# Supplementary material for: Inducible plasmid copy number control for synthetic biology in commonly used E. coli strains
Source: Nat Commun. 2022 Nov 5;13:6691. doi: 10.1038/s41467-022-34390-7 (PMC9637173; doi:10.1038/s41467-022-34390-7)
Supplement: Supplementary file 1 — Supplementary Information [file 41467_2022_34390_MOESM1_ESM.pdf]

Supplementary Information for “Inducible plasmid copy number control for synthetic biology in commonly used *E. coli* strains”

Shivang Hina-Nilesh Joshi,[1] Chentao Yong,[1,2] Andras Gyorgy[1*]

[1]Division of Engineering, New York University Abu Dhabi, United Arab Emirates
[2]Department of Chemical and Biomolecular Engineering,
New York University, New York, NY USA
*To whom correspondence should be addressed; E-mail: andras.gyorgy@nyu.edu.

This Supplementary Information includes (i) additional experimental data; (ii) complete list of strains, genetic parts, constructs, key primers, and CRISPRi guide RNA designs used in the paper; and (iii) details on the mathematical model and analysis complementing the experimental data. Across the document the following abbreviations are used: “PCN” for plasmid copy number; *E. coli* for *Escherichia coli*; “NonFP” for non-fluorescent *E. coli* cells carrying no plasmid; “OD” for Optical Density; “PBS” for filter sterilized phosphate buffered saline. Black lines denote the population median in histograms, and gray infills and dashed-lines indicate data corresponding to NonFP samples.

Contents

1 Supplementary experimental data **3**

1.1 Reconfiguring the pSC101 origin for inducible PCN control 3

1.2 Implementing inducible PCN control on a single plasmid 3

1.3 TULIP is portable across a variety of *E. coli* strains 9

1.4 PCN can be dynamically regulated and reliably maintained using TULIP . . 13

1.5 TULIP reduces our reliance on cloning and transformation 17

1.6 TULIP promotes the re-use of modules in different contexts 18

1.7 TULIP can facilitate gene circuit prototyping 19

1.8 TULIP enables versatile PCN control using a variety of input stimuli 31

1.9 Gating cells in flow cytometry experiments 32

1.10 Kinetic microplate reader data 32

2 Strains, genetic parts, constructs, and primers **71**

3 Mathematical model and analysis **87**

3.1 Single layer sensor modules and NOT gate 87

3.2 Toggle switch 89

3.3 Typical range of model parameters 93

1 Supplementary experimental data

Here, we present the supplementary experimental data accompanying the main results presented in the manuscript, together with details on flow cytometry gating, and alongside with growth rate and timecourse data for kinetic microplate reader experiments.

1.1 Reconfiguring the pSC101 origin for inducible PCN control

The fPCN collection of plasmids with fixed copy number (*1*) consists of 8 plasmids (Supplementary Table 2), offering more than 20-fold control over gene expression across a PCN range spanning 3 to 62 copies per cell with minimal effect on growth rate in NEBStable (Supplementary Fig. 1).

1.2 Implementing inducible PCN control on a single plasmid

Implementation of TULIP on a single plasmid was carried out in two main steps: (i) RBS library preparation and initial selection; and (ii) screening for inducible PCN behavior.

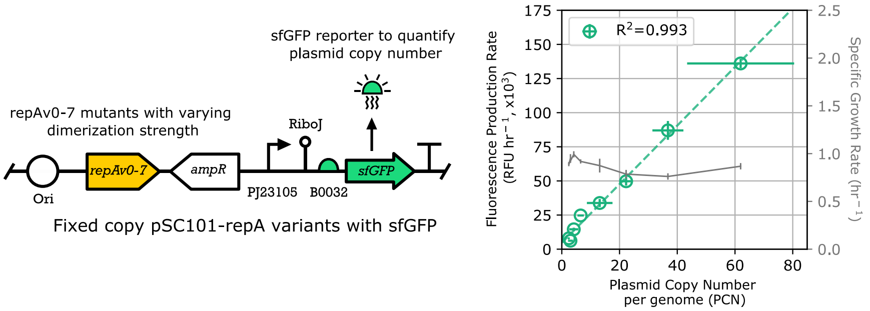

Supplementary Fig. 1. **Plasmids with fixed copy number (fPCN collection) offer substantial control over both PCN and gene expression rate.** Fluorescence production rate (green) and growth rate (gray) were measured via kinetic microplate reader experiments, PCN was quantified using qPCR.

1.2.1 RBS library preparation and initial selection

In order to maximize efficiency for library cloning and to ensure that all possible variants are assembled, the RBS library was constructed in a 2-part Golden Gate assembly reaction. An intermediary "base" plasmid (pSJ10) was constructed to serve as the library backbone, carrying the sfGFP cassette, ampicillin resistance marker, and the incomplete synthetic origin for inducible PCN control (Supplementary Fig. 2). For stable maintenance of the plasmid despite the incomplete origin, a pMB1 high-copy origin is used initially, then later removed by PCR amplification during the library construction.

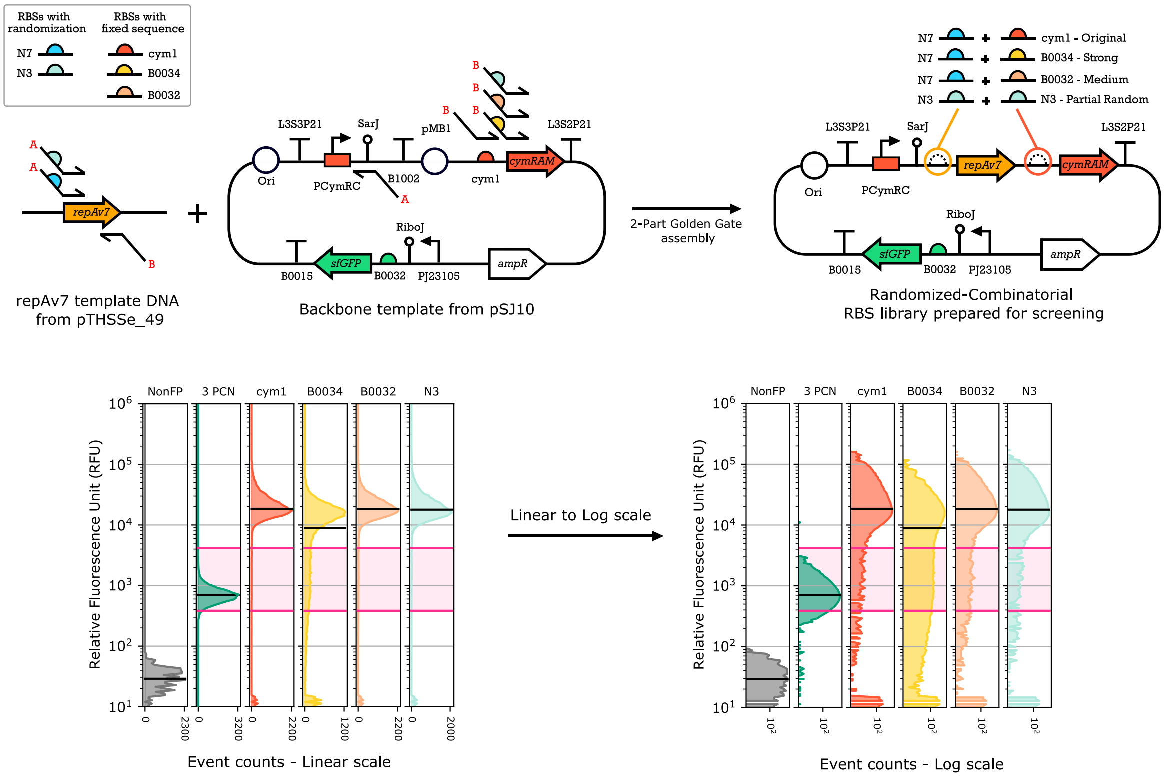

Supplementary Fig. 2. **RBS library construction and initial screening.** Non-fluorescent cells (NonFP) and pTHSSe_42 carrying cells (3 PCN) act as negative and positive controls. The four libraries constructed are labeled cym1 (N7-cym1), B0034 (N7-B0034), B0032 (N7-B0032), and N3 (N3-N3). Black lines denote the population median, magenta region highlights the population fluorescence intensity of interest.

The variant repAv7 was amplified from pTHSSe_49 (*1*), with the forward primer overhang carrying the Shine-Dalgarno sequence with degenerate nucleotides (N7 or N3) and BsaI sites generating the sticky end denoted by A at the 5'. The reverse primer overhang only has a BsaI site generating the sticky end denoted by B at the 3'. For the backbone, the forward primer either targets the complete cym1 RBS, or the CymRAM coding sequence carrying a predefined RBS (B0034 or B0032), or the degenerate Shine-Dalgarno (N3) in the overhang; the reverse primer targets the SarJ ribozyme sequence. Both primers for the backbone amplification carry BsaI restriction sites generating sticky ends B and A at the 5' and 3' ends, respectively. The primers used for the library construction can be found in Supplementary Table 9. The libraries were transformed into NEBStable cells, grown overnight on solid media without any inducer, and analyzed using a BD FACSAria. The resulting population distributions across all libraries show a significant bias for high PCN variants in the absence of Cuminic acid (Supplementary Fig. 2). Scaling the event counts to a logarithmic scale reveals the N7-B0034 library as the best candidate with variants within the desired initial low PCN range (Supplementary Fig. 2).

1.2.2 Screening for inducible PCN behavior

Following library preparation and initial selection, cells screened on day 1 were cultured on LB agar plates induced with a range of Cuminic acid concentrations in preparation for the second round of screening. For this next round, non-fluorescent cells (NonFP) were considered as the negative controls, and cells carrying pTHSSe_42 (3 PCN) and pTHSSe_49 (62 PCN) sfGFP expressing plasmids as the positive controls.

We initially planned to alternate the absence and presence of Cuminic acid inducer in each round of flow cytometry screening. To this end, a range of concentrations were tested (0, 0.5, 1.0, and 1.5 μM). When samples were analyzed using flow cytometry (Supplementary Fig. 3a), we observed that the population in the absence of any inducer resembled that of day 1. In the

presence of Cuminic acid the population median did increase, and this shift correlated with the inducer concentration, suggesting that the library did contain PCN inducible variants.

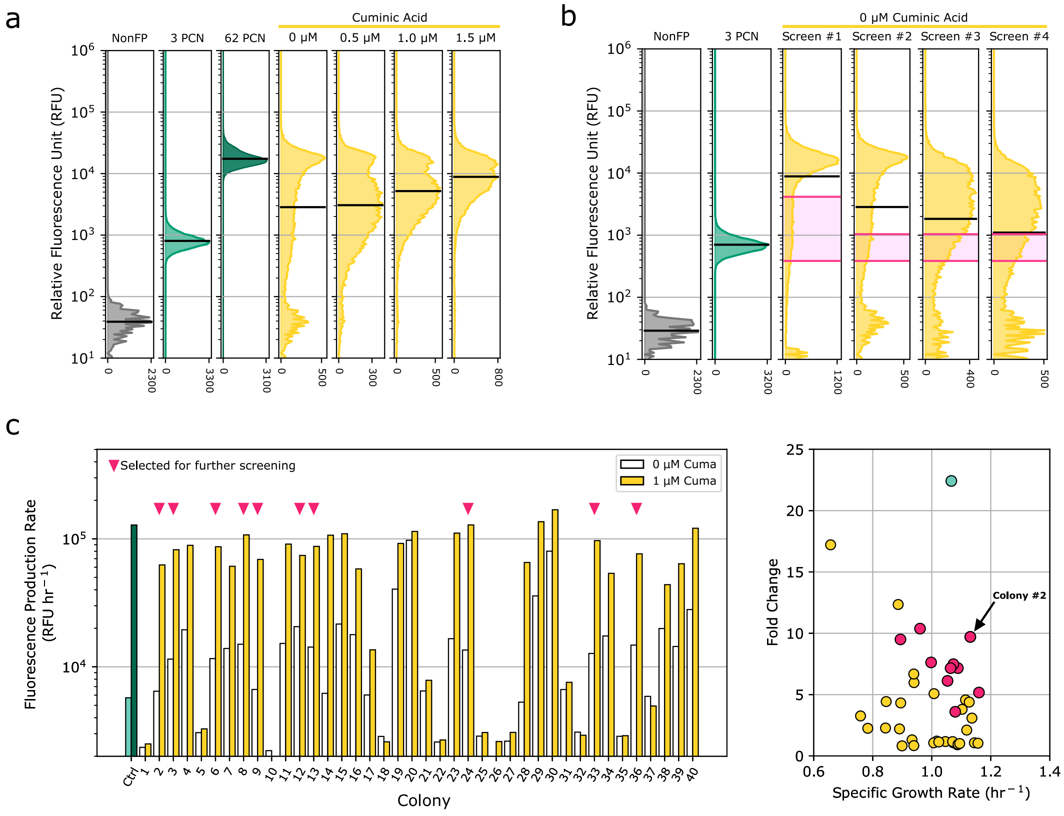

Supplementary Fig. 3. **Screening of the N7-B0034 library.** Non-fluorescent cells (NonFP) are the negative, and cells carrying pTHSSe_42 (3 PCN, light green) and pTHSSe_49 (62 PCN, dark green) are the positive controls for fluorescence. **a** Cells selected from screen #1 of the N7-B0034 library were cultured overnight on LB agar plates with either 0, 0.5, 1.0, or 1.5 μM Cuminic acid. Black lines denote the population median. **b** For subsequent rounds of screening from the N7-B0034 library, cells were gated from the low fluorescence intensity range with progressively narrower gates for screens #2–#4, indicated by the magenta regions. **c** After screen #4, 40 colonies were manually selected by eye and screened for PCN inducibility in microplate reader experiments (both in the absence and presence of 1μM Cuminic acid, using sfGFP signal per cell as the proxy for PCN). The scatter plot displays data about these 40 colonies. Fold change refers to the ratio of fluorescence in the presence and absence of Cuminic acid (for positive controls it is the ratio of pTHSSe_49 and pTHSSe_42), growth rate is the averaged growth rate in the presence and absence of Cuminic acid (for positive controls it is the average of pTHSSe_42 and pTHSSe_49). Magenta indicates colonies selected for further screening, green corresponds to the positive control.

Selecting for the high PCN state in this second round would have been futile, as even in the absence of inducer there were still many constitutively high copy variants that would dilute out and compete against the inducible variants of interest. To address this issue, cells in the low fluorescence intensity range were gated in subsequent rounds of flow cytometry screening and a more stringent gate width was used. This resulted in progressively decreasing population median with each round (Supplementary Fig. 3b). Finally, 40 colonies were selected from the day 4 plate, and then cultured in microplate reader experiments, both in the presence and absence of 1 μM Cuminic acid (Supplementary Fig. 3c). Considering both their growth rate (comparable to the positive control) and their responsiveness to Cuminic acid (indicating inducible PCN behaviour), 10 colonies were selected for further screening (Supplementary Fig. 3c).

These 10 candidate variants were further screened by using a Cuminic acid gradient (0, 0.1, 0.2, 0.4, 0.8, 1.6, 2.4, and 4.0 μM). This revealed that all variants responded to Cuminic acid induction (Supplementary Fig. 4). Unfortunately, all candidates displayed a broad distribution at low concentrations of the inducer (or in its absence), many spanning more than 2-orders of magnitude (e.g., colony #2, #6, #13). This, taken together with the presence of bimodal distributions (e.g., colony #6, #8, #9, #12, #24, #36) indicated the presence of non-fluorescent cell populations, thus revealing the need for further optimization of the circuit.

Considering its broad dynamic range and the almost complete absence of non-fluorescent sub-population, colony #2 was selected for further optimization (Supplementary Fig. 4). To reduce the relatively high PCN in the absence of the inducer due to the potential accumulation of RepAv7, either a weak (ASV) or a moderate-weak (AAV) degradation tag (*2*) was attached to RepAv7 to promote its active removal. Both these modifications resulted in significantly narrower distributions at lower inducer concentrations than the original non-tagged variant (Supplementary Fig. 5). As there was no noticeable difference between the two variants, the version with the moderate-weak (AAV) tag was selected for subsequent experiments.

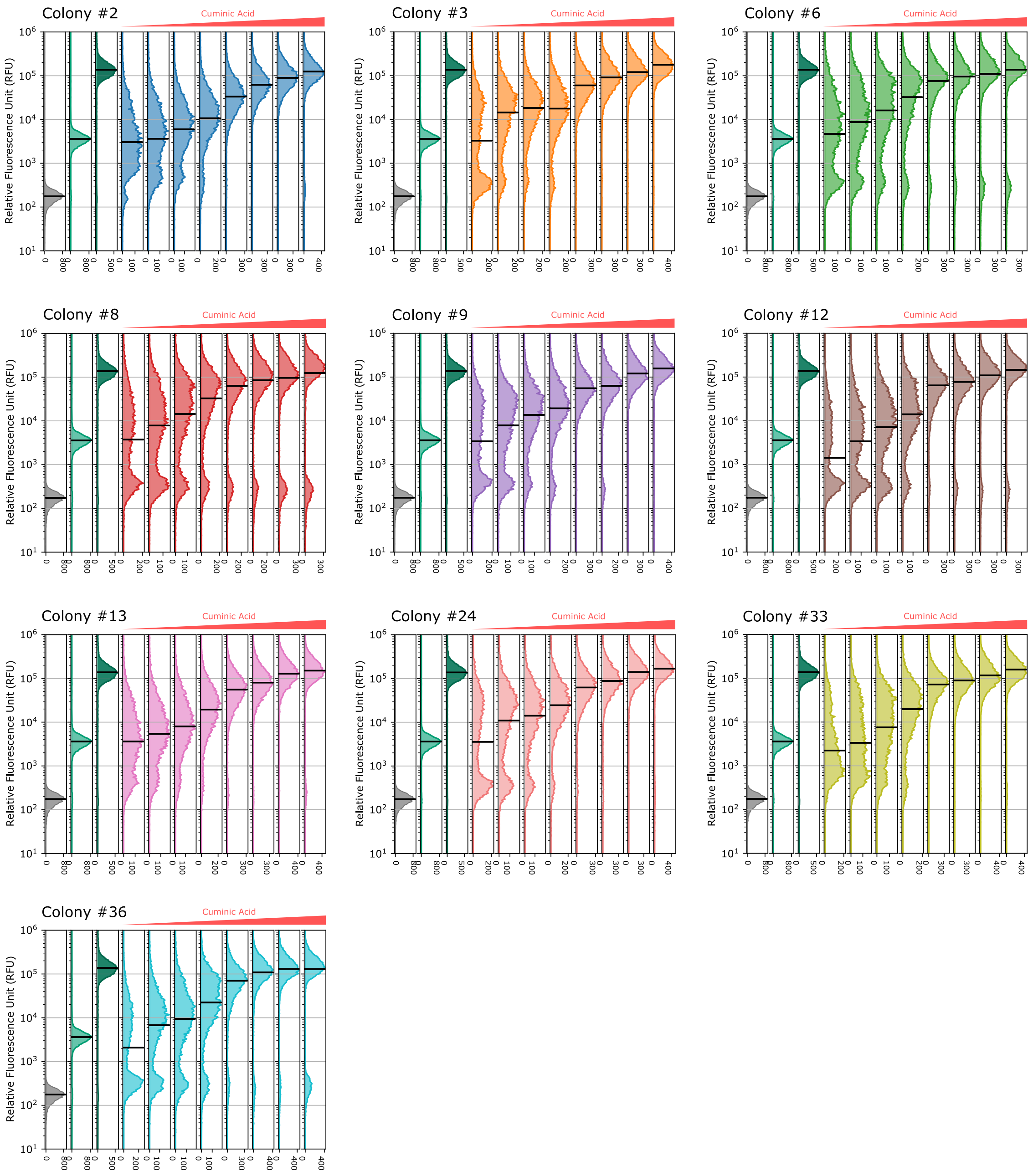

Supplementary Fig. 4. **Screening of the 10 candidate variants selected in Supplementary Fig. 3c for response to Cuminic acid induction.** Non-fluorescent cells (NonFP, gray) are the negative controls, and cells carrying pTHSSe_42 (3 PCN, light green) and pTHSSe_49 (62 PCN, dark green) are the positive controls. Each of the 10 variants selected were induced with Cuminic acid at the following concentrations: 0, 0.1, 0.2, 0.4, 0.8, 1.6, 2.4, and 4.0 μM.

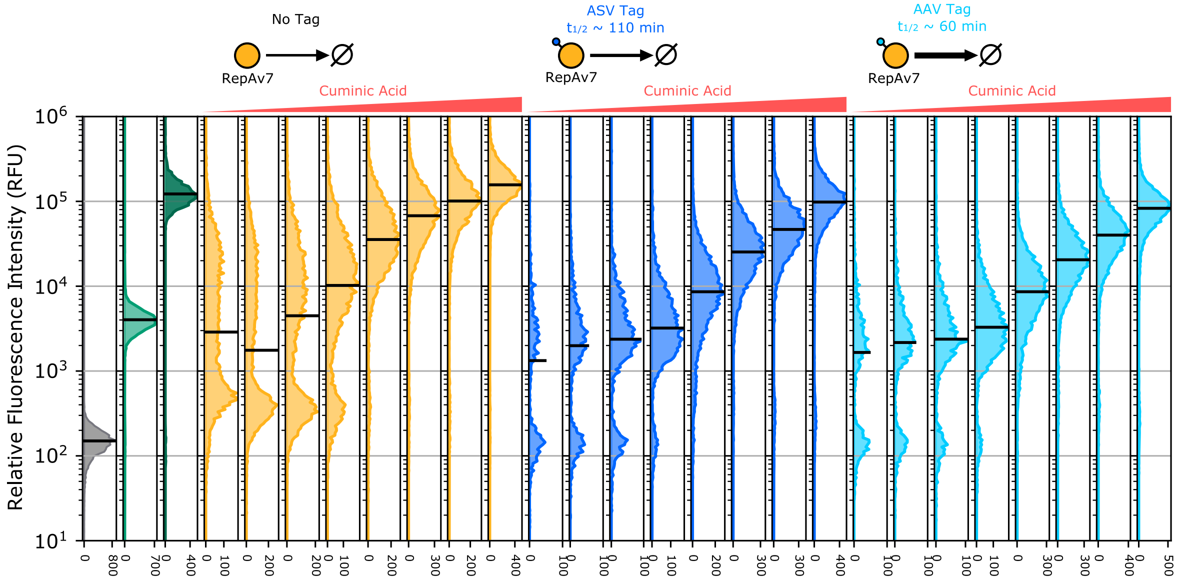

Supplementary Fig. 5. **Screening of colony #2 with and without degradation tags in NEB-Stable cells.** Non-fluorescent cells (NonFP, gray) are the negative controls, and cells carrying pTHSSe_42 (3 PCN, light green) and pTHSSe_49 (62 PCN, dark green) are the positive controls. Light-orange populations carry the colony #2 plasmid with repAv7, blue populations have repAv7-ASV plasmids, and cyan populations have repAv7-AAV plasmids. The cells were induced with Cuminic acid at the following concentrations: 0, 0.1, 0.2, 0.4, 0.8, 1.6, 2.4, and 4.0 μM.

1.3 TULIP is portable across a variety of *E. coli* strains

Flow cytometry data presented in Supplementary Fig. 6 collected across a variety of *E. coli* strains reveal three key observations about the behavior of TULIP. First and foremost, the data confirm that PCN can be modulated via the addition of Cuminic acid across a wide variety of *E. coli* strains as the TULIP carrying cell population distribution is distinct at each inducer level and TULIP's inducible PCN function is retained in all 5 tested strains (Figures 2–3). Second, NEBStable, DH10B, and BW25113 cells with no induction of Cuminic acid were indistinguishable in fluorescence from the negative non-fluorescent controls, whereas NEBExpress and MG1655 displayed bimodality in the absence of Cuminic acid (Supplementary Fig. 5 and Sup-

plementary Fig. 6). As a result, all subsequent solid culture medium and cloning liquid cultures were supplemented with 1 μM Cuminic acid for stable maintenance of the plasmid. Third, at low Cuminic acid concentrations the population-level distribution of relative fluorescence intensity appears broader than for the fPCN collection. This could stem from the timescale difference of the feedback mechanism: for the pSC101 variants the negative feedback occurs at the post-translational level where removal of monomeric RepA and transcription inhibition of repA expression is simultaneous; whereas for TULIP at the transcriptional level, and excess RepAv7 removal is via dilution and degradation.

In addition to M9-Glucose (M9-Gluc), we also induced TULIP to a range of PCNs in a variety of growth media commonly used in experimental microbiology: M9-Glycerol (M9-Gly), Lysogeny Broth (LB), and Super Optimal Broth (SOB). The data in Supplementary Fig. 7 ver-

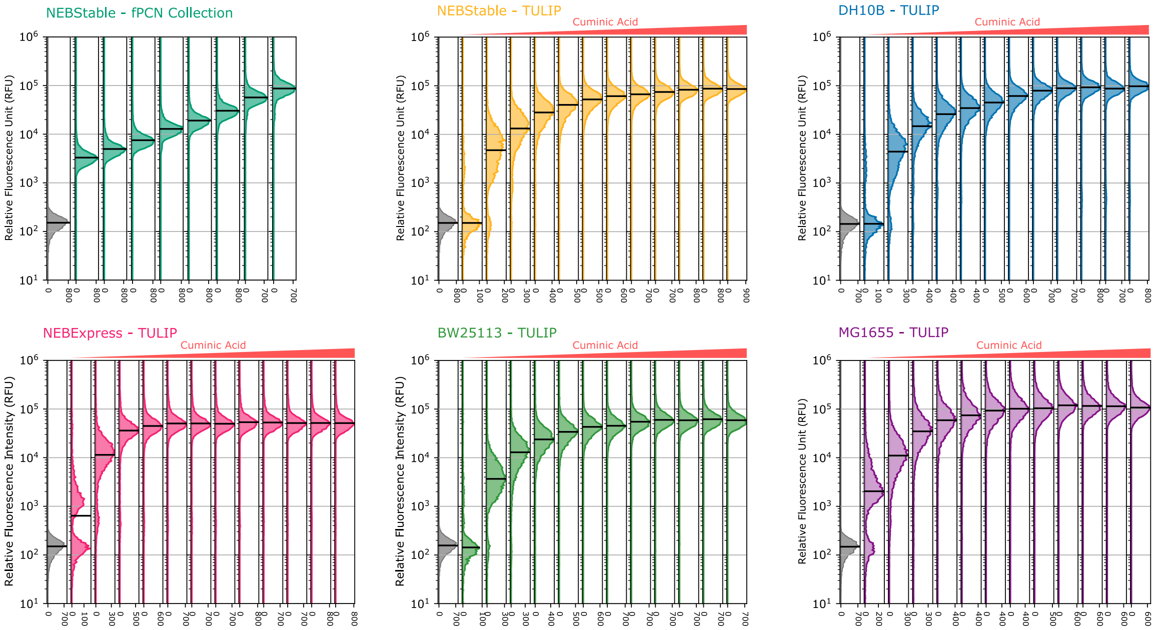

Supplementary Fig. 6. **Flow cytometry histograms of fPCN in NEBStable and TULIP in multiple strains of *E. coli*.** Non-fluorescent cells (NonFP, gray) are the negative controls. TULIP was induced with Cuminic acid at 0, 0.4, 0.8, 1.2, 1.6, 2.0, 2.4 3.0, 4.0, 5.0, 7.5, and 10.0 μM.

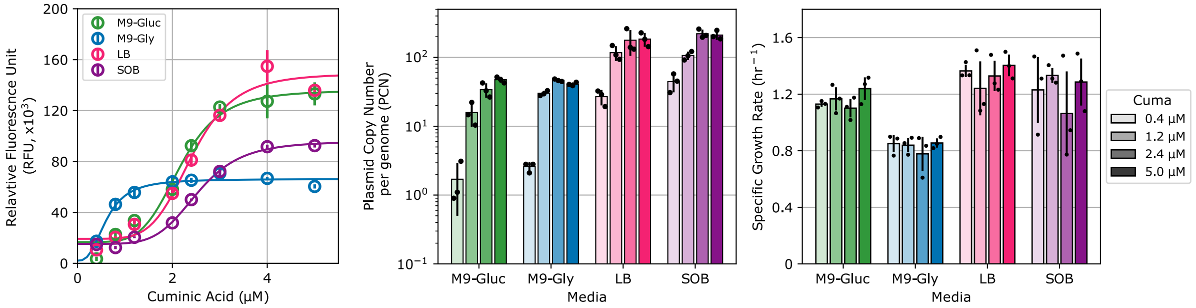

Supplementary Fig. 7. **TULIP enables flexible PCN control across a variety of growth media.** The empty TULIP backbone was transformed into DH10B cells and PCN inducibility was characterized using flow cytometry and qPCR in M9-Glucose (M9-Gluc), M9-Glycerol (M9-Gly), Lysogeny Broth (LB), and Super Optimal Broth (SOB). An experiment with identical media conditions was set up in a microplate reader to characterize growth rate. Each experiment was conducted in biological triplicates ($n = 3$), data points and error bars represent the mean and standard deviation across replicates.

ify not only that TULIP enables inducible PCN control in these other growth media as well, but also confirm that its presence has negligible impact on growth rate across all tested media and Cuminic acid concentrations. Additionally, while cells in M9-Gly grow significantly slower than those in M9-Gluc (considering the approximately 33% reduction in growth rate from $\sim$1.2 h^{-1} to $\sim$0.8 h^{-1}), the measured PCN dynamic range is almost identical (2–48 in M9-Gluc and 3–47 in M9-Gly). While growth rate differences in M9-Gluc and M9-Gly seem to have negligible impact on PCN, they likely represent a major contributor to the observed differences in gene expression: e.g., at the highest tested concentration flow cytometry analysis reveals that sfGFP expression in M9-Gluc is approximately 2.2-fold higher than in M9-Gly, despite the nearly identical PCN values. All subsequent experiments were performed in M9-Gluc media due to its low autofluorescence when compared to other “rich” media alternatives.

While our results confirm that TULIP can be successfully deployed for flexible PCN control across a wide range of *E. coli* strains, behavior in these hosts display considerable variation from a quantitative perspective (*3*). This is illustrated, for instance, by considering the observed PCN range, spanning approximately 2–38 in DH10B, 3–51 in NEBStable, 29–82 in NEBEx-

press, 2–102 in BW25113, and 13–184 in MG1655 (Figure 3b, Supplementary Fig. 6). This heterogeneity likely stems from variable protein expression rates due to the differences in genetic profiles and in the availability of the DNA replication proteins provided by the host that are required for successful replication using the pSC101 origin (e.g., DnaA, DnaB, and DnaG (*4*)). Therefore, we expect the behavior of TULIP to be similar when considering strains that are closely related, and that differences should increase with phylogenetic distance. This is confirmed, for instance, when comparing the heavily mutated NEBStable and DH10B strains to the

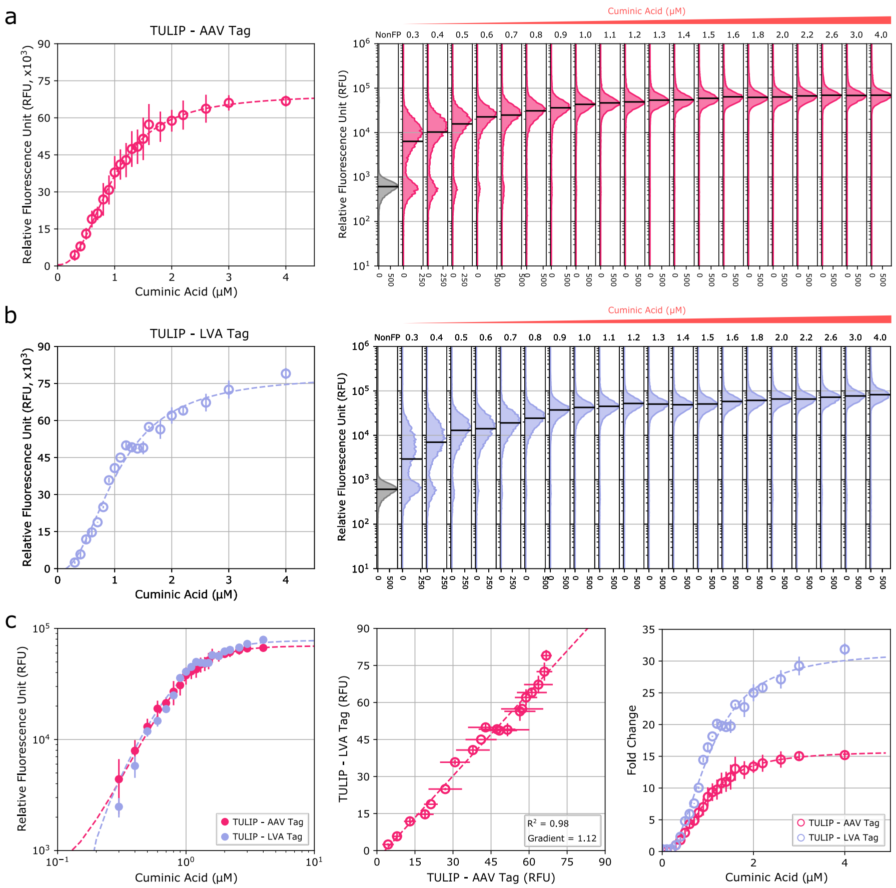

Supplementary Fig. 8. **Performance of TULIP in NEBExpress.** PCN is modulated via the addition of Cuminic acid. **a** TULIP with the moderate AAV tag. **b** TULIP with the strong LVA tag. **c** Comparison of TULIP's performance with the two degradation tags. Each experiment was conducted in biological triplicates ($n = 3$), data points and error bars represent the mean and standard deviation across replicates.

BW25113 and MG1655 cells, which are close relatives of the WT strain carrying almost the complete repertoire of *E. coli* genes.

The response of TULIP to Cuminic acid in NEBExpress appears considerably less gradual than in the other strains when using the same inducer concentration gradient, however, this issue can be easily mitigated by considering a finer concentration gradient of Cuminic acid (Supplementary Fig. 8a). This phenomenon likely stems from the lack of Lon and OmpT protease machinery in NEBExpress (*5*), contributing to a lower removal rate of RepAv7, thus increasing sensitivity of PCN to lower levels of Cuminic acid when compared to the other strains. To increase the removal rate of RepAv7, we modified TULIP by replacing the moderate AAV degradation tag with a stronger LVA tag (Supplementary Fig. 8b). This modification lowered the minimum PCN that can be stably maintained, improving the dynamic range of TULIP in NEBExpress from 15-fold to 32-fold (Supplementary Fig. 8c), however, TULIP's dynamic performance to Cuminic acid induction appears nearly identical with the two degradation tags.

1.4 PCN can be dynamically regulated and reliably maintained using TULIP

To demonstrate dynamic PCN regulation, DH10B cells harboring the empty TULIP backbone were grown using the Chi.Bio turbidostat platform (*6*) to ensure their continuous exponential growth. Fresh media supplemented with varying levels of Cuminic acid was exchanged every 12 hours, alongside with sampling for flow cytometry analysis. The data in Supplementary Fig. 9a confirm that multiple setpoint changes in both directions can be achieved via modulating Cuma concentration in the fresh media, and that these setpoints can be reliably maintained for over 10 generations each. Since specific growth rate is approximately 1–1.2 h^{-1} independent of PCN (Figure 4a), it takes approximately 3–4 hours to replace over 99% of the media and to switch between consecutive setpoints. Flow cytometry analysis in Supplementary Fig. 10a

confirms that this is the typical timescale for cells to reach the new equilibrium subsequent to instantaneous changes in Cuminic acid concentration.

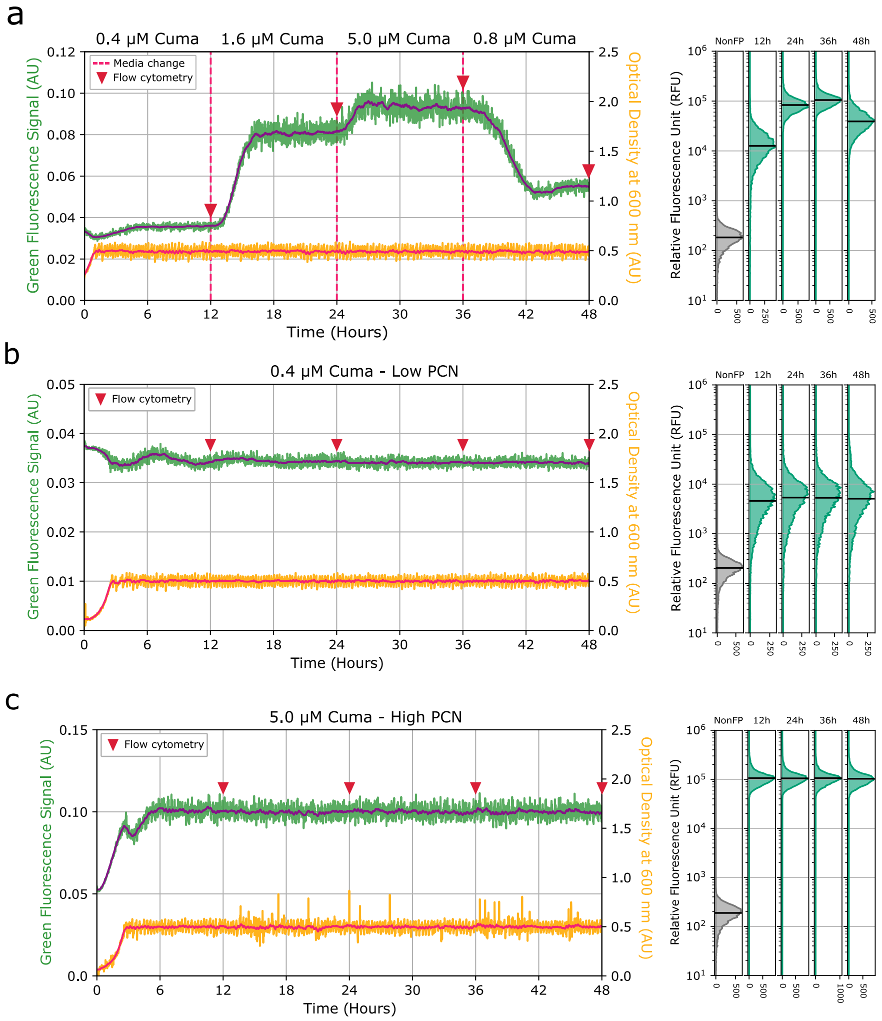

Supplementary Fig. 9. **Long-term turbidostat experiments using the Chi.Bio platform (*6*) with DH10B cells harboring the empty TULIP backbone.** OD600 is maintained at constant level (orange) to ensure that cells are kept at exponential phase by simultaneously removing media containing cells and pumping in fresh media. Green and orange represent raw data, purple and red overlays represent 30-point moving average to highlight the key features of the trace data. Flow cytometry samples were taken at 12 hour intervals to verify plasmid stability. **a** Fresh media was changed every 12 hours, successive batches contained Cuminic acid at 0.4 μM, 1.6 μM, 5.0 μM, and 0.8 μM concentration. **b** Fresh media contained Cuminic acid at 0.4 μM concentration throughout the entire experiment. **c** Fresh media contained Cuminic acid at 5.0 μM concentration throughout the entire experiment.

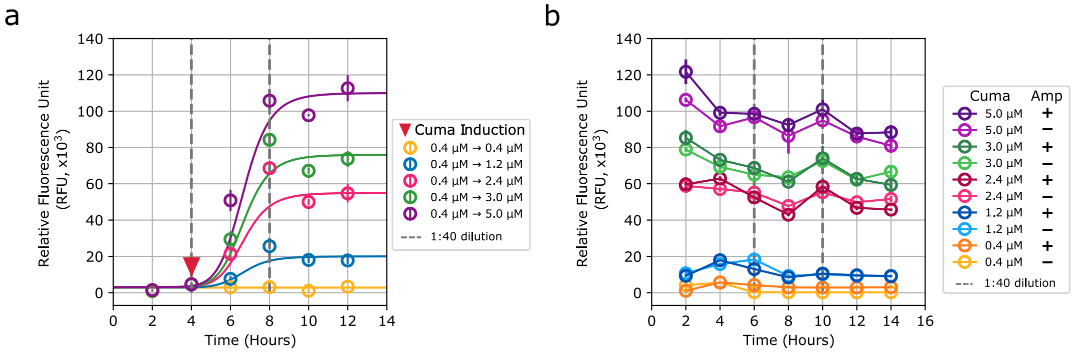

Supplementary Fig. 10. **Flow cytometry analysis of plasmid setpoint change and plasmid stability experiments using DH10B cells harboring the empty TULIP backbone.** Samples were taken every 2 hours to quantify sfGFP expression as proxy for PCN and to determine plasmid stability across the population. **a** Cells were cultured in 0.4 μM Cuminic acid for the overnight pre-culture and for the first 4 hours of the experiment. At 4 hours, PCN was upregulated by transferring cells to fresh media with an elevated target Cuminic acid concentration. The corresponding representative flow cytometry histograms are presented in Supplementary Fig. 11. **b** Cells were cultured overnight in media with varying levels of Cuminic acid and in the presence of ampicillin. At 0 hours, cells were diluted 1:200 into fresh media (maintaining Cuminic acid concentration), either containing or lacking ampicillin. The corresponding representative flow cytometry histograms are presented in Supplementary Fig. 12.

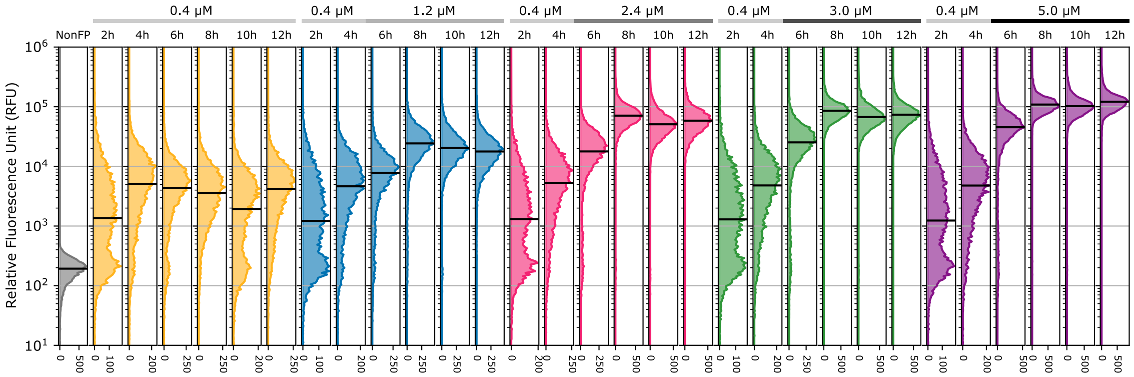

Supplementary Fig. 11. **Representative flow cytometry histograms for plasmid induction experiment presented in Supplementary Fig. 10a using the same color scheme.** Cuminic acid concentration in the growth media is indicated above the histograms.

Next, we repeated the Chi.Bio turbidostat experiment with media containing either 0.4 μM or 5.0 μM Cuminic acid, corresponding to the lowest and highest reliably controllable PCN in

DH10B, respectively. The results in Supplementary Fig. 9bc confirm that PCN can be reliably maintained with TULIP for extended periods of time (over 50 generations) with negligible temporal variation in the presence of antibiotic selection pressure, whereas in the absence of ampicillin, TULIP is gradually lost over time, especially at low Cuminic acid concentrations (Supplementary Fig. 10b, Supplementary Fig. 12).

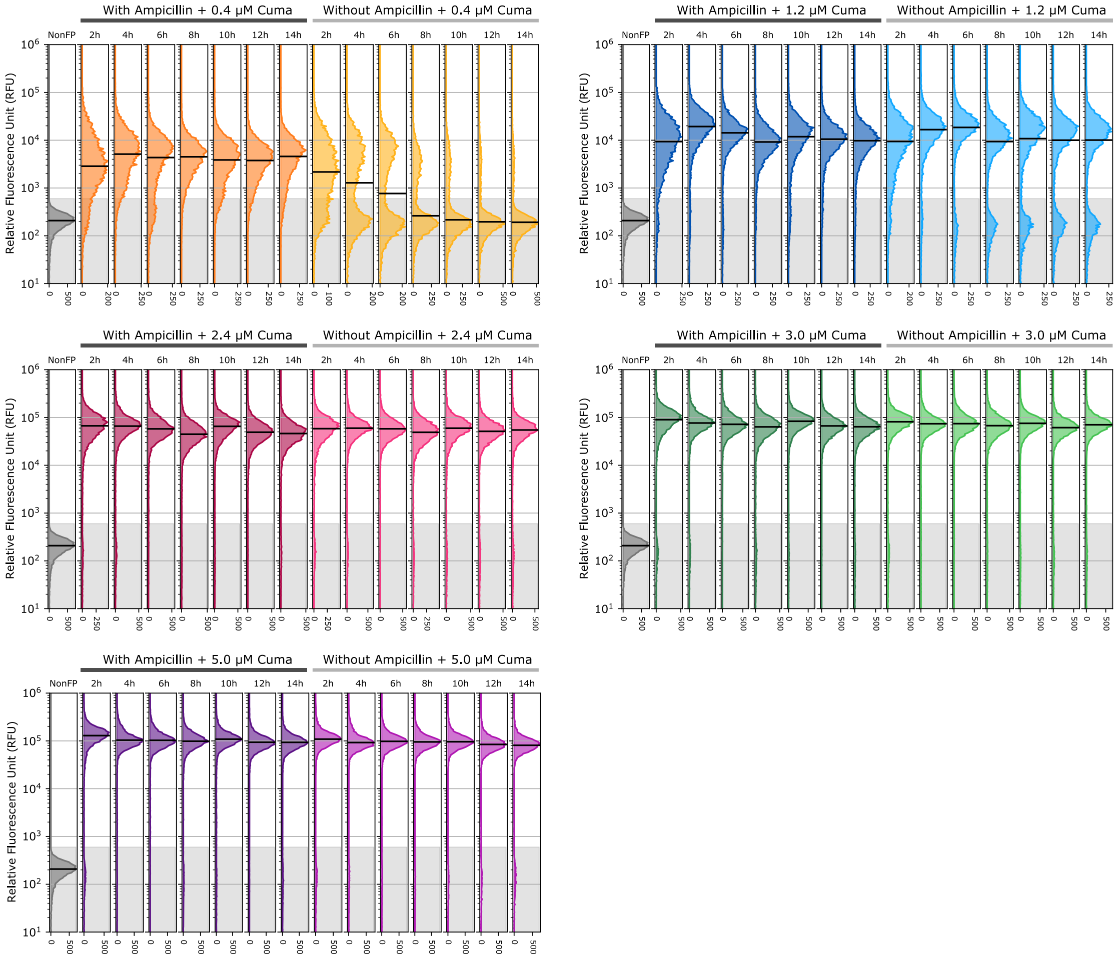

Supplementary Fig. 12. **Representative flow cytometry histograms for plasmid stability experiment both with and without ampicillin selection corresponding to Supplementary Fig. 10b using the same color scheme.** Plasmid loss is concluded if sfGFP expression falls below the threshold defined by non-fluorescent cells (gray).

1.5 TULIP reduces our reliance on cloning and transformation

To reveal the impact of competition for shared cellular resources on PCN when using TULIP, we selected the sensor module in Figure 5b as an illustrative example since Van-induced mScarI expression represents considerable metabolic burden, leading to an approximately 75% reduction in sfGFP expression (Figure 5b). According to the data presented in Supplementary Fig. 13 considering both TULIP and plasmids with fixed copy number, increased metabolic burden has minor impact on the PCN of either type of plasmid vector despite the significant drop in sfGFP expression. In case of TULIP, the only potential exception may be at 0.8 μM Cuminic acid concentration, where PCN decreases from approximately 5 to 3 with Vanillic acid induction. However, such a relative change can be observed in the opposite direction in case of p15A (from approximately 5 to 10), suggesting that these fluctuations at low PCN levels may be independent of Van induction.

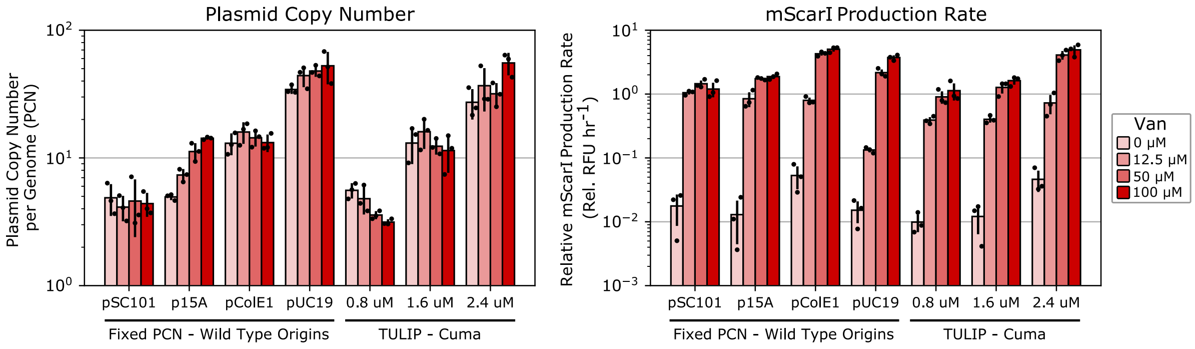

Supplementary Fig. 13. **PCN (obtained via qPCR) and mScarI production rate (obtained via microplate reader experiments) considering the Van-inducible sensor module from Figure 5b cloned into plasmids with fixed copy number and TULIP.** Gene expression load is modulated via induction with Vanillic acid at different concentrations. TULIP is induced with 0.8 μM, 1.6 μM, and 2.4 μM Cuminic acid. All experiments were carried out in DH10B. Each experiment was conducted in biological triplicates ($n = 3$), data points and error bars represent the mean and standard deviation across replicates.

1.6 TULIP promotes the re-use of modules in different contexts

Complementing the results presented in Figures 5–6, consider the multi-layer sensor module in Supplementary Fig. 14, obtained by placing the AmtR gene (inhibitory TF) downstream of the PVanCC promoter in the B0032-VanRAM circuit from Figure 5b, together with the mScarI reporter under the PAmtR promoter. Thus, mScarI is constitutively expressed in the absence of Vanillic acid, when the AmtR gene is inactive. Upon induction with Vanillic acid, expression of AmtR leads to mScarI inhibition, thus the module in Supplementary Fig. 14 implements a NOT gate. For all tested PCN values (light red: 5/18, medium red: 12/50, dark red: 23/72 in DH10B/BW25113) gene expression decreased as an inverse Hill curve with increasing concentrations of Vanillic acid, confirming that the multi-layer genetic circuit functions as expected.

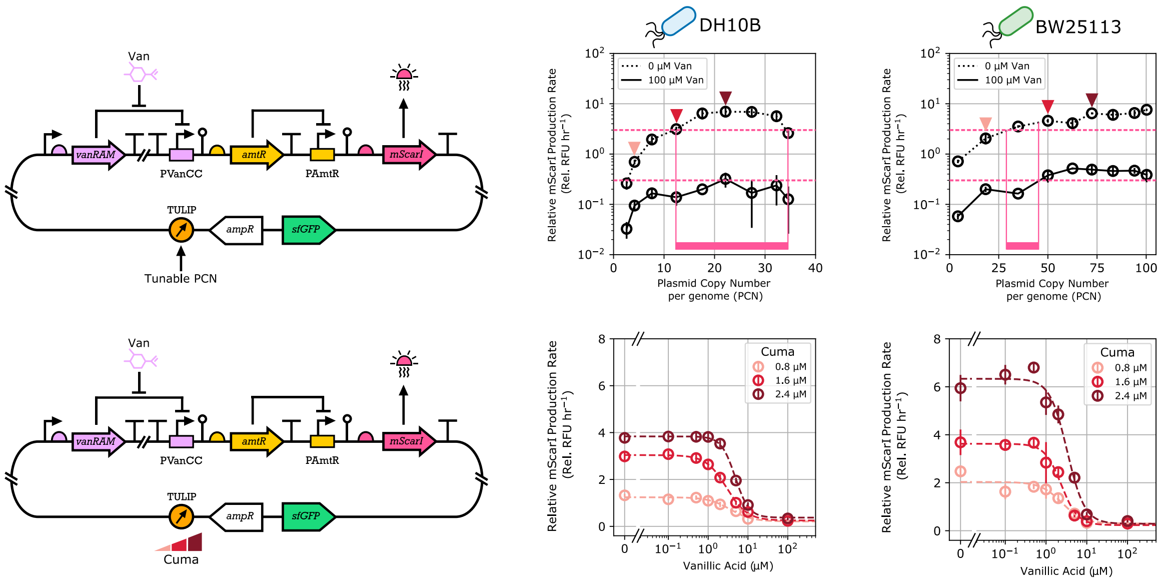

Supplementary Fig. 14. **Behavior of a genetic NOT gate harbored in TULIP, both in DH10B and BW25113.** Expression of mScarI is monitored in microplate reader experiments. Arrowheads correspond to the following Cuminic acid concentrations: 0.8 μM (light red), 1.6 μM (medium red), and 2.4 μM (dark red). Horizontal bars indicate the PCN range where the output in the ON/OFF states is greater/lower than the critical threshold (10-fold range) denoted by the dashed magenta lines. Fluorescence production rate is normalized against a mScarI control strain (DHScarI or BWScarI) for each respective strain of *E. coli* (Supplementary Table 1).

Importantly, the ON/OFF states (corresponding to 0 μM and 100 μM Vanillic acid induction, respectively) in one strain can be easily matched in the other: for instance, by changing Cuminic acid concentration from 2.4 μM in DH10B to 1.6 μM in BW25113, without any cloning or transformation steps. The dynamic range can also be tuned in a straightforward fashion, increasing it from 6-fold to 40-fold in DH10B, and from 7-fold to 20-fold in BW25113. The data in Supplementary Fig. 14 also illustrate how PCN can be easily adjusted via TULIP so that the output in the ON/OFF state is greater/lower than a critical threshold value (denoted by dashed magenta lines in Supplementary Fig. 14). This can be leveraged, for instance, to ensure compatible input/output dynamic ranges in multi-layered genetic circuits (*7, 8*), especially considering that the ON state could be increased by almost 30-fold in DH10B without significantly affecting the K_m value of the tested versions (approximately 3.75±0.95 μM Vanillic acid).

1.7 TULIP can facilitate gene circuit prototyping

Complementing the results presented in Figure 7, in Supplementary Fig. 15 we include representative histograms for both pre-wash and post-wash samples (alongside with the corresponding eGFP-mKate2 scatter plots in Supplementary Fig. 16, Supplementary Fig. 17, and Supplementary Fig. 18) for experiments carried out using DH10B cells harboring the toggle switch and with chromosomal LacI expression. In Supplementary Fig. 19 we present the fraction of cells that are in the "green state" (high TetR expression, low LacI expression after IPTG induction) or in the "red state" (high LacI expression, low TetR expression after aTc induction) for both the pre-wash and post-wash samples. Except at the lowest PCN levels, the overwhelming majority of cells preserve their steady state following the removal of the inducers (IPTG/aTc), certifying bistability independent of the backbone once PCN is sufficiently high. In Supplementary Fig. 20–Supplementary Fig. 25 we include the results following the same protocol and using the same constructs, but this time with DH10B cells lacking chromosomal LacI expression.

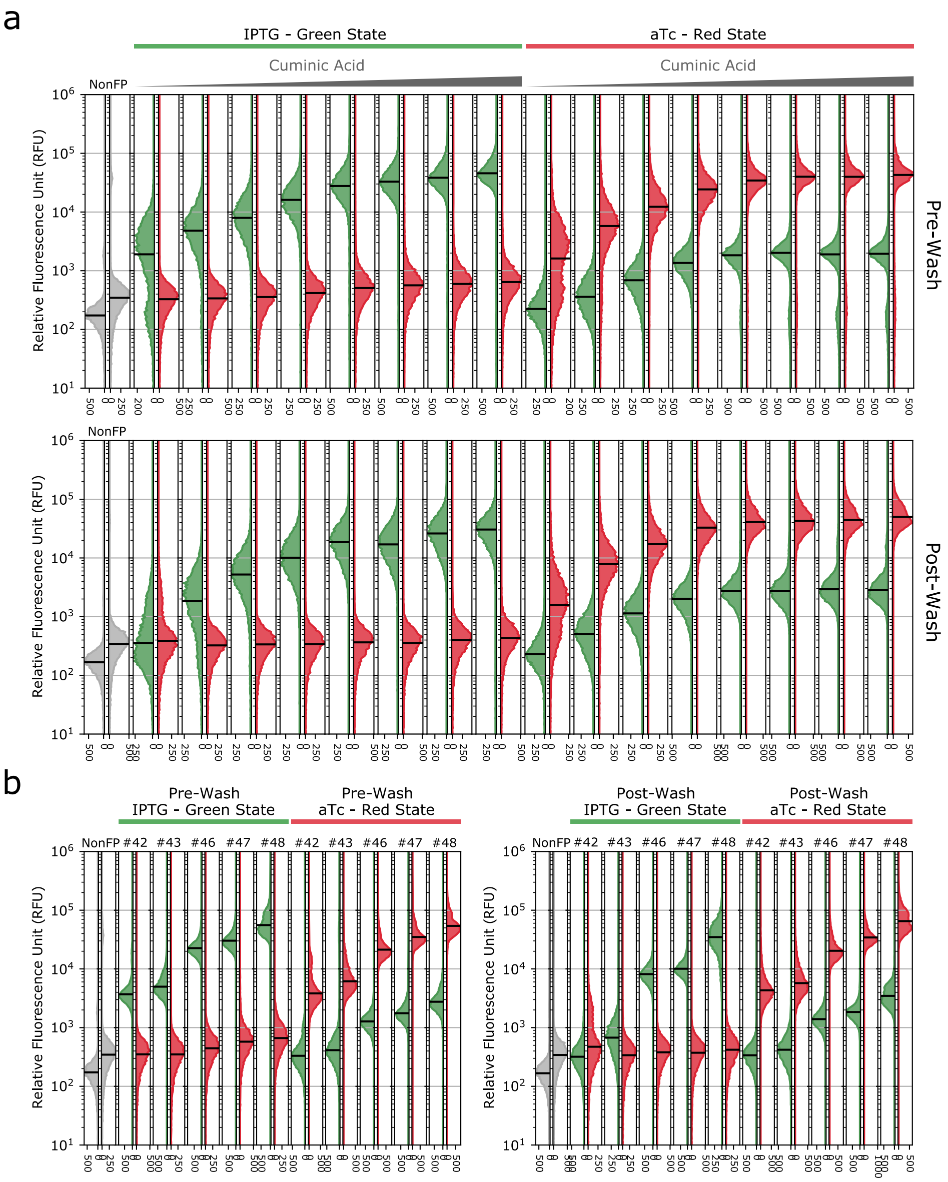

Supplementary Fig. 15. **Representative histograms for the toggle switch obtained in the experiments corresponding to Figure 7 considering DH10B cells with chromosomal LacI expression.** Pre-wash and post-wash refer to samples taken at 8 h and at 32 h, respectively, see Figure 7a for more details. Each diverging pair of x-axis depicts data from a single sample, the left side axis represents fluorescence signal distribution in the eGFP channel, the right side axis presents signal distribution in the mKate2 channel. **a** Toggle switch is harbored in TULIP, PCN is modulated via the addition of Cuminic acid. **b** Toggle switch is harbored in plasmids with fixed PCN.

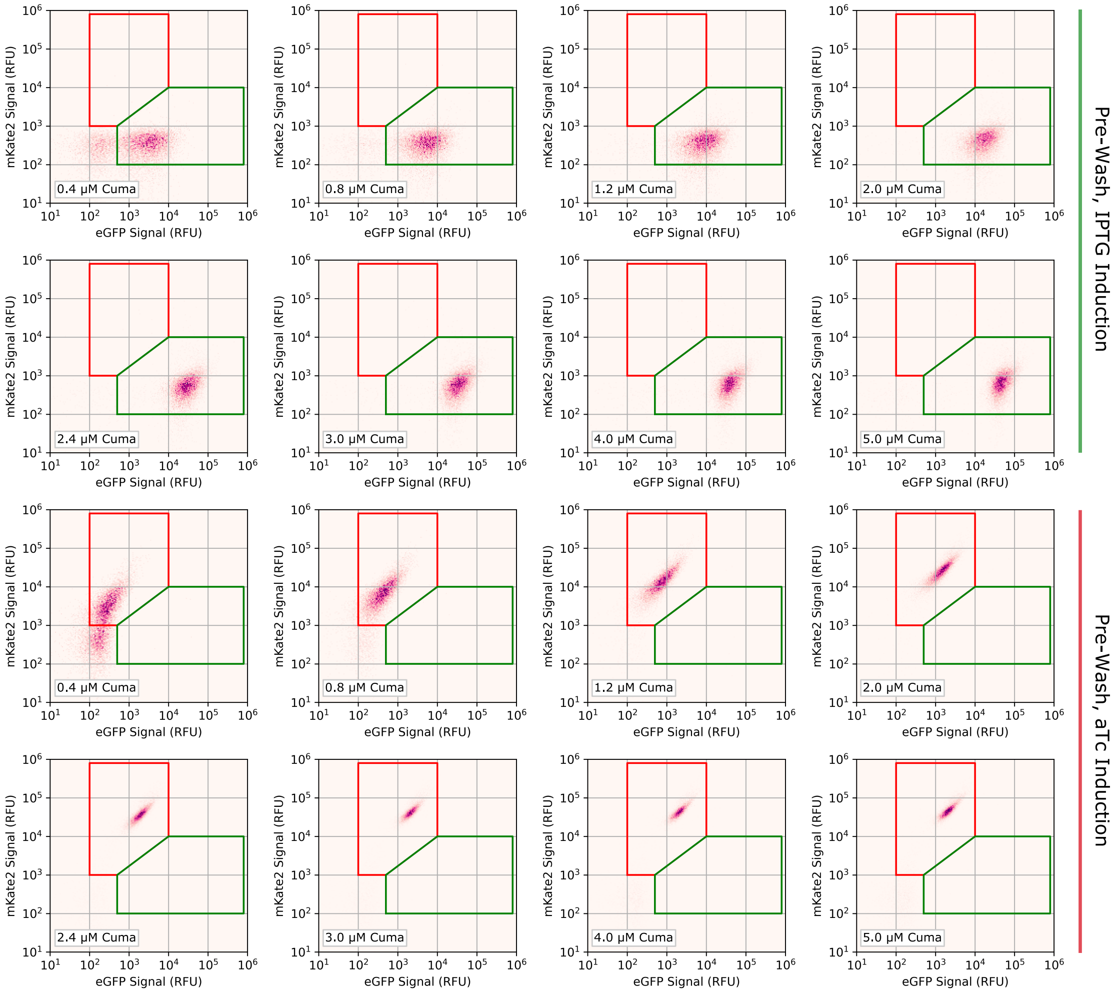

Supplementary Fig. 16. **Scatter plots depicting eGFP and mKate2 expression at the pre-wash time point when the toggle switch is harbored in TULIP considering DH10B cells with chromosomal LacI expression.** Green and red polygons indicate the regions referred to as "green state" (high TetR expression, low LacI expression after IPTG induction) and "red state" (high LacI expression, low TetR expression after aTc induction), respectively. See Figure 7 for more details.

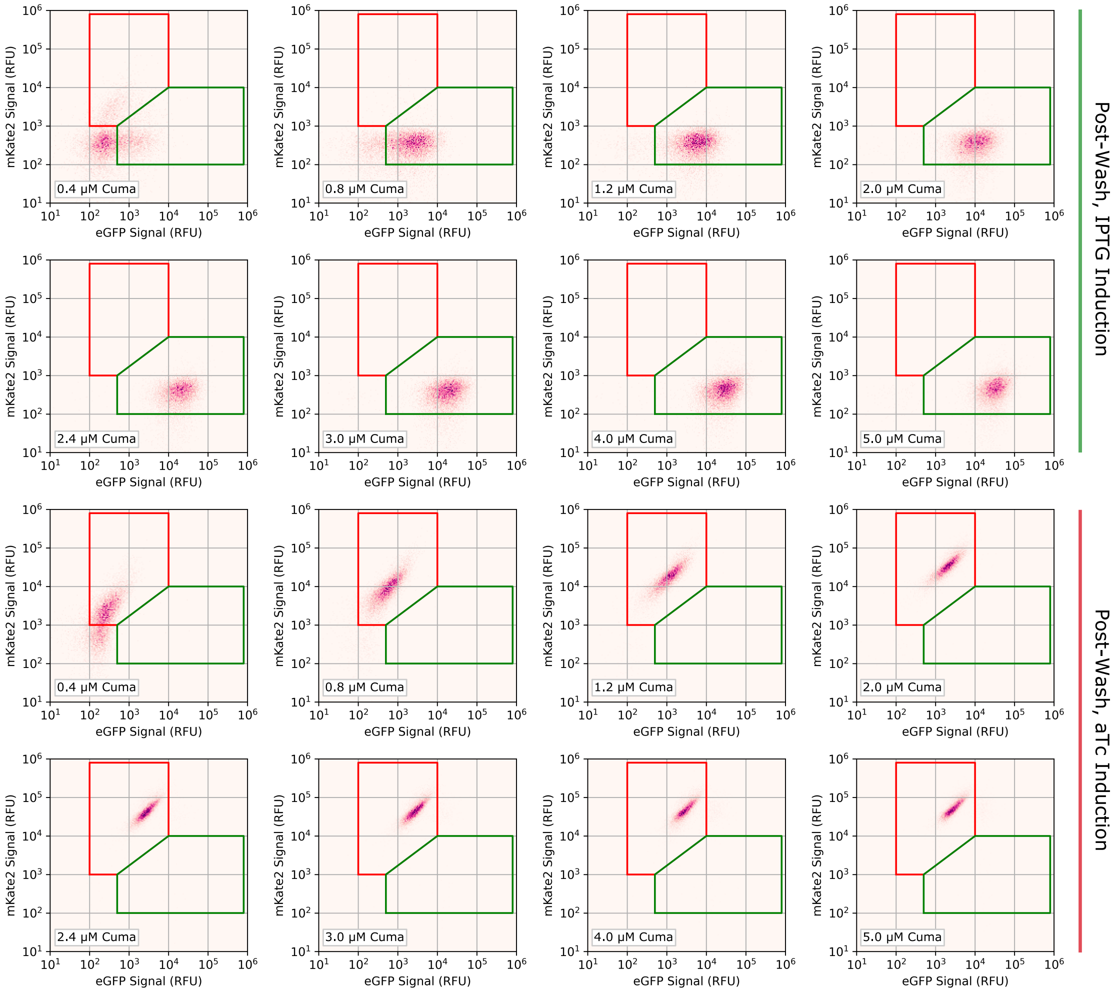

Supplementary Fig. 17. **Scatter plots depicting eGFP and mKate2 expression at the post-wash time point when the toggle switch is harbored in TULIP considering DH10B cells with chromosomal LacI expression.** Green and red polygons indicate the regions referred to as "green state" (high TetR expression, low LacI expression after IPTG induction) and "red state" (high LacI expression, low TetR expression after aTc induction), respectively. See Figure 7 for more details.

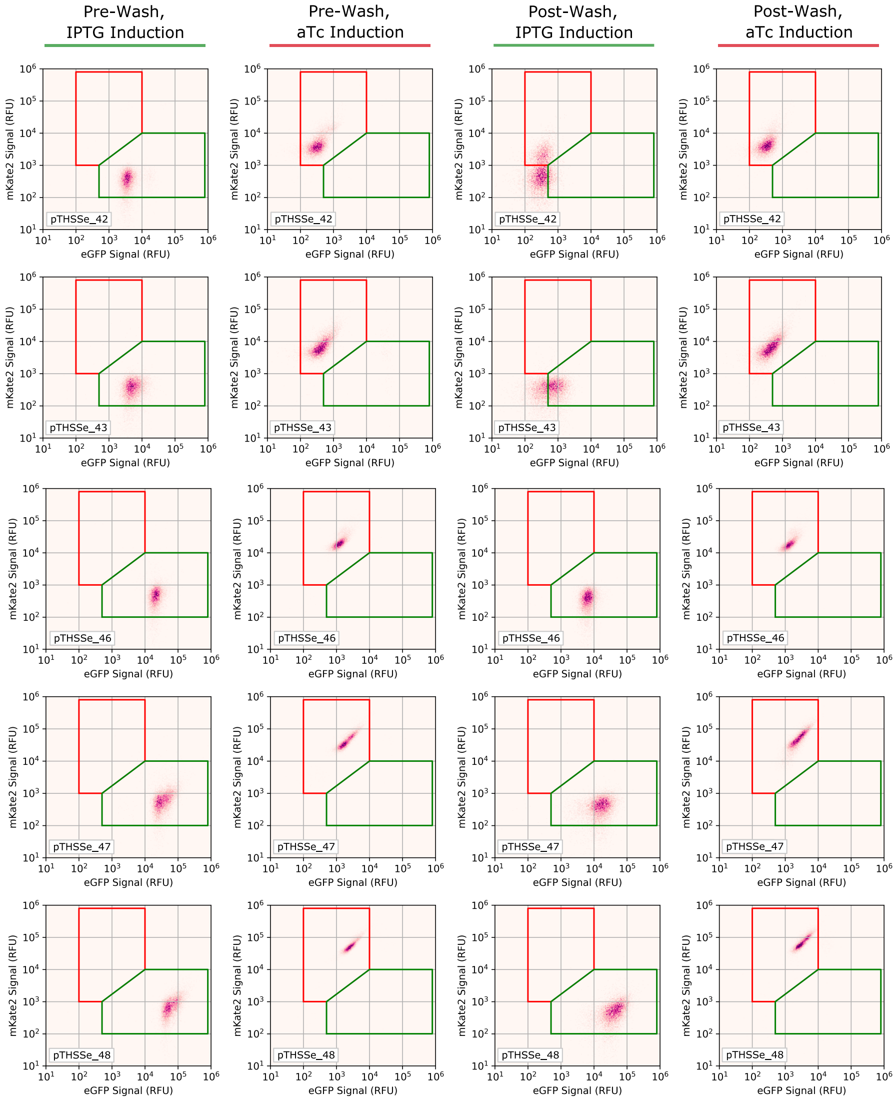

Supplementary Fig. 18. **Scatter plots depicting eGFP and mKate2 expression at the pre-wash and post-wash time points when the toggle switch is harbored in plasmids with fixed copy number considering DH10B cells with chromosomal LacI expression.** Green and red polygons indicate the regions referred to as "green state" (high TetR expression, low LacI expression after IPTG induction) and "red state" (high LacI expression, low TetR expression after aTc induction), respectively. See Figure 7 for more details.

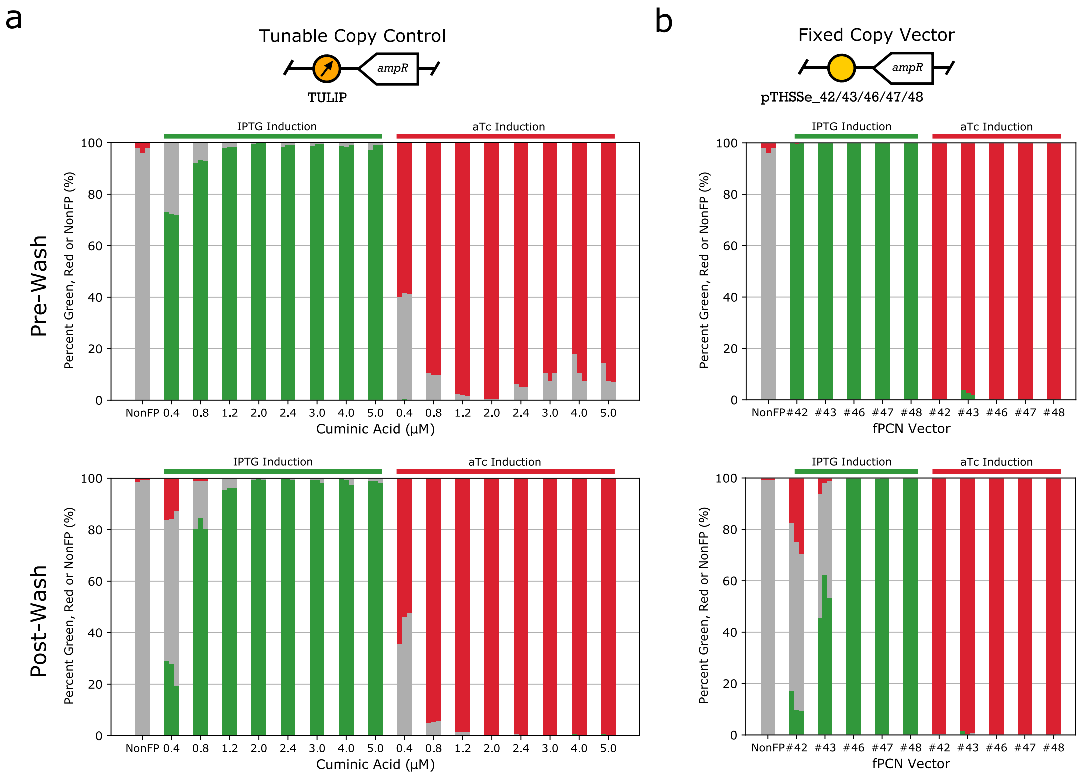

Supplementary Fig. 19. **Fraction of cells in either the TetR-dominant or the LacI-dominant states considering DH10B cells with chromosomal LacI expression.** The TetR-dominant "green state" (high eGFP and low mKate2 expression) and the LacI-dominant "red state" (low eGFP and high mKate2 expression) are defined considering the regions in Supplementary Fig. 16–Supplementary Fig. 18. Three replicates are displayed in each column, pre-wash and post-wash refer to samples taken at 8 h and at 32 h, respectively, see Figure 7 for more details.

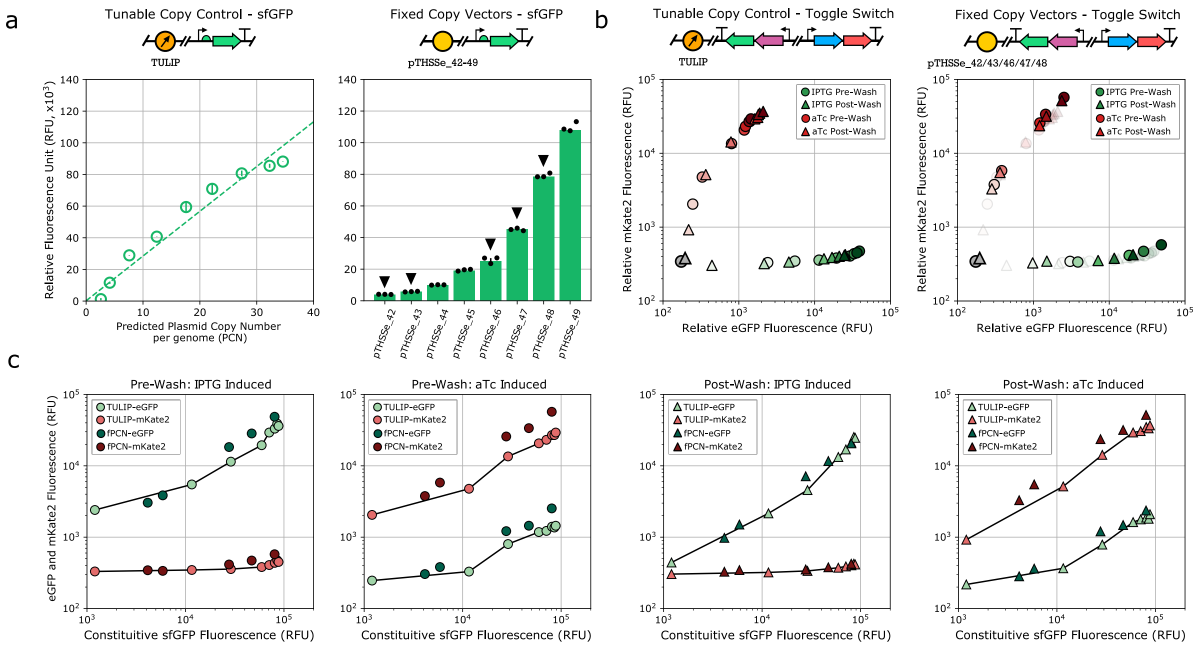

Supplementary Fig. 20. **Behavior of the toggle switch from Figure 7 in DH10B cells without chromosomal LacI expression.** LacI is co-expressed with mKate2, TetR is co-expressed with eGFP. Additional flow cytometry data and analysis are provided in Supplementary Fig. 21–Supplementary Fig. 25. Circles and triangles denote pre-wash and post-wash samples, respectively. **a** Expression of sfGFP from the empty backbone (proxy for PCN). PCN estimates for TULIP are obtained via qPCR experiments. Among plasmids in the fPCN collection, those indicated with black arrowheads were selected to harbor the toggle switch. **b** Behavior of the toggle switch when harbored in TULIP (PCN is modulated via the addition of Cuminic acid at the following concentrations: 0.4, 0.8, 1.2, 2.0, 2.4, 3.0, 4.0, and 5.0 μM) and when harbored in plasmids with fixed copy number (pTHSSe_42, pTHSSe_43, pTHSSe_46, pTHSSe_47, pTHSSe_48). Green/red indicates induction with IPTG/aTc. Darker shades indicate higher PCN, gray corresponds to empty cells with no fluorescent constructs, symbols in the background denote data obtained with TULIP. **c** Pre-wash and post-wash eGFP and mKate2 expression levels as a function of sfGFP expression from the empty backbone (proxy for PCN), both in case of TULIP (light symbols) and plasmids with fixed copy number (dark symbols).

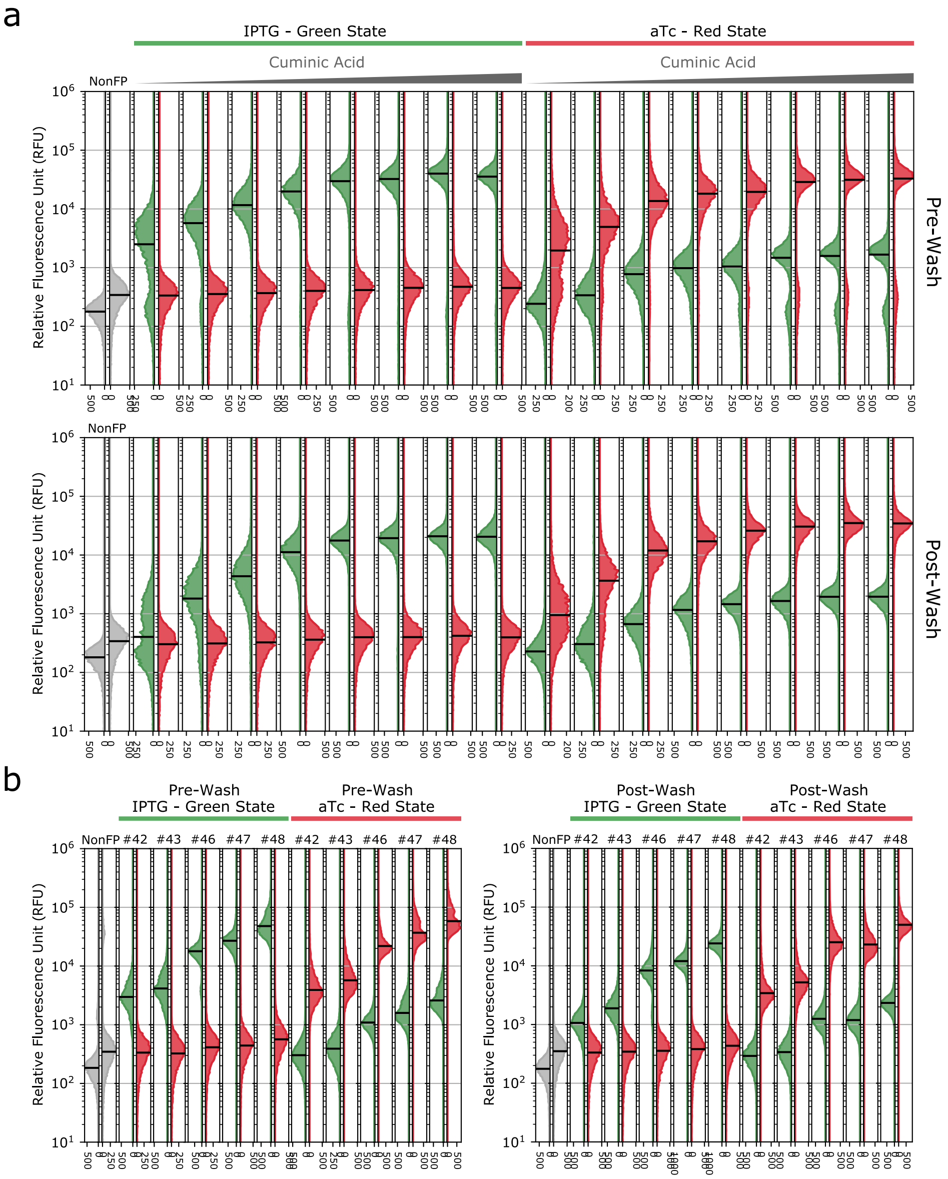

Supplementary Fig. 21. **Representative histograms for the toggle switch obtained in the experiments corresponding to Supplementary Figure 20 considering DH10B cells without chromosomal LacI expression.** Pre-wash and post-wash refer to samples taken at 8 h and at 32 h, respectively, see Figure 7a for more details. Each diverging pair of x-axis depicts data from a single sample, the left side axis represents fluorescence signal distribution in the eGFP channel, the right side axis presents signal distribution in the mKate2 channel. **a** Toggle switch is harbored in TULIP, PCN is modulated via the addition of Cuminic acid. **b** Toggle switch is harbored in plasmids with fixed PCN.

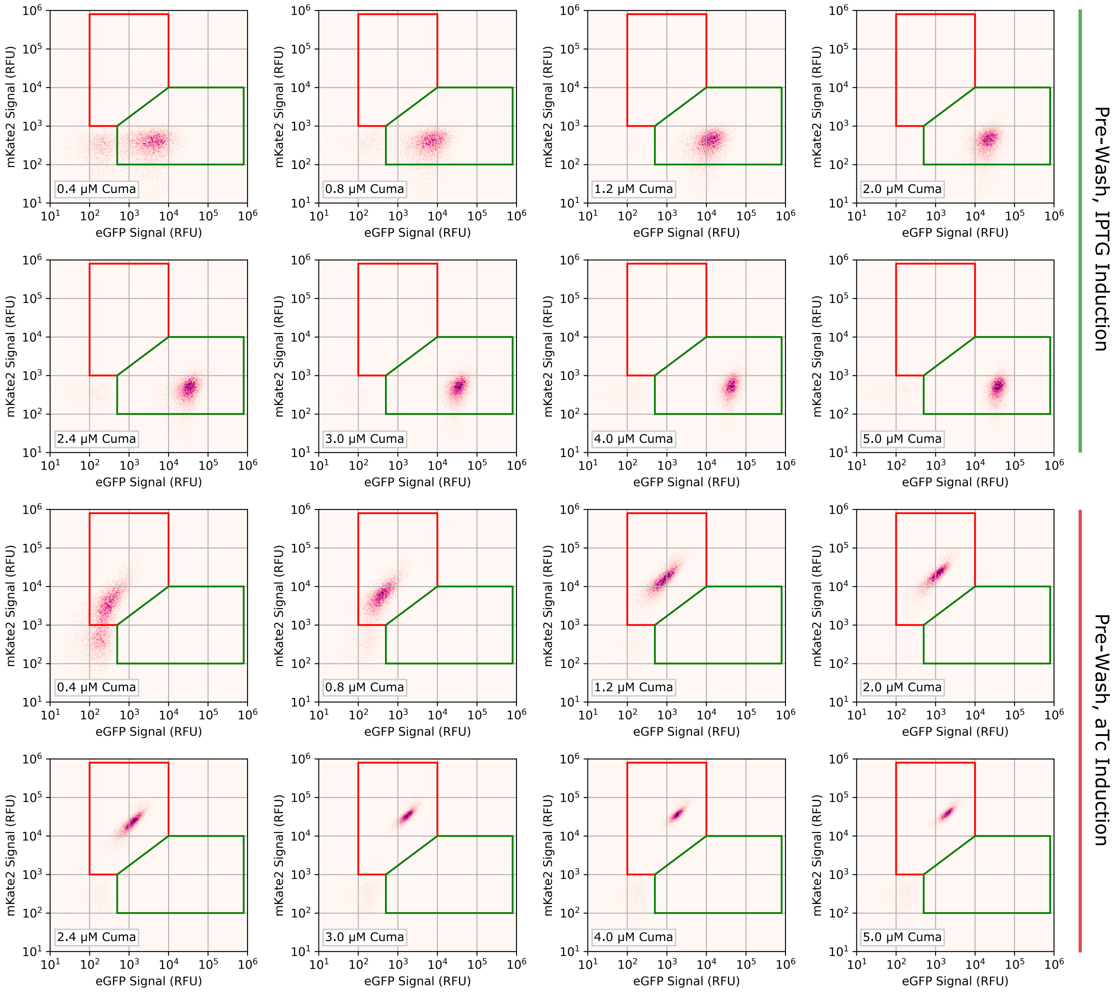

Supplementary Fig. 22. **Scatter plots depicting eGFP and mKate2 expression at the pre-wash time point when the toggle switch is harbored in TULIP considering DH10B cells without chromosomal LacI expression.** Green and red polygons indicate the regions referred to as "green state" (high TetR expression, low LacI expression after IPTG induction) and "red state" (high LacI expression, low TetR expression after aTc induction), respectively. See Figure 7 for more details.

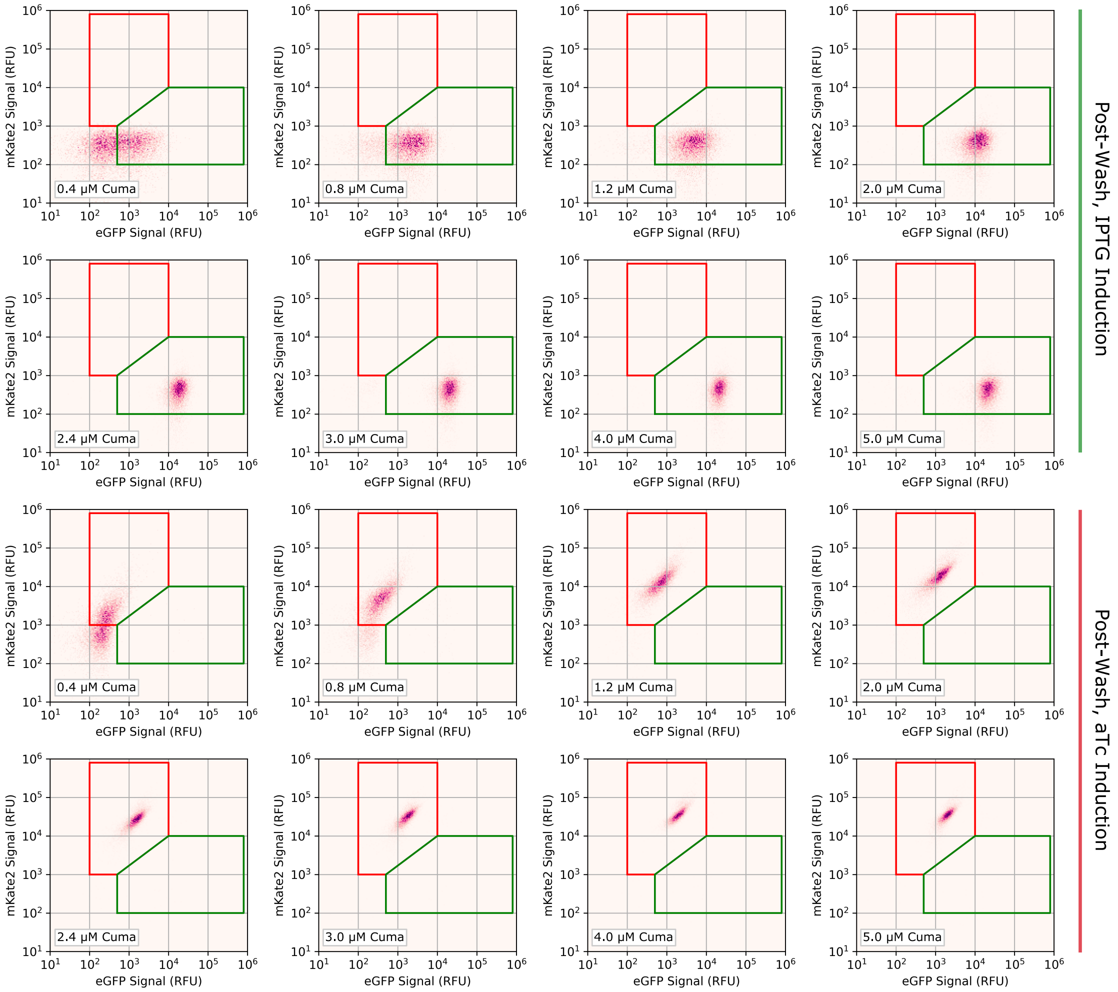

Supplementary Fig. 23. **Scatter plots depicting eGFP and mKate2 expression at the post-wash time point when the toggle switch is harbored in TULIP considering DH10B cells without chromosomal LacI expression.** Green and red polygons indicate the regions referred to as "green state" (high TetR expression, low LacI expression after IPTG induction) and "red state" (high LacI expression, low TetR expression after aTc induction), respectively. See Figure 7 for more details.

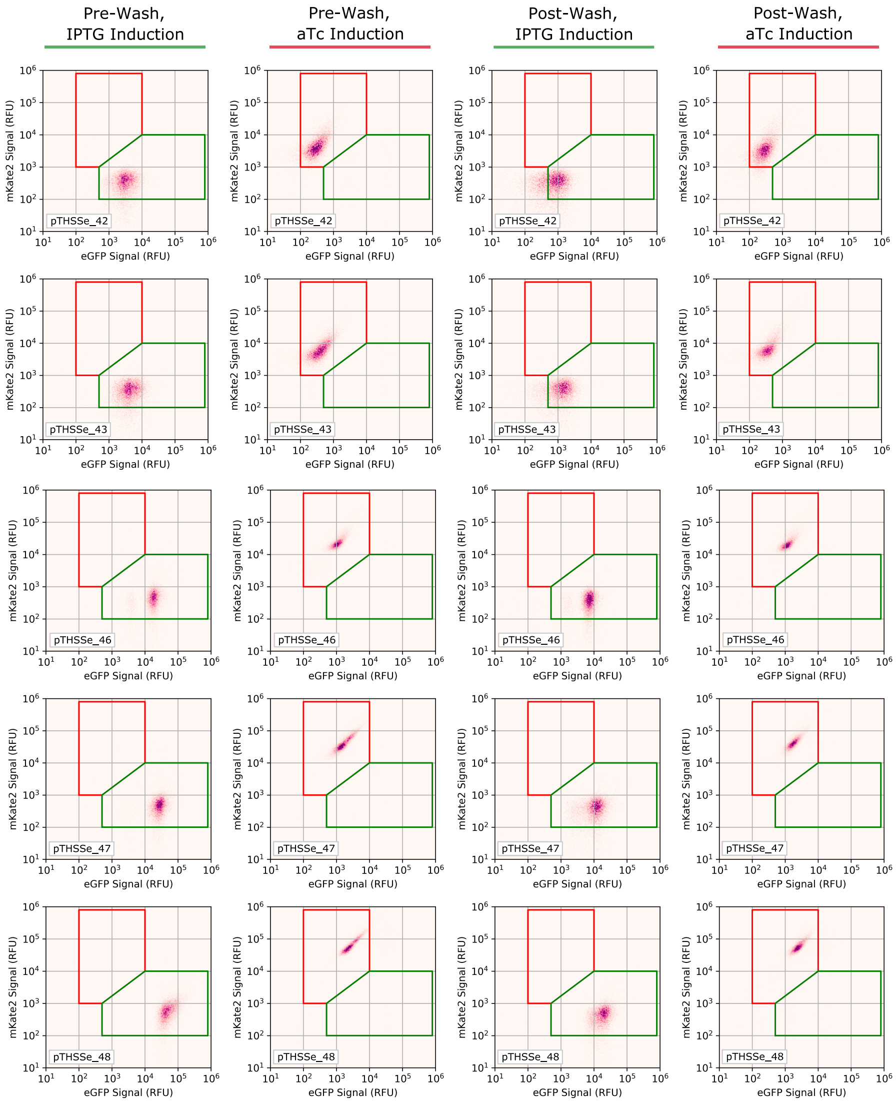

Supplementary Fig. 24. **Scatter plots depicting eGFP and mKate2 expression at the pre-wash and post-wash time points when the toggle switch is harbored in plasmids with fixed copy number considering DH10B cells without chromosomal LacI expression.** Green and red polygons indicate the regions referred to as "green state" (high TetR expression, low LacI expression after IPTG induction) and "red state" (high LacI expression, low TetR expression after aTc induction), respectively. See Figure 7 for more details.

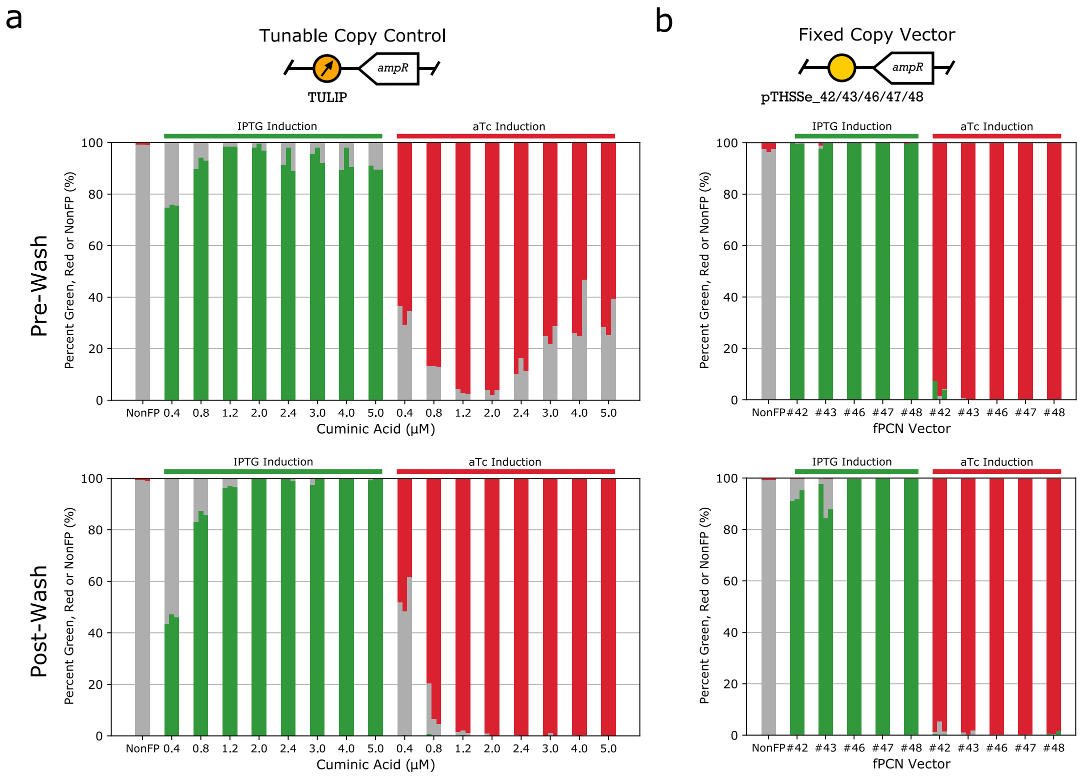

Supplementary Fig. 25. **Fraction of cells in either the TetR-dominant or the LacI-dominant states considering DH10B cells without chromosomal LacI expression.** The TetR-dominant "green state" (high eGFP and low mKate2 expression) and the LacI-dominant "red state" (low eGFP and high mKate2 expression) are defined considering the regions in Supplementary Fig. 22–Supplementary Fig. 24. Three replicates are displayed in each column, pre-wash and post-wash refer to samples taken at 8 h and at 32 h, respectively, see Figure 7 for more details.

1.8 TULIP enables versatile PCN control using a variety of input stimuli

The FR-E01 strain harboring aTc-inducible dCas9 in its genome (Figure 8a) was subjected to a range of inducer concentrations to characterize dCas9 protein expression toxicity. According to the data presented in Supplementary Fig. 26a, aTc concentrations exceeding 1 μM perturbed cell growth significantly. Therefore, we decided to use this recommended concentration (*9*).

Based on the data in Figure 8a, while gRNA #5 offered the most promising initial response to Cuminic acid, gRNA #6 displayed similar performance. Therefore, we replicated the experiments underpinning the results presented in Figure 8bc, but this time using gRNA #6 to target the cymRAM gene. Similarly to our top pick (gRNA #5) featured in Figure 8bc, gRNA #6 also ensures tunable PCN control using the alternative inputs AHL (Supplementary Fig. 26b) and Van (Supplementary Fig. 26c), further illustrating the flexibility of the CRISPRi-based regulatory architecture outlined in Figure 8a.

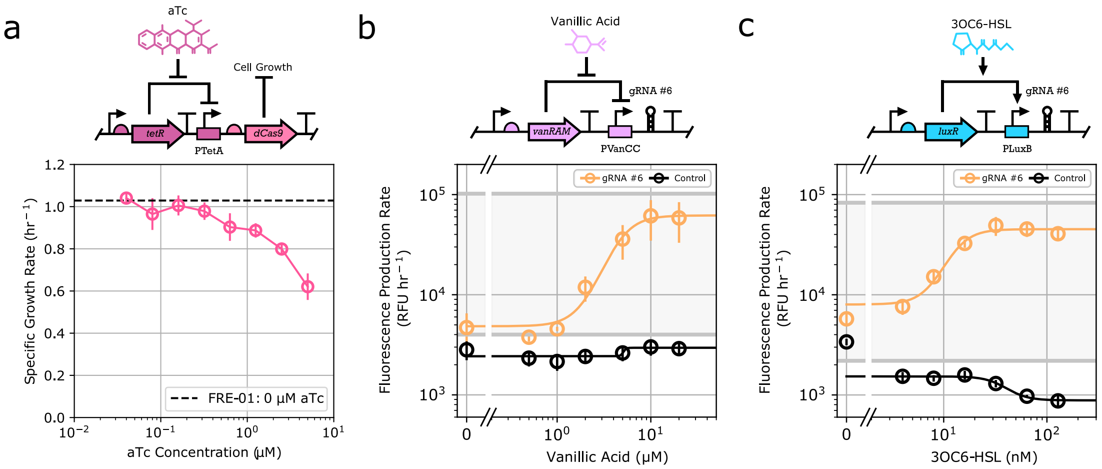

Supplementary Fig. 26. **Toxicity of dCas9 and performance of the CRISPRi-based PCN control using gRNA #6.** The gray shaded region indicates TULIP's functional range corresponding to 0.4–5.0 μM Cuminic acid induction. Control refers to constructs using gRNA #9. **a** Growth rate effect of aTc-inducible dCas9 expression using the FR-E01 strain (*9*). Dashed line corresponds to growth rate in the absence of aTc induction. **b** Tunable PCN control via Van induction using gRNA #6. **c** Tunable PCN control via AHL induction using gRNA #6.

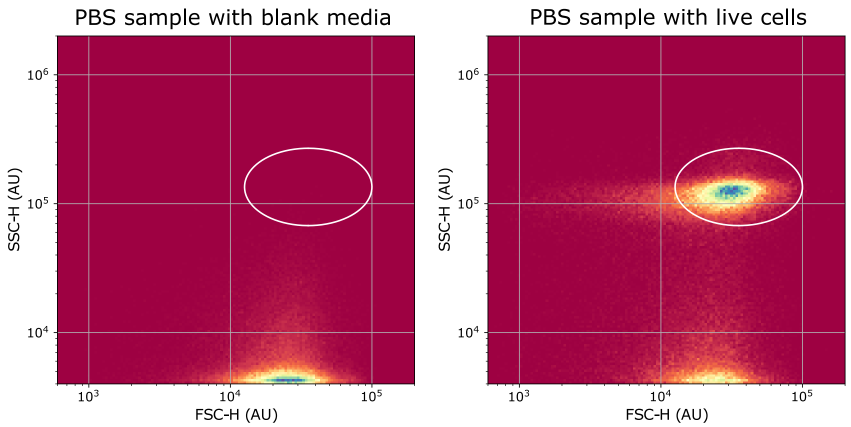

Supplementary Fig. 27. **Example of gating in flow cytometry experiments.**

1.9 Gating cells in flow cytometry experiments

For analyzing samples using the flow cytometer, at the sampling timepoint cells growing in liquid culture were diluted into PBS solution and were analyzed immediately by the flow cytometer. The FACSAria III Sorter was used for library screening, and the Attune NxT was used for routine sample analysis. Forward Scatter Height (FSC-H) and Side Scatter Height (SSC-H) were plotted on a logarithmic scale to visualize and gate the events. PBS samples with uninoculated M9-Gluc media were ran frequently in each experiment to verify the absence of cells in PBS and in blank media samples. The positive control of PBS with live cells was used to indicate where to place the gate for counting events, demarcated by the white ellipse in Supplementary Fig. 27.

1.10 Kinetic microplate reader data

In Supplementary Fig. 28 we replicate the analysis presented in Figure 3d to demonstrate that TULIP's response to Cuminic acid in all tested strains is qualitatively similar considering sfGFP production rate at its peak or its value when growth rate is maximized using kinetic microplate reader data. Similarly, data in Supplementary Fig. 29 illustrate that metabolic burden due to

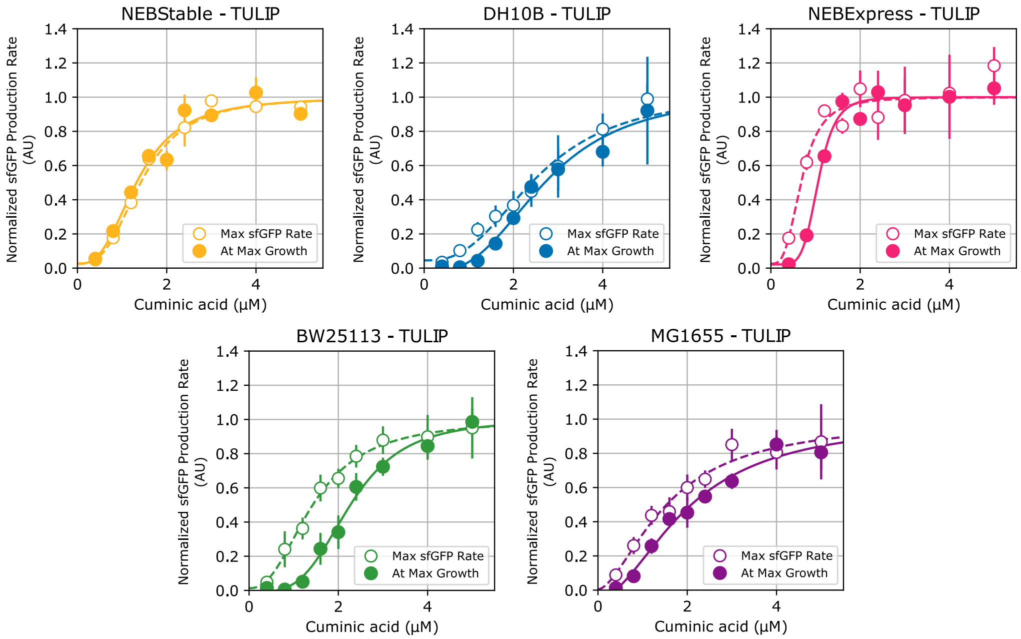

Supplementary Fig. 28. **Response of TULIP to Cuminic acid in NEBStable (yellow), DH10B (blue), NEBExpress (magenta), BW25113 (green), and MG1655 (purple).** Empty circles and dashed lines indicate peak sfGFP production rate, full circles and solid lines correspond to sfGFP production rate when growth rate is maximized. Values are normalized by the maximum of the fitted curves (4 parameter logistic curve). Each experiment was conducted in biological triplicates ($n = 3$), data points and error bars represent the mean and standard deviation across replicates.

mScarI expression has a similar impact on sfGFP production considering the sensor modules in Figure 5 at different timepoints. These results illustrate that our findings hold across a variety of timepoints.

Following this, we include the specific growth rate data obtained in kinetic microplate reader experiments in Supplementary Fig. 30–Supplementary Fig. 36 for Figures 5–6, 8, Supplementary Fig. 13, Supplementary Fig. 14, and Supplementary Fig. 26 (data for Figures 2–3, Supplementary Fig. 1, Supplementary Fig. 3, and Supplementary Fig. 7 are included in the figures themselves), then the detailed timecourses in Supplementary Fig. 37–Supplementary Fig. 101; in these, the data have been averaged across each respective triplicate group over the timecourse

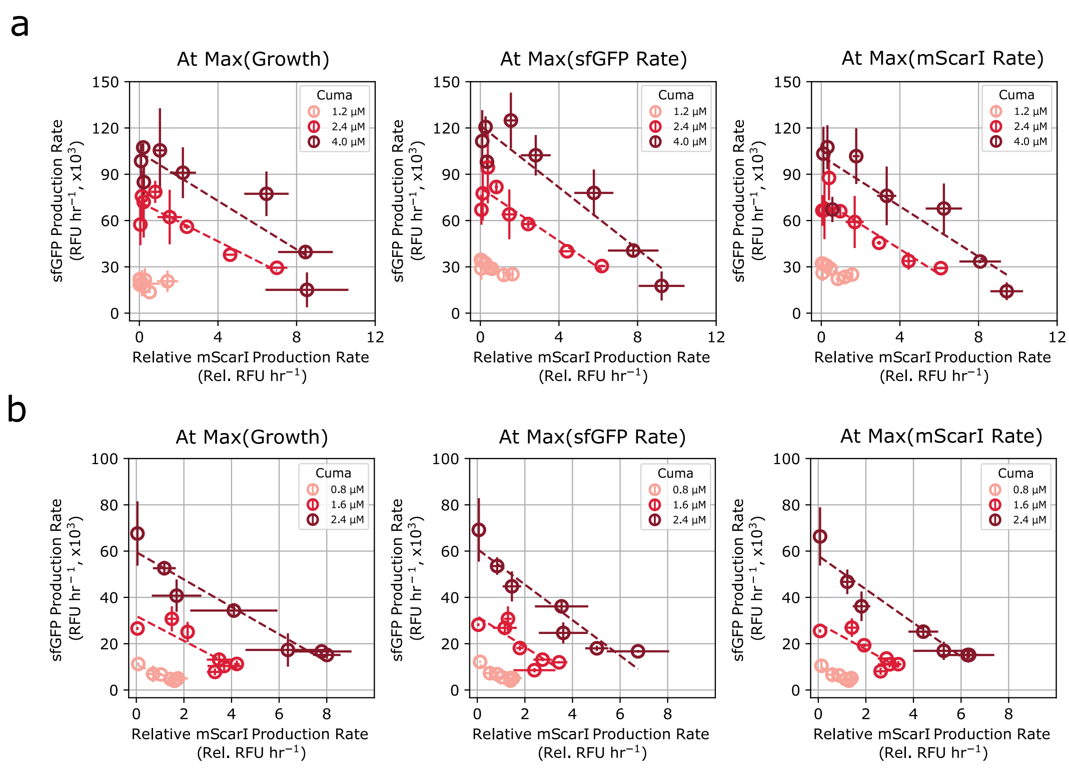

Supplementary Fig. 29. **Impact of mScarI expression on sfGFP production in case of the sensor modules in Figure 5.** Subsequent panels correspond to the timepoints when growth rate, sfGFP production rate, and mScarI production rate is maximized, respectively. Each experiment was conducted in biological triplicates ($n = 3$), data points and error bars represent the mean and standard deviation across replicates. **a** Data correspond to the sensor module in Figure 5a. **b** Data correspond to the sensor module in Figure 5b.

of the experiment, and shaded regions of the same color represent standard deviation.

Data in Figures 2–3 highlight that the empty TULIP backbone does not significantly alter growth rate in any of the strains, and TULIP's impact is similarly negligible even in the presence of the additional CRISPRi-based layer of control based on data in Supplementary Fig. 33–Supplementary Fig. 34. The results in Supplementary Fig. 30–Supplementary Fig. 32 and Supplementary Fig. 35 further suggest that the modules from Figure 5 can be deployed in TULIP without considerably affecting the host (DH10B or BW25113), in spite of the substantial impact on sfGFP expression due to resource competition (Figure 5).

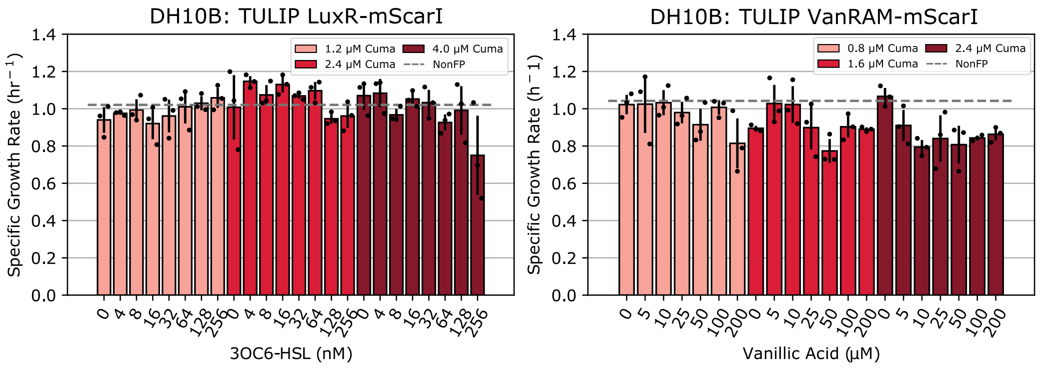

Supplementary Fig. 30. **Growth rates corresponding to the experiments presented in Figure 5.** Gray dashed line indicates the growth rate of NonFP cells. Each experiment was conducted in biological triplicates ($n = 3$), data points and error bars represent the mean and standard deviation across replicates.

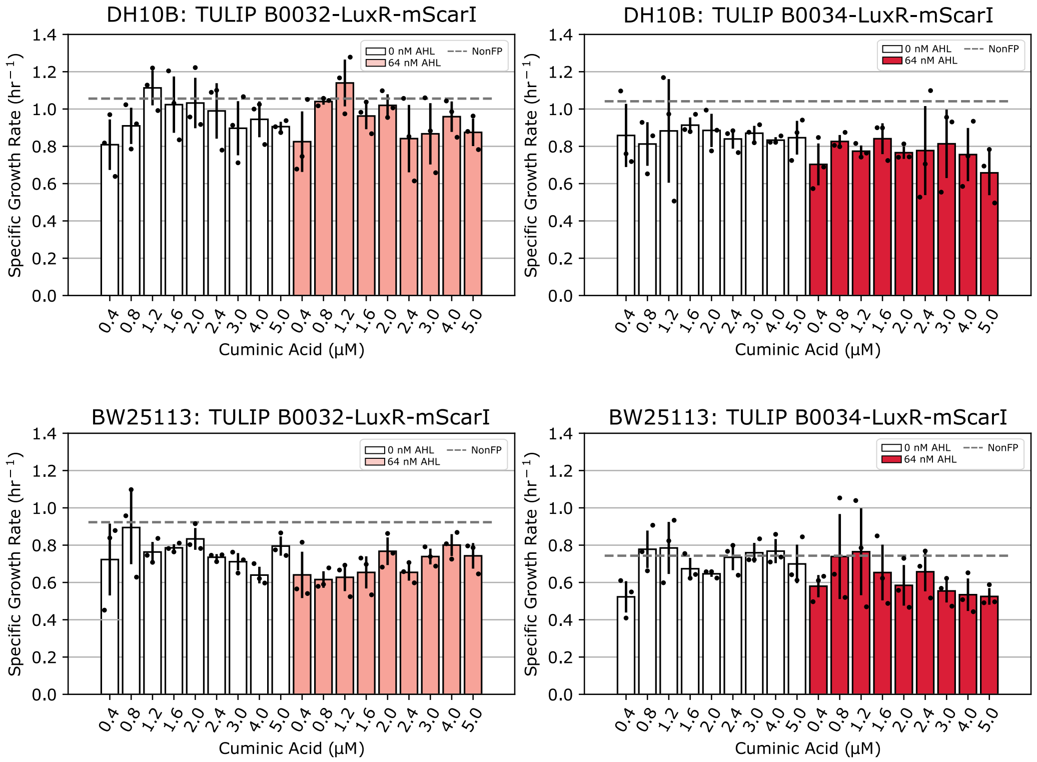

Supplementary Fig. 31. **Growth rates corresponding to the experiments presented in Figure 6a.** Gray dashed line indicates the growth rate of NonFP cells. Each experiment was conducted in biological triplicates ($n = 3$), data points and error bars represent the mean and standard deviation across replicates.

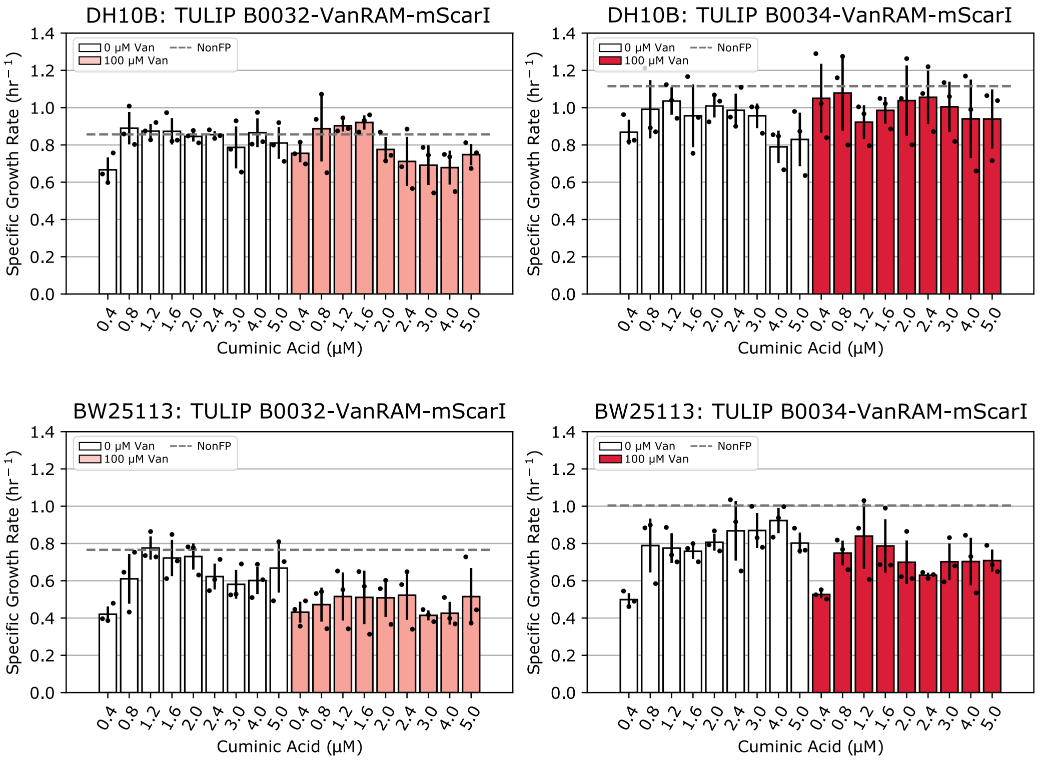

Supplementary Fig. 32. **Growth rates corresponding to the experiments presented in Figure 6b.** Gray dashed line indicates the growth rate of NonFP cells. Each experiment was conducted in biological triplicates ($n = 3$), data points and error bars represent the mean and standard deviation across replicates.

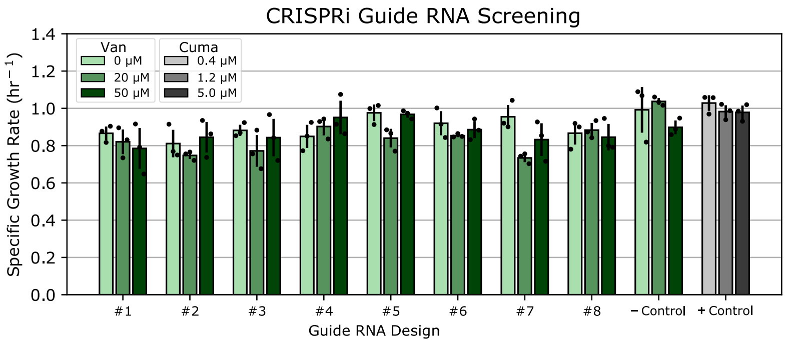

Supplementary Fig. 33. **Growth rates corresponding to the experiments presented in Figure 8.** Each experiment was conducted in biological triplicates ($n = 3$), data points and error bars represent the mean and standard deviation across replicates.

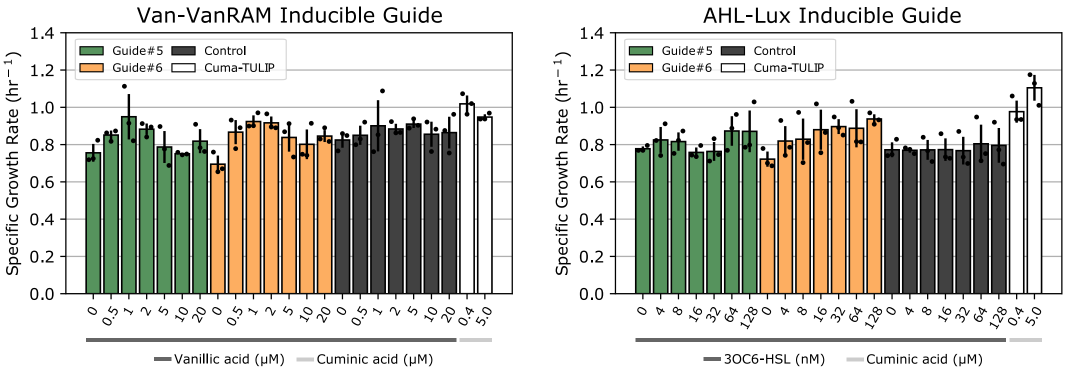

Supplementary Fig. 34. **Growth rates corresponding to the experiments presented in Figure 8 and Supplementary Fig. 26.** Control refers to constructs using gRNA #9. Values on the x-axis refer to induction with Vanillic acid or AHL in case of green (gRNA #5), orange (gRNA #6), and dark gray bars (gRNA #9), and to induction via Cuminic acid in case of white bars (TULIP). Each experiment was conducted in biological triplicates ($n = 3$), data points and error bars represent the mean and standard deviation across replicates.

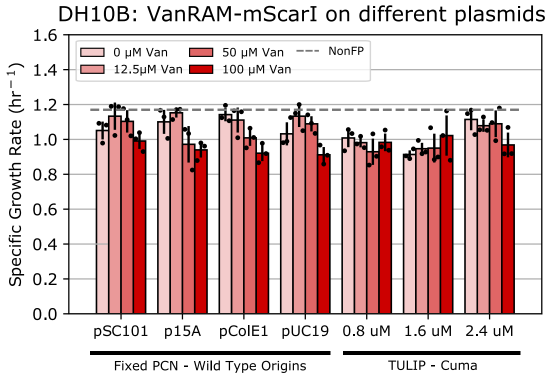

Supplementary Fig. 35. **Growth rates corresponding to the experiments presented in Supplementary Fig. 13.** Gray dashed line indicates the growth rate of NonFP cells. Each experiment was conducted in biological triplicates ($n = 3$), data points and error bars represent the mean and standard deviation across replicates.

Reduced growth rates in Supplementary Fig. 36 likely stem from AmtR toxicity (*10*), especially considering the significantly higher PCN of TULIP in BW25113 than in DH10B (Figure 3). Importantly, despite severely impacted growth, Cuma-induced PCN upregulation via TULIP still functions: e.g., although in the absence of Van induction cells grow 80% slower in BW25113 at 5.0 μM than at 0.4 μM Cuma concentration (Supplementary Fig. 36), mScarI

production is approximately 10-fold higher (Supplementary Fig. 91).

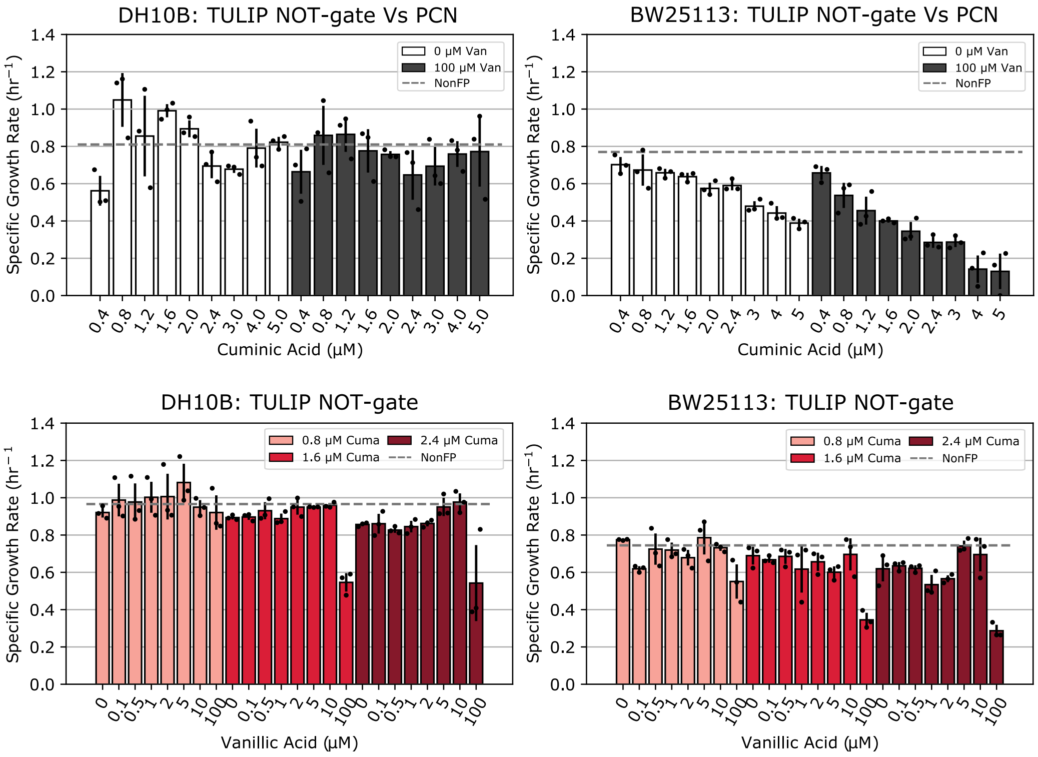

Supplementary Fig. 36. **Growth rates corresponding to the experiments presented in Supplementary Fig. 14.** Gray dashed line indicates the growth rate of NonFP cells. Each experiment was conducted in biological triplicates ($n = 3$), data points and error bars represent the mean and standard deviation across replicates.

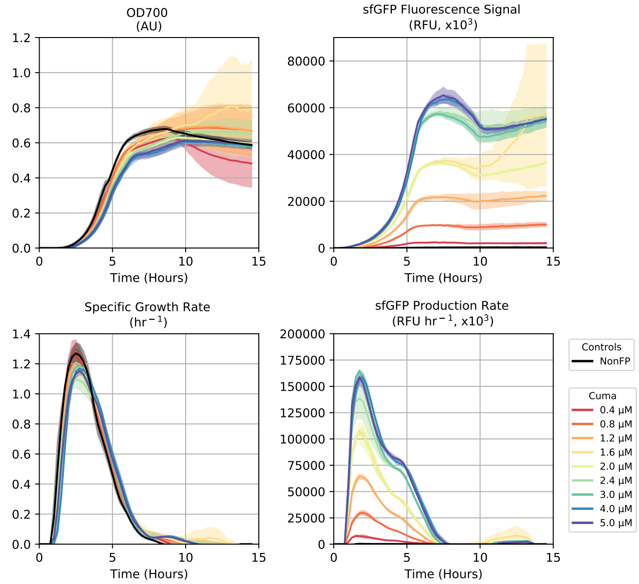

Supplementary Fig. 37. **Microplate reader data for Figure 2c.** Each experiment was conducted in biological triplicates ($n = 3$), data points and error bars represent the mean and standard deviation across replicates.

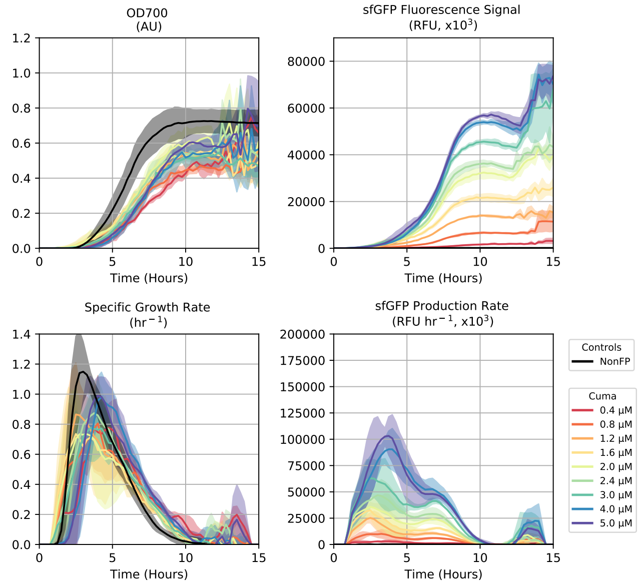

Supplementary Fig. 38. **Microplate reader data for Figure 3d corresponding to the experiments carried out in DH10B.** Each experiment was conducted in biological triplicates ($n = 3$), data points and error bars represent the mean and standard deviation across replicates.

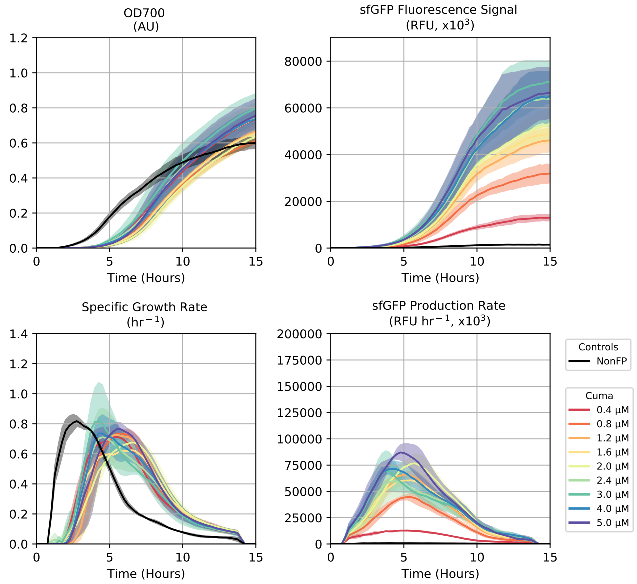

Supplementary Fig. 39. **Microplate reader data for Figure 3d corresponding to the experiments carried out in NEBExpress.** Each experiment was conducted in biological triplicates ($n = 3$), data points and error bars represent the mean and standard deviation across replicates.

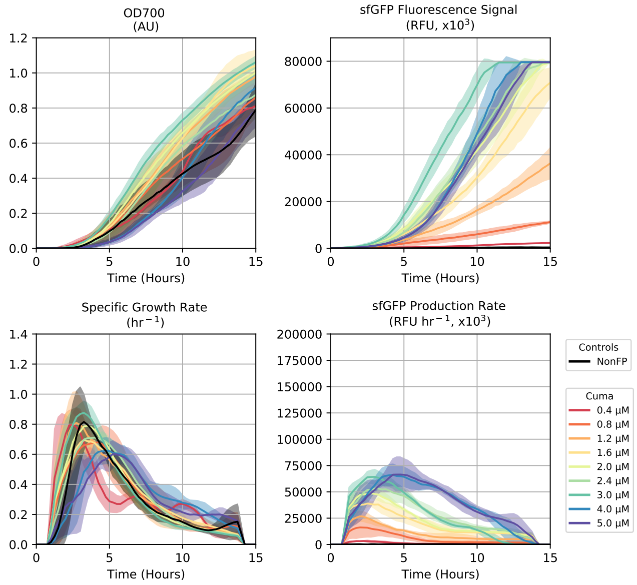

Supplementary Fig. 40. **Microplate reader data for Figure 3d corresponding to the experiments carried out in BW25113.** Each experiment was conducted in biological triplicates ($n = 3$), data points and error bars represent the mean and standard deviation across replicates.

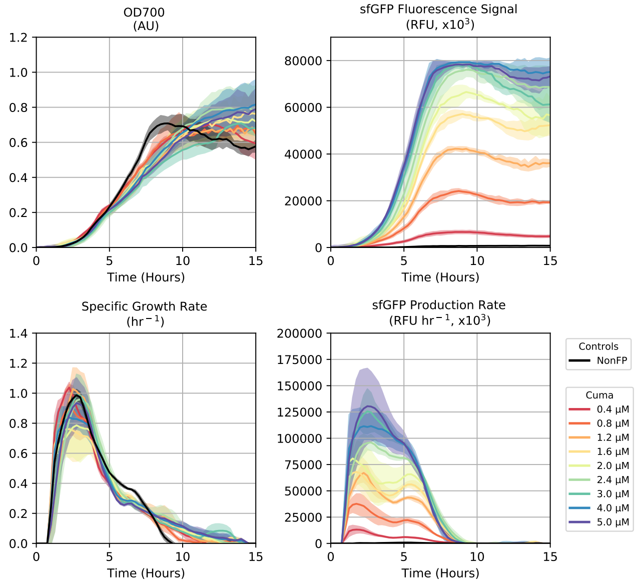

Supplementary Fig. 41. **Microplate reader data for Figure 3d corresponding to the experiments carried out in MG1655.** Each experiment was conducted in biological triplicates ($n = 3$), data points and error bars represent the mean and standard deviation across replicates.

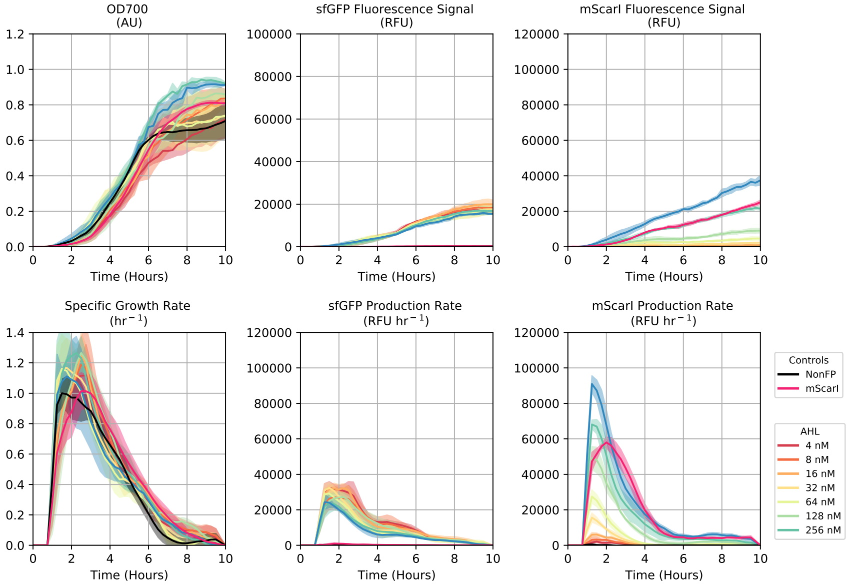

Supplementary Fig. 42. **Microplate reader data for Figure 5a corresponding to the experiments carried out in the presence of 1.2 μM Cuminic acid.** Each experiment was conducted in biological triplicates ($n = 3$), data points and error bars represent the mean and standard deviation across replicates.

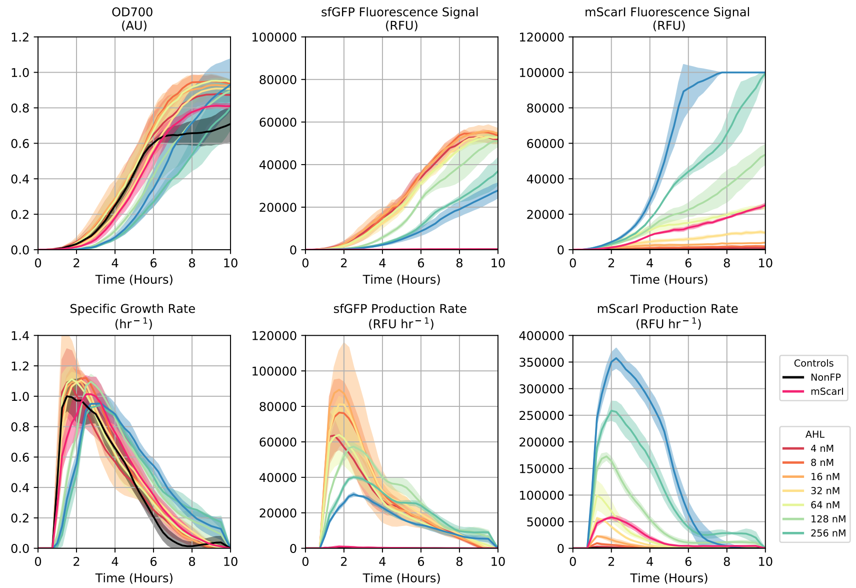

Supplementary Fig. 43. **Microplate reader data for Figure 5a corresponding to the experiments carried out in the presence of 2.4 μM Cuminic acid.** Each experiment was conducted in biological triplicates ($n = 3$), data points and error bars represent the mean and standard deviation across replicates.

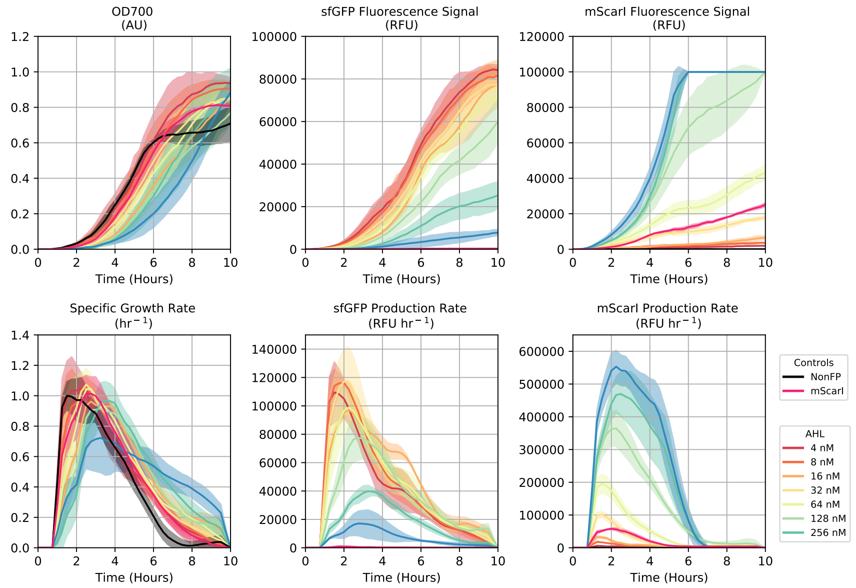

Supplementary Fig. 44. **Microplate reader data for Figure 5a corresponding to the experiments carried out in the presence of 4.0 μM Cuminic acid.** Each experiment was conducted in biological triplicates ($n = 3$), data points and error bars represent the mean and standard deviation across replicates.

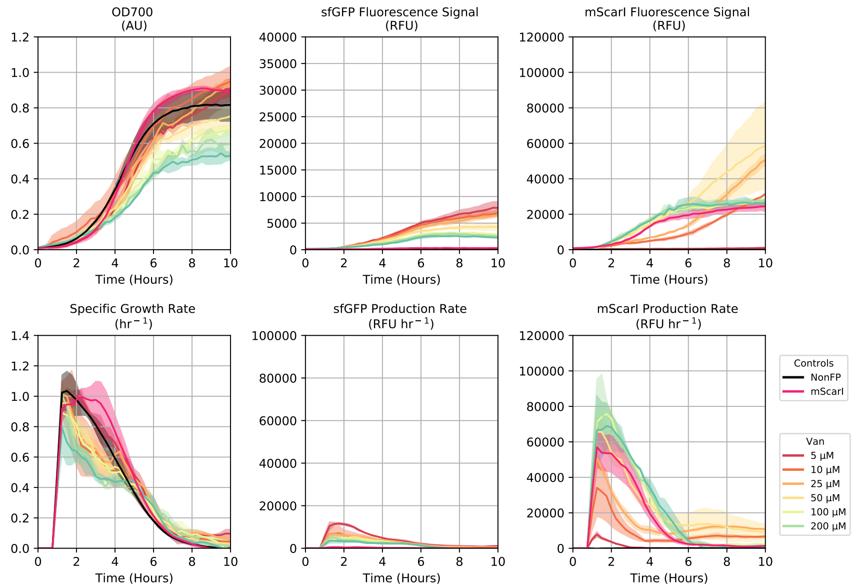

Supplementary Fig. 45. **Microplate reader data for Figure 5b corresponding to the experiments carried out in the presence of 0.8 μM Cuminic acid.** Each experiment was conducted in biological triplicates ($n = 3$), data points and error bars represent the mean and standard deviation across replicates.

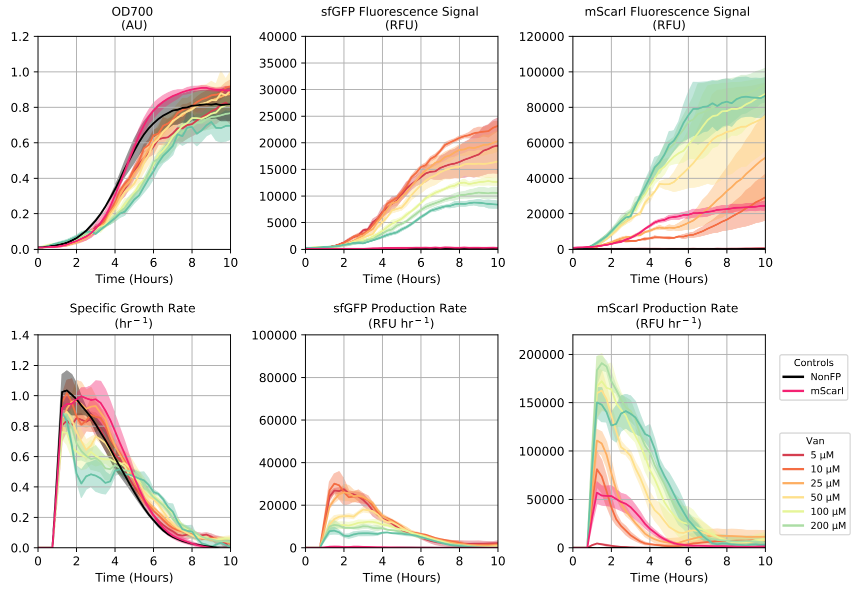

Supplementary Fig. 46. **Microplate reader data for Figure 5b corresponding to the experiments carried out in the presence of 1.6 μM Cuminic acid.** Each experiment was conducted in biological triplicates ($n = 3$), data points and error bars represent the mean and standard deviation across replicates.

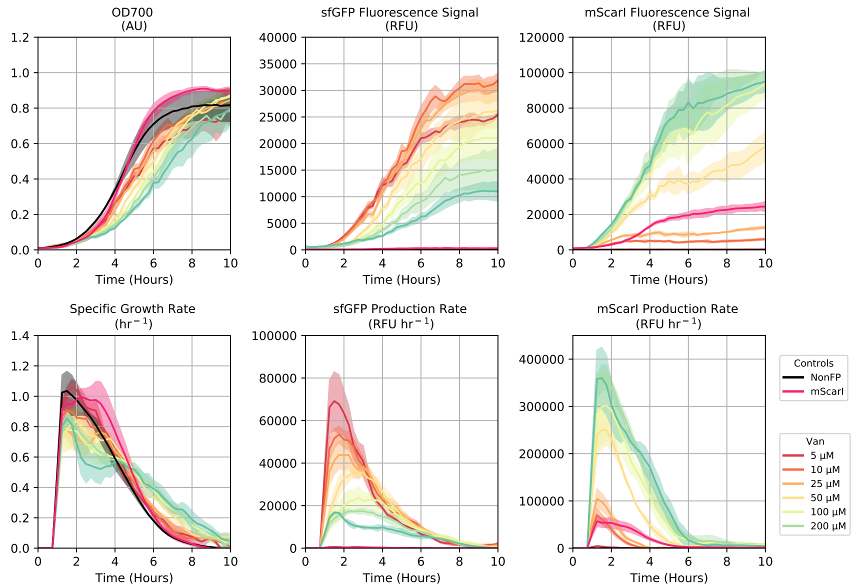

Supplementary Fig. 47. **Microplate reader data for Figure 5b corresponding to the experiments carried out in the presence of 2.4 μM Cuminic acid.** Each experiment was conducted in biological triplicates ($n = 3$), data points and error bars represent the mean and standard deviation across replicates.

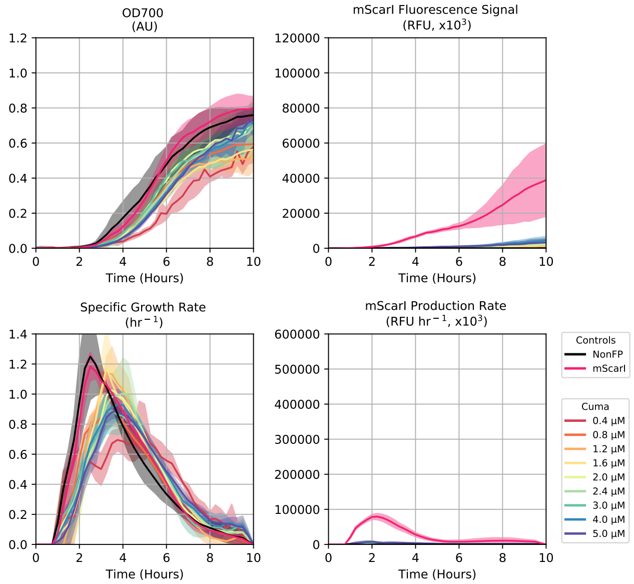

Supplementary Fig. 48. **Microplate reader data for Figure 6a corresponding to the experiments carried out in DH10B with B0032 in the presence of 0 nM AHL.** Each experiment was conducted in biological triplicates ($n = 3$), data points and error bars represent the mean and standard deviation across replicates.

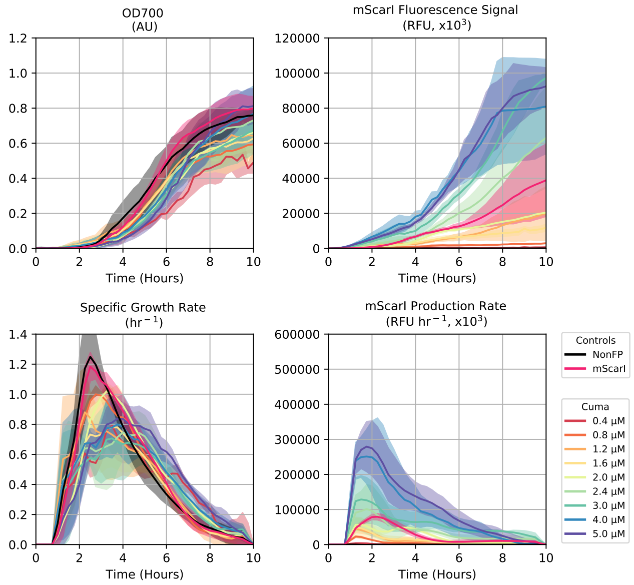

Supplementary Fig. 49. **Microplate reader data for Figure 6a corresponding to the experiments carried out in DH10B with B0032 in the presence of 64 nM AHL.** Each experiment was conducted in biological triplicates ($n = 3$), data points and error bars represent the mean and standard deviation across replicates.

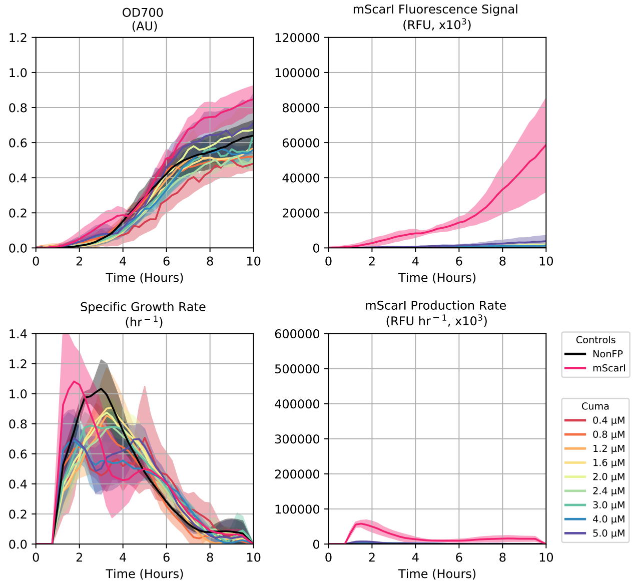

Supplementary Fig. 50. **Microplate reader data for Figure 6a corresponding to the experiments carried out in DH10B with B0034 in the presence of 0 nM AHL.** Each experiment was conducted in biological triplicates ($n = 3$), data points and error bars represent the mean and standard deviation across replicates.

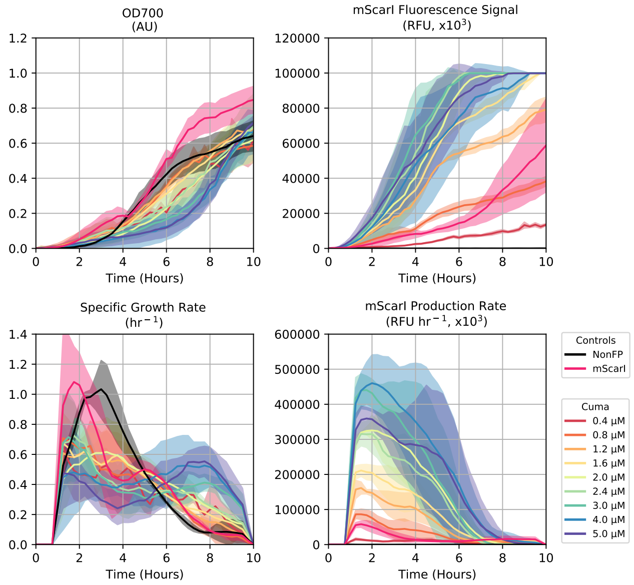

Supplementary Fig. 51. **Microplate reader data for Figure 6a corresponding to the experiments carried out in DH10B with B0034 in the presence of 64 nM AHL.** Each experiment was conducted in biological triplicates ($n = 3$), data points and error bars represent the mean and standard deviation across replicates.

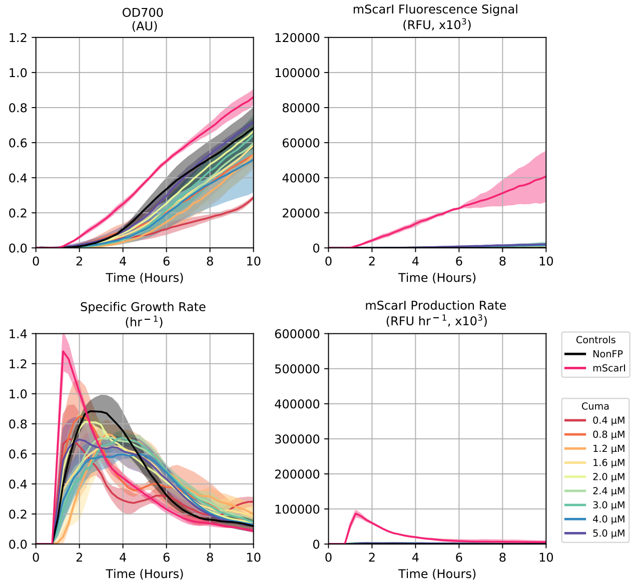

Supplementary Fig. 52. **Microplate reader data for Figure 6a corresponding to the experiments carried out in BW25113 with B0032 in the presence of 0 nM AHL.** Each experiment was conducted in biological triplicates ($n = 3$), data points and error bars represent the mean and standard deviation across replicates.

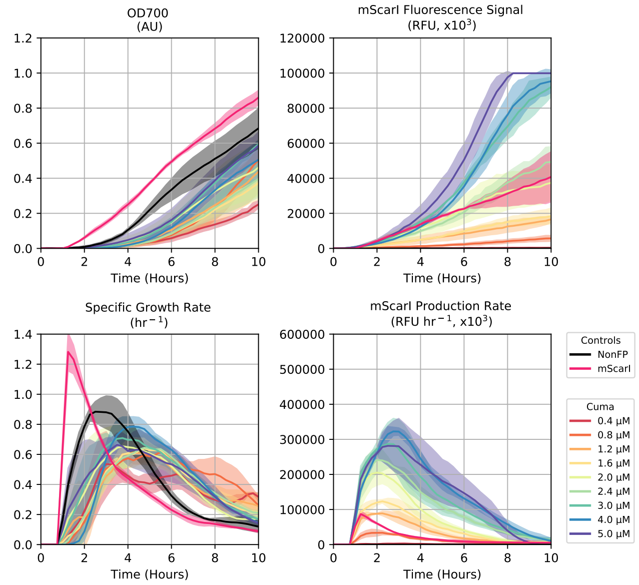

Supplementary Fig. 53. **Microplate reader data for Figure 6a corresponding to the experiments carried out in BW25113 with B0032 in the presence of 64 nM AHL.** Each experiment was conducted in biological triplicates ($n = 3$), data points and error bars represent the mean and standard deviation across replicates.

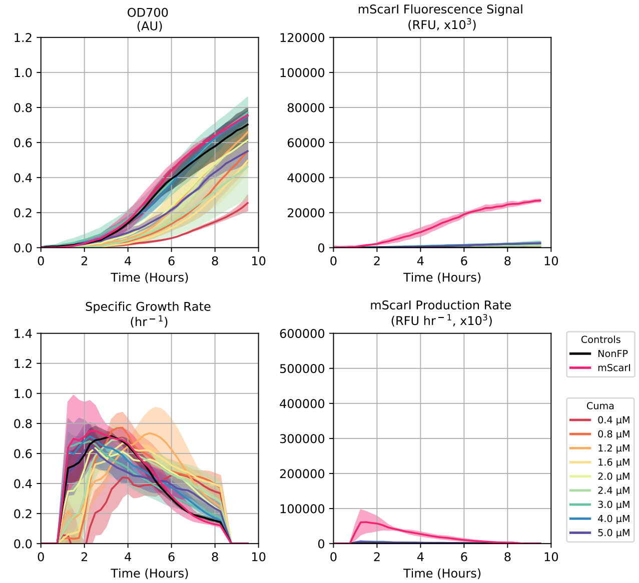

Supplementary Fig. 54. **Microplate reader data for Figure 6a corresponding to the experiments carried out in BW25113 with B0034 in the presence of 0 nM AHL.** Each experiment was conducted in biological triplicates ($n = 3$), data points and error bars represent the mean and standard deviation across replicates.

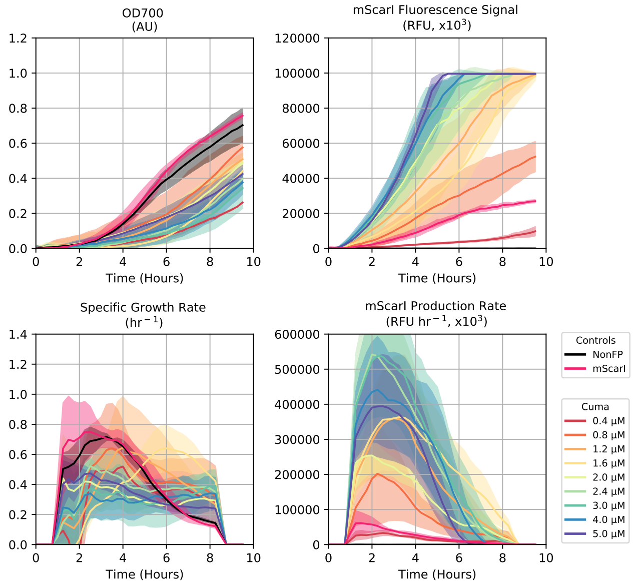

Supplementary Fig. 55. **Microplate reader data for Figure 6a corresponding to the experiments carried out in BW25113 with B0034 in the presence of 64 nM AHL.** Each experiment was conducted in biological triplicates ($n = 3$), data points and error bars represent the mean and standard deviation across replicates.

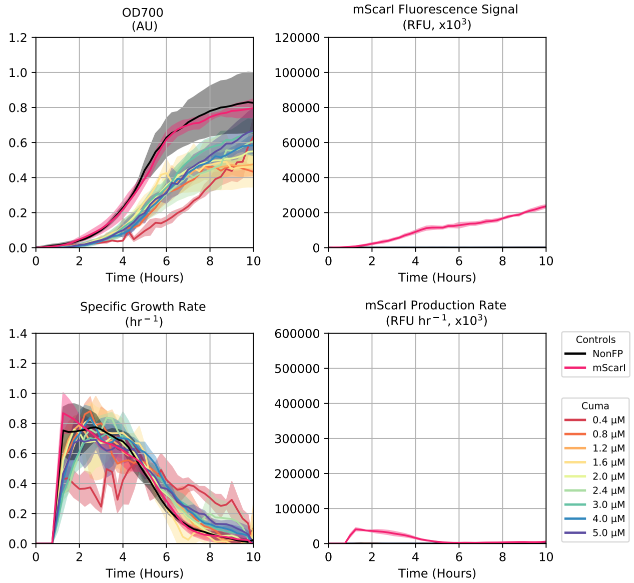

Supplementary Fig. 56. **Microplate reader data for Figure 6b corresponding to the experiments carried out in DH10B with B0032 in the presence of 0 μM Van.** Each experiment was conducted in biological triplicates ($n = 3$), data points and error bars represent the mean and standard deviation across replicates.

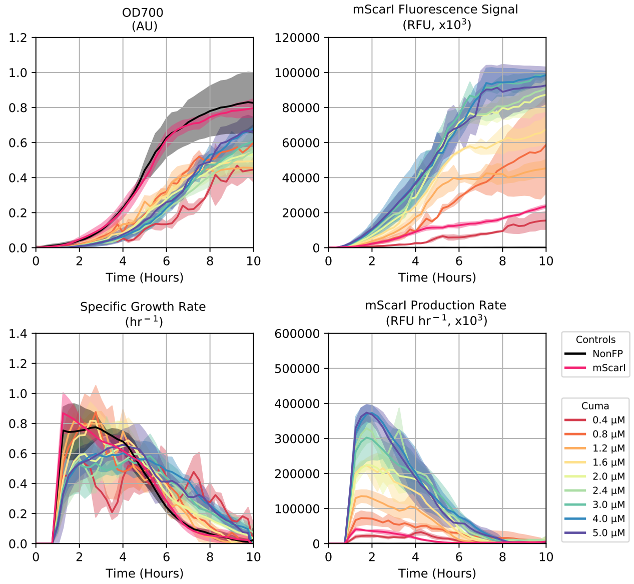

Supplementary Fig. 57. **Microplate reader data for Figure 6b corresponding to the experiments carried out in DH10B with B0032 in the presence of 100 μM Van.** Each experiment was conducted in biological triplicates ($n = 3$), data points and error bars represent the mean and standard deviation across replicates.

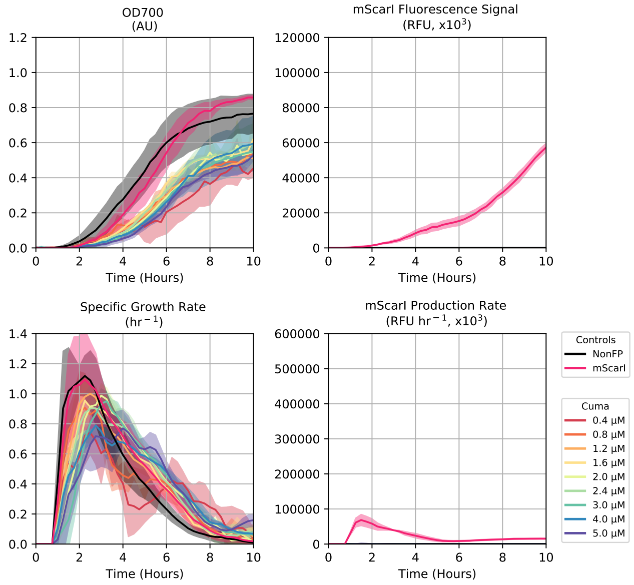

Supplementary Fig. 58. **Microplate reader data for Figure 6b corresponding to the experiments carried out in DH10B with B0034 in the presence of 0 μM Van.** Each experiment was conducted in biological triplicates ($n = 3$), data points and error bars represent the mean and standard deviation across replicates.

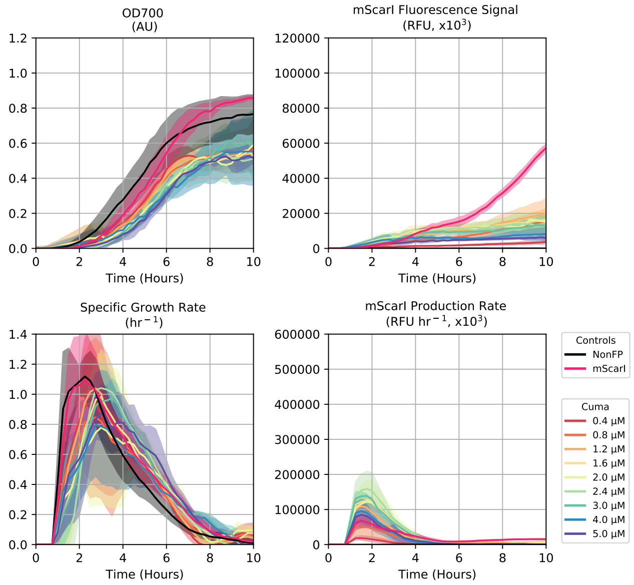

Supplementary Fig. 59. **Microplate reader data for Figure 6b corresponding to the experiments carried out in DH10B with B0034 in the presence of 100 μM Van.** Each experiment was conducted in biological triplicates ($n = 3$), data points and error bars represent the mean and standard deviation across replicates.

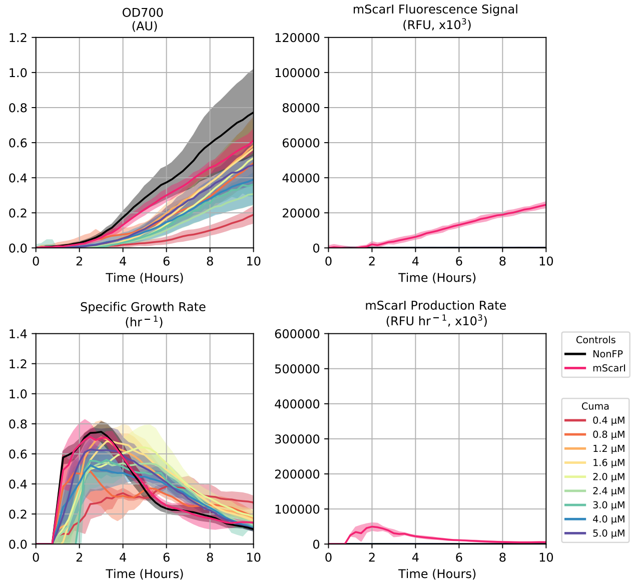

Supplementary Fig. 60. **Microplate reader data for Figure 6b corresponding to the experiments carried out in BW25113 with B0032 in the presence of 0 μM Van.** Each experiment was conducted in biological triplicates ($n = 3$), data points and error bars represent the mean and standard deviation across replicates.

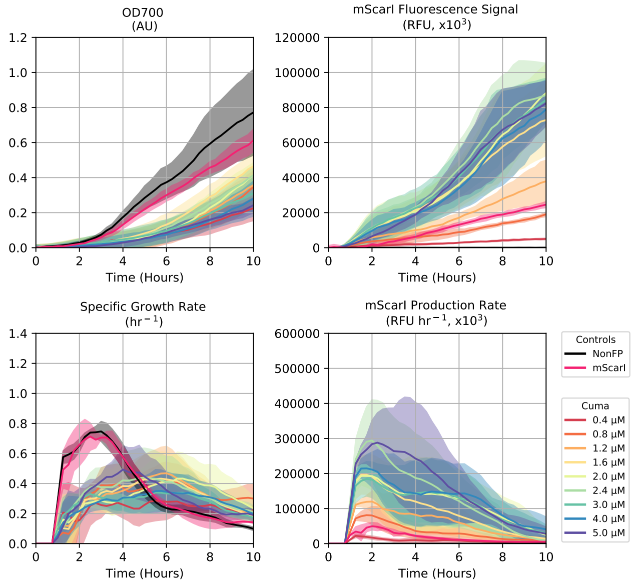

Supplementary Fig. 61. **Microplate reader data for Figure 6b corresponding to the experiments carried out in BW25113 with B0032 in the presence of 100 μM Van.** Each experiment was conducted in biological triplicates ($n = 3$), data points and error bars represent the mean and standard deviation across replicates.

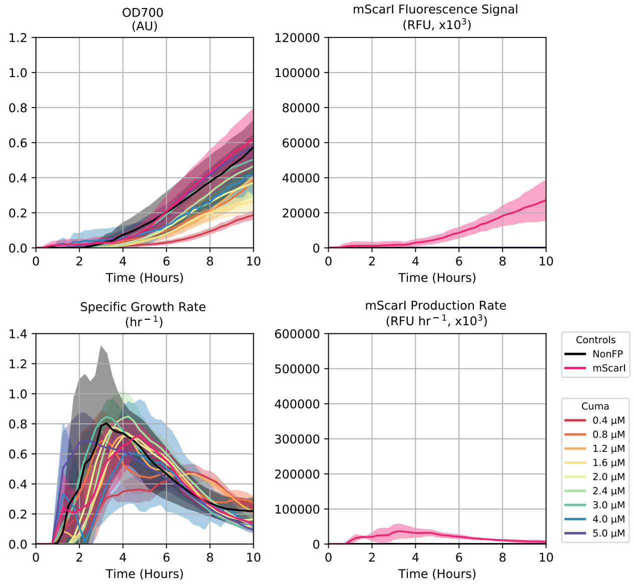

Supplementary Fig. 62. **Microplate reader data for Figure 6b corresponding to the experiments carried out in BW25113 with B0034 in the presence of 0 μM Van.** Each experiment was conducted in biological triplicates ($n = 3$), data points and error bars represent the mean and standard deviation across replicates.

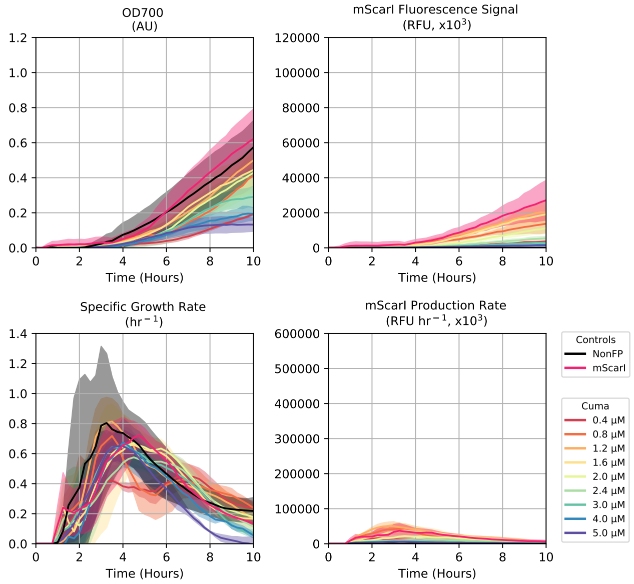

Supplementary Fig. 63. **Microplate reader data for Figure 6b corresponding to the experiments carried out in BW25113 with B0034 in the presence of 100 μM Van.** Each experiment was conducted in biological triplicates ($n = 3$), data points and error bars represent the mean and standard deviation across replicates.

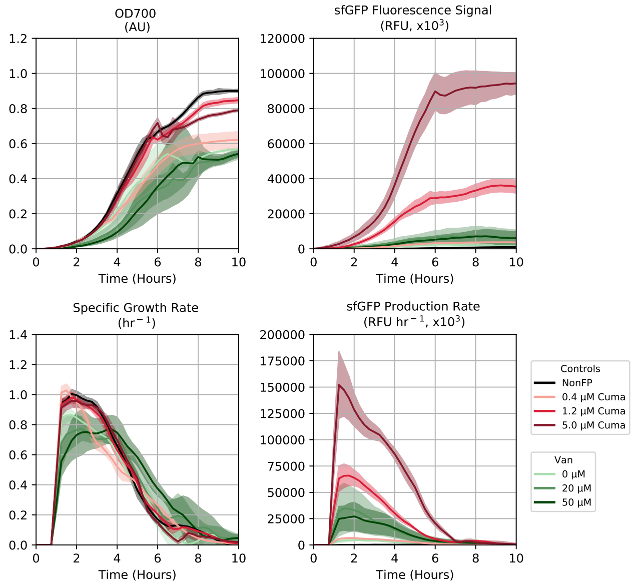

Supplementary Fig. 64. **Microplate reader data for Figure 8a corresponding to gRNA #1.** Each experiment was conducted in biological triplicates ($n = 3$), data points and error bars represent the mean and standard deviation across replicates.

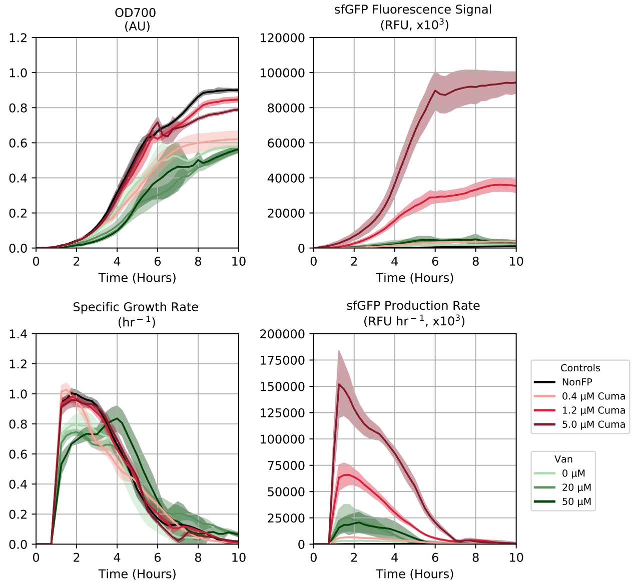

Supplementary Fig. 65. **Microplate reader data for Figure 8a corresponding to gRNA #2.** Each experiment was conducted in biological triplicates ($n = 3$), data points and error bars represent the mean and standard deviation across replicates.

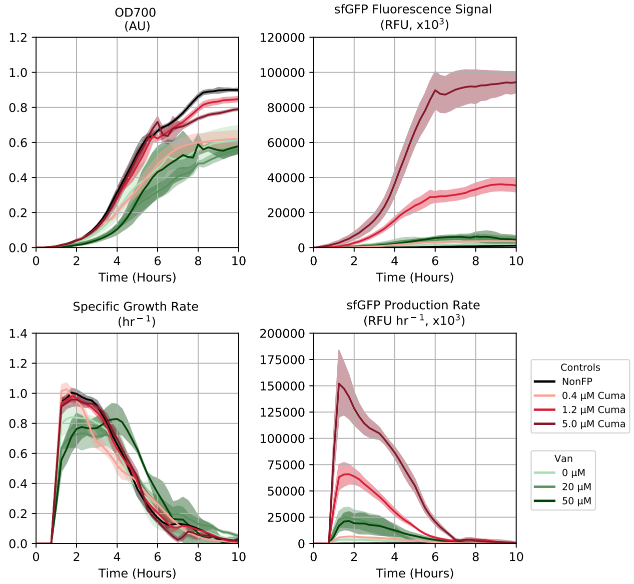

Supplementary Fig. 66. **Microplate reader data for Figure 8a corresponding to gRNA #3.** Each experiment was conducted in biological triplicates ($n = 3$), data points and error bars represent the mean and standard deviation across replicates.

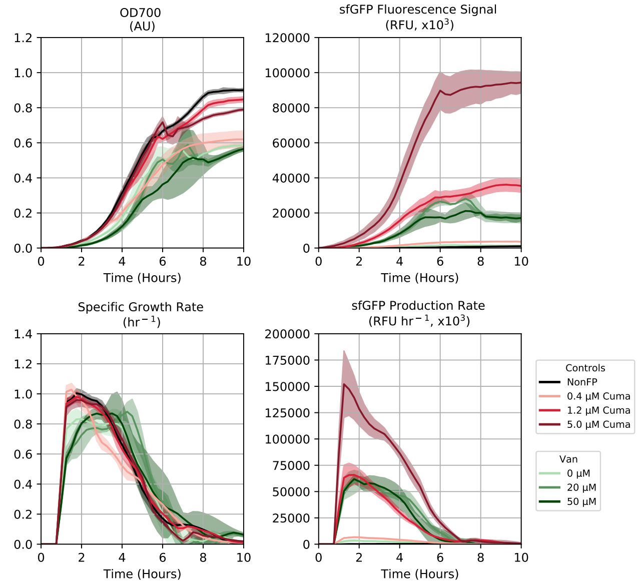

Supplementary Fig. 67. **Microplate reader data for Figure 8a corresponding to gRNA #4.** Each experiment was conducted in biological triplicates ($n = 3$), data points and error bars represent the mean and standard deviation across replicates.

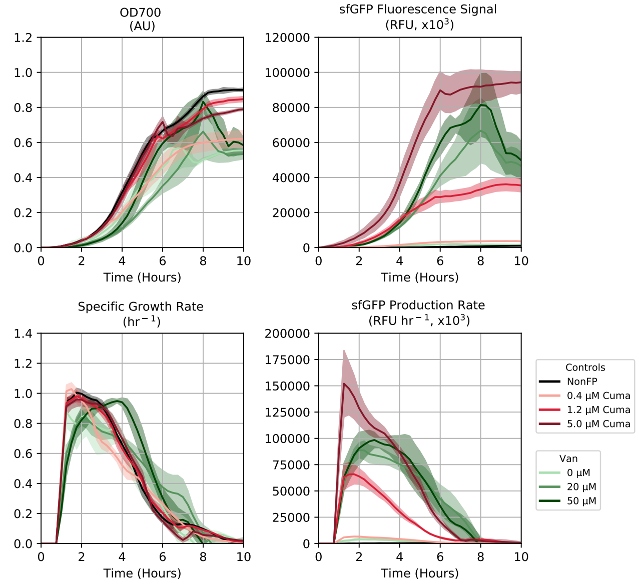

Supplementary Fig. 68. **Microplate reader data for Figure 8a corresponding to gRNA #5.** Each experiment was conducted in biological triplicates ($n = 3$), data points and error bars represent the mean and standard deviation across replicates.

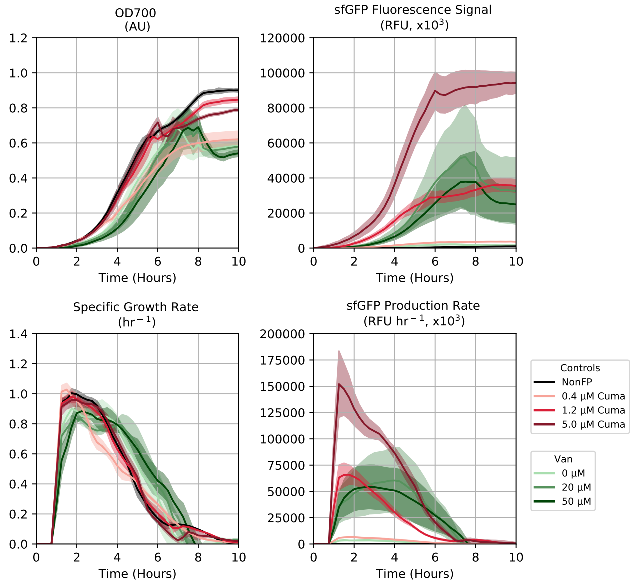

Supplementary Fig. 69. **Microplate reader data for Figure 8a corresponding to gRNA #6.** Each experiment was conducted in biological triplicates ($n = 3$), data points and error bars represent the mean and standard deviation across replicates.

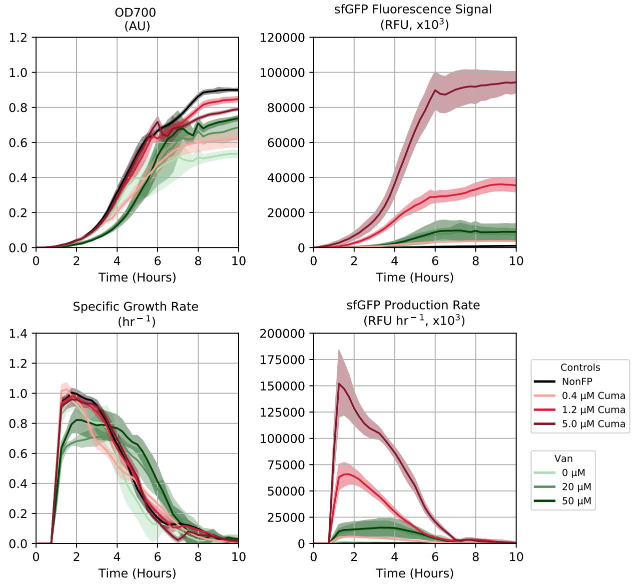

Supplementary Fig. 70. **Microplate reader data for Figure 8a corresponding to gRNA #7.** Each experiment was conducted in biological triplicates ($n = 3$), data points and error bars represent the mean and standard deviation across replicates.

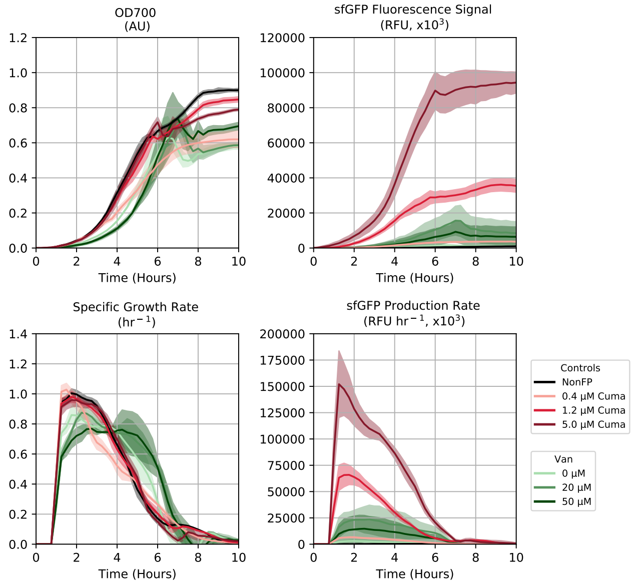

Supplementary Fig. 71. **Microplate reader data for Figure 8a corresponding to gRNA #8.** Each experiment was conducted in biological triplicates ($n = 3$), data points and error bars represent the mean and standard deviation across replicates.

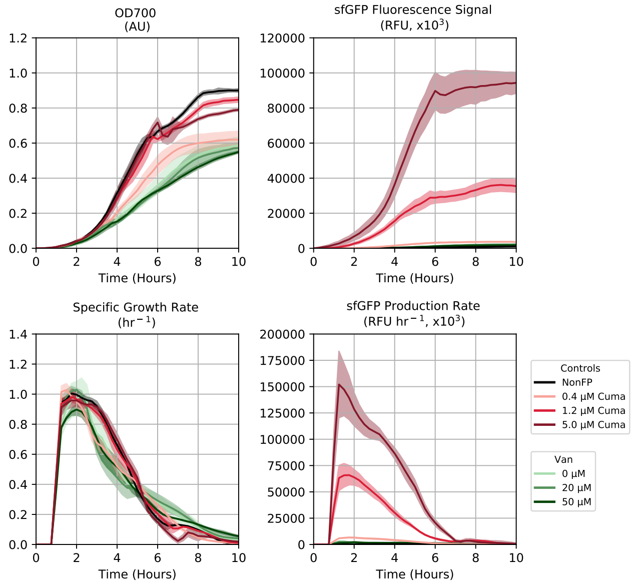

Supplementary Fig. 72. **Microplate reader data for Figure 8a corresponding to gRNA #9.** Each experiment was conducted in biological triplicates ($n = 3$), data points and error bars represent the mean and standard deviation across replicates.

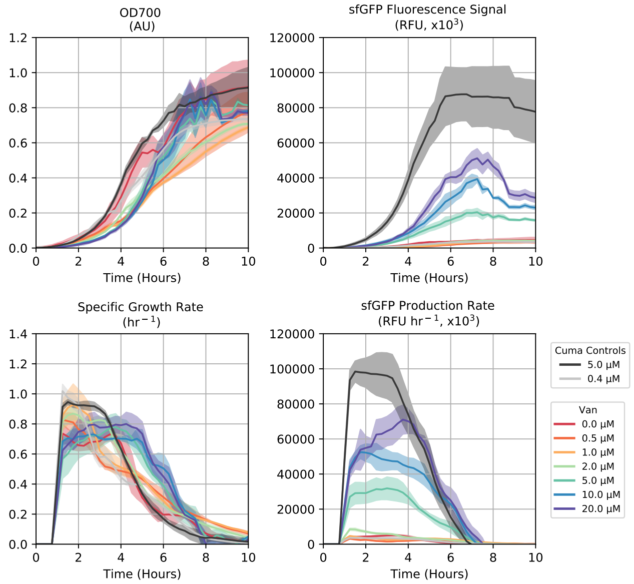

Supplementary Fig. 73. **Microplate reader data for Figure 8b corresponding to gRNA #5.** Each experiment was conducted in biological triplicates ($n = 3$), data points and error bars represent the mean and standard deviation across replicates.

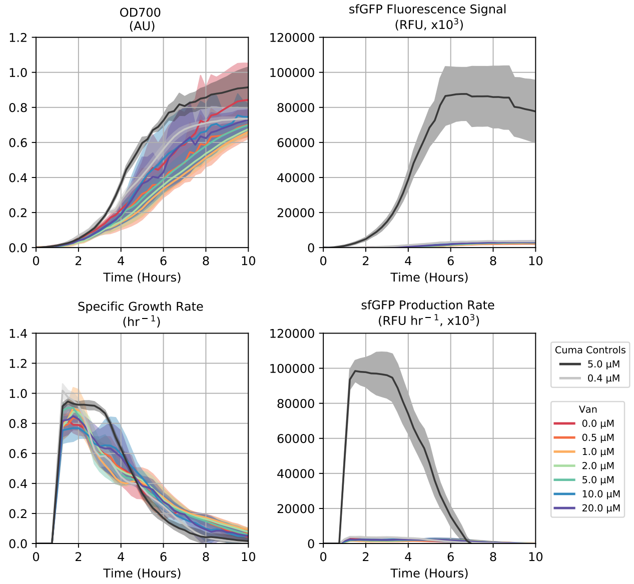

Supplementary Fig. 74. **Microplate reader data for Figure 8b corresponding to gRNA #9 (negative control).** Each experiment was conducted in biological triplicates ($n = 3$), data points and error bars represent the mean and standard deviation across replicates.

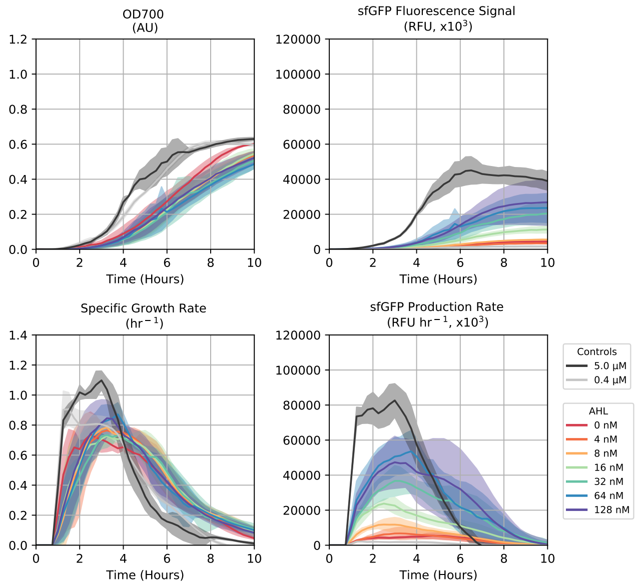

Supplementary Fig. 75. **Microplate reader data for Figure 8c corresponding to gRNA #5.** Each experiment was conducted in biological triplicates ($n = 3$), data points and error bars represent the mean and standard deviation across replicates.

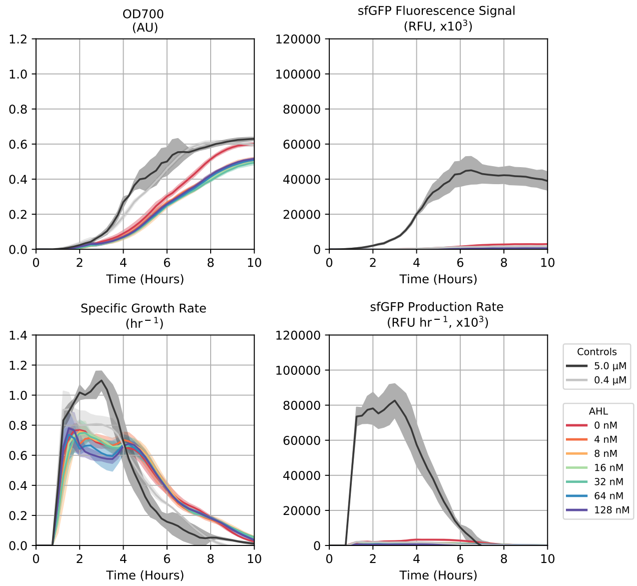

Supplementary Fig. 76. **Microplate reader data for Figure 8c corresponding to gRNA #9 (negative control).** Each experiment was conducted in biological triplicates ($n = 3$), data points and error bars represent the mean and standard deviation across replicates.

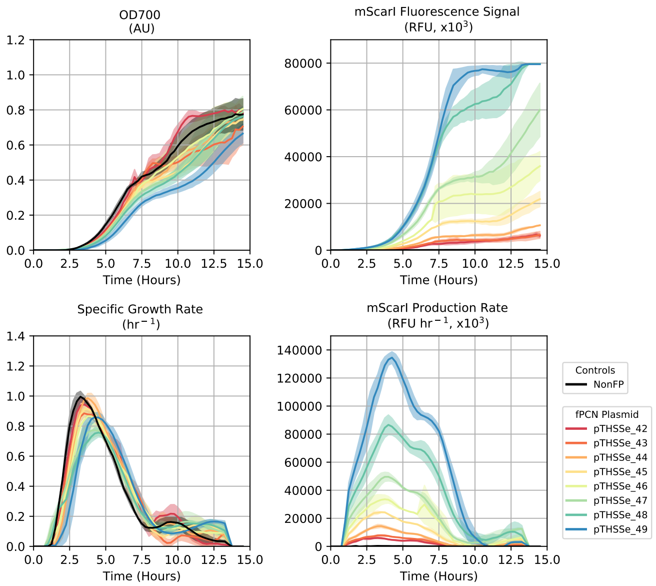

Supplementary Fig. 77. **Microplate reader data for Supplementary Fig. 1.** Each experiment was conducted in biological triplicates ($n = 3$), data points and error bars represent the mean and standard deviation across replicates.

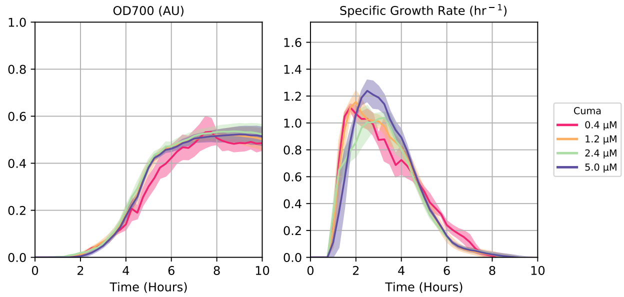

Supplementary Fig. 78. **Microplate reader data for Supplementary Fig. 7 corresponding to M9-Gluc.** Each experiment was conducted in biological triplicates ($n = 3$), data points and error bars represent the mean and standard deviation across replicates.

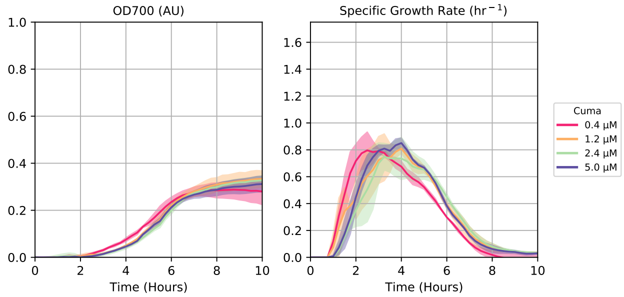

Supplementary Fig. 79. **Microplate reader data for Supplementary Fig. 7 corresponding to M9-Gly.** Each experiment was conducted in biological triplicates ($n = 3$), data points and error bars represent the mean and standard deviation across replicates.

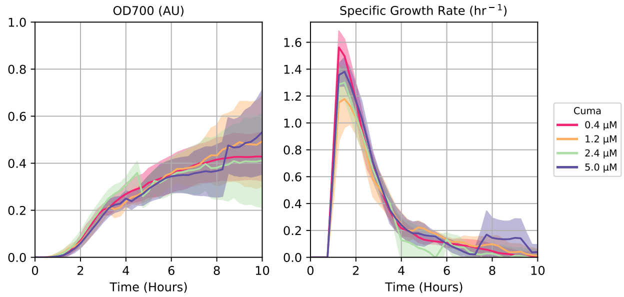

Supplementary Fig. 80. **Microplate reader data for Supplementary Fig. 7 corresponding to LB.** Each experiment was conducted in biological triplicates ($n = 3$), data points and error bars represent the mean and standard deviation across replicates.

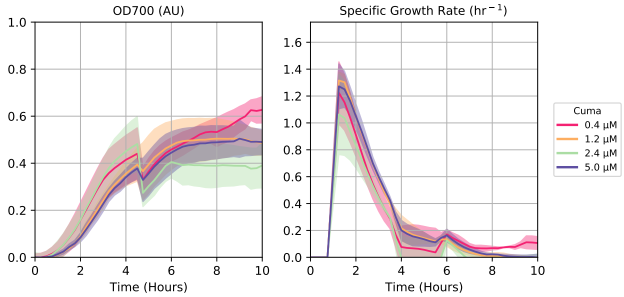

Supplementary Fig. 81. **Microplate reader data for Supplementary Fig. 7 corresponding to SOB.** Each experiment was conducted in biological triplicates ($n = 3$), data points and error bars represent the mean and standard deviation across replicates.

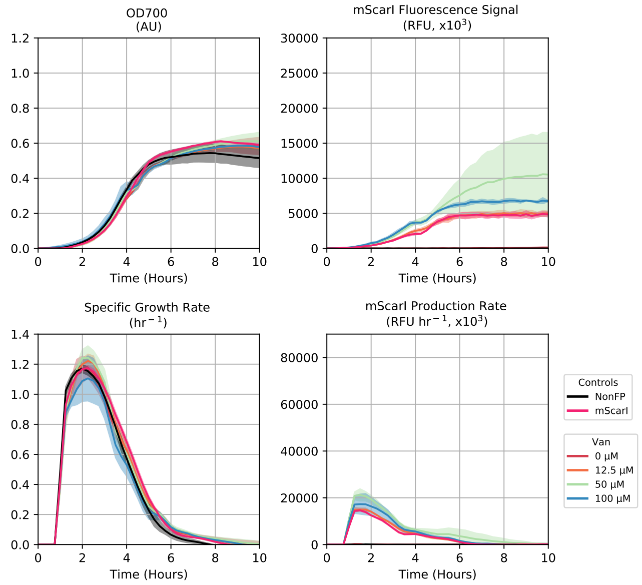

Supplementary Fig. 82. **Microplate reader data for Supplementary Fig. 13 corresponding to the experiments carried out with pSC101.** Each experiment was conducted in biological triplicates ($n = 3$), data points and error bars represent the mean and standard deviation across replicates.

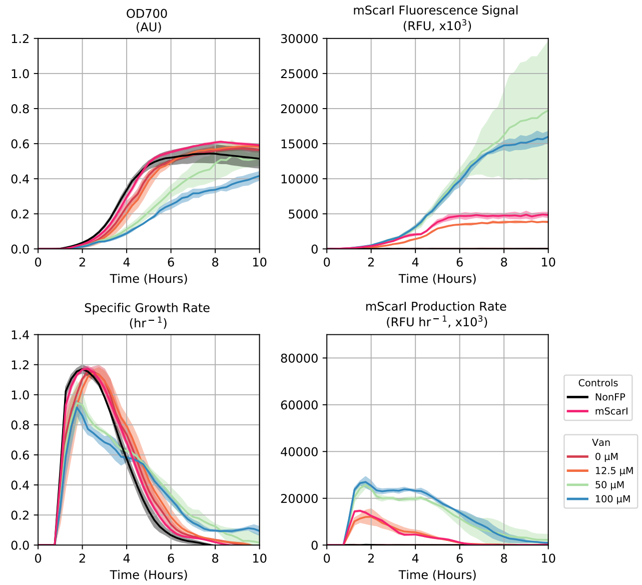

Supplementary Fig. 83. **Microplate reader data for Supplementary Fig. 13 corresponding to the experiments carried out with p15A.** Each experiment was conducted in biological triplicates ($n = 3$), data points and error bars represent the mean and standard deviation across replicates.

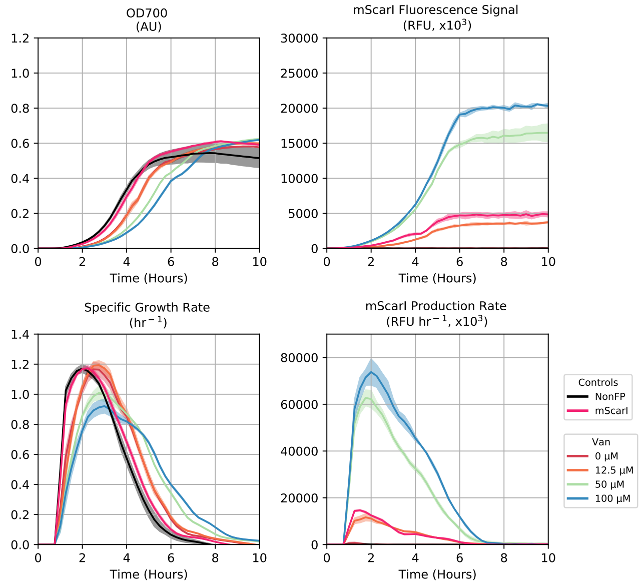

Supplementary Fig. 84. **Microplate reader data for Supplementary Fig. 13 corresponding to the experiments carried out with pColE1.** Each experiment was conducted in biological triplicates ($n = 3$), data points and error bars represent the mean and standard deviation across replicates.

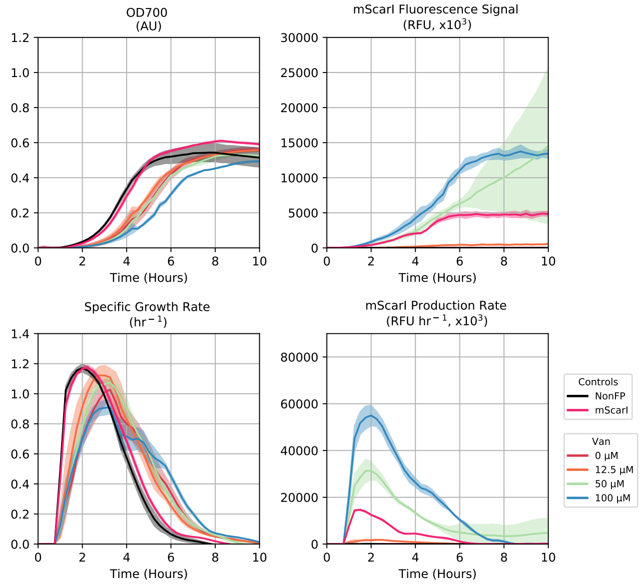

Supplementary Fig. 85. **Microplate reader data for Supplementary Fig. 13 corresponding to the experiments carried out with pUC19.** Each experiment was conducted in biological triplicates ($n = 3$), data points and error bars represent the mean and standard deviation across replicates.

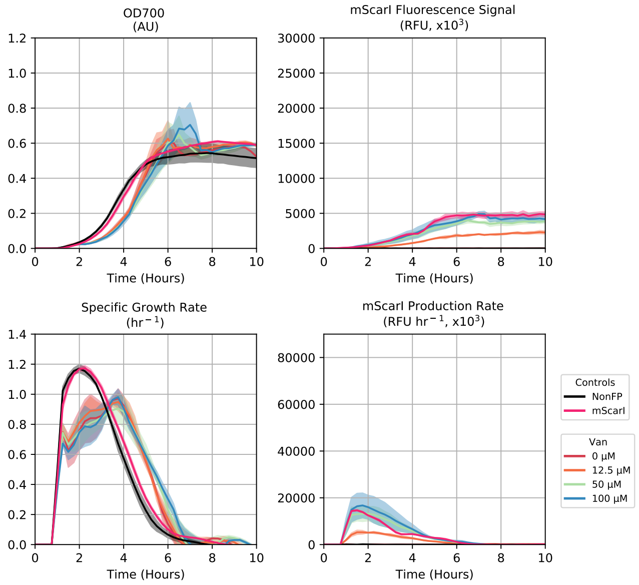

Supplementary Fig. 86. **Microplate reader data for Supplementary Fig. 13 corresponding to the experiments carried out with TULIP in the presence of 0.8 μM Cuminic acid.** Each experiment was conducted in biological triplicates ($n = 3$), data points and error bars represent the mean and standard deviation across replicates.

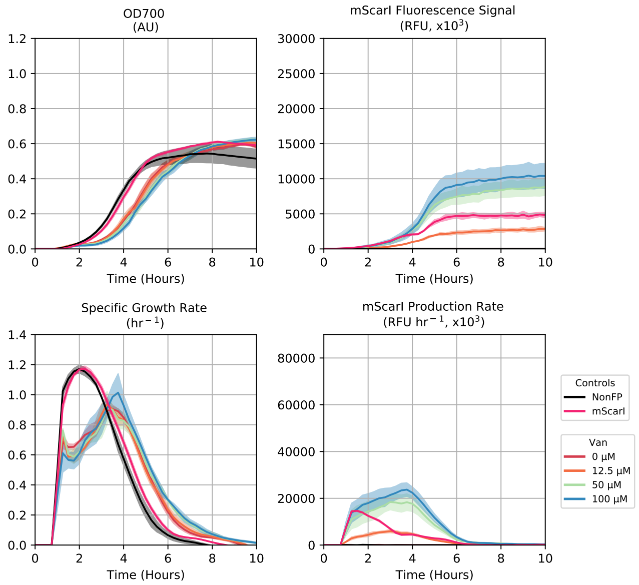

Supplementary Fig. 87. **Microplate reader data for Supplementary Fig. 13 corresponding to the experiments carried out with TULIP in the presence of 1.6 μM Cuminic acid.** Each experiment was conducted in biological triplicates ($n = 3$), data points and error bars represent the mean and standard deviation across replicates.

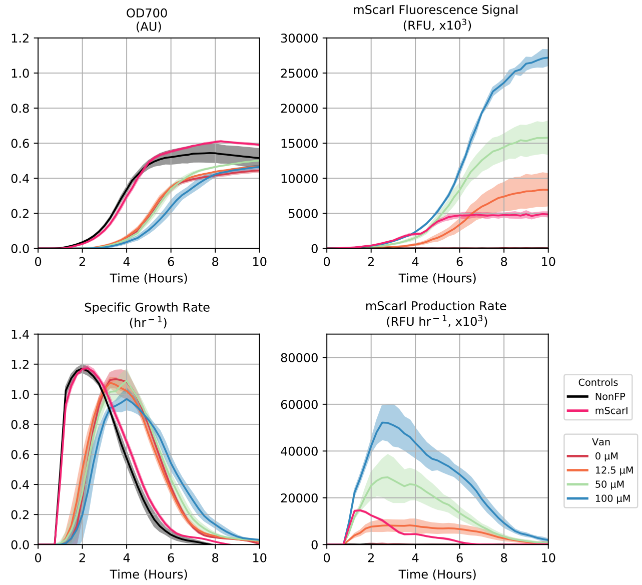

Supplementary Fig. 88. **Microplate reader data for Supplementary Fig. 13 corresponding to the experiments carried out with TULIP in the presence of 2.4 μM Cuminic acid.** Each experiment was conducted in biological triplicates ($n = 3$), data points and error bars represent the mean and standard deviation across replicates.

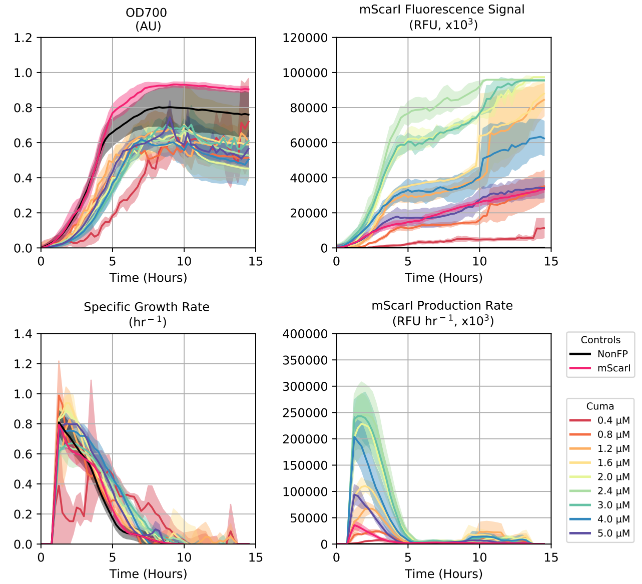

Supplementary Fig. 89. **Microplate reader data for Supplementary Fig. 14 corresponding to the experiments carried out in DH10B in the presence of 0 μM Vanillic acid.** Each experiment was conducted in biological triplicates ($n = 3$), data points and error bars represent the mean and standard deviation across replicates.

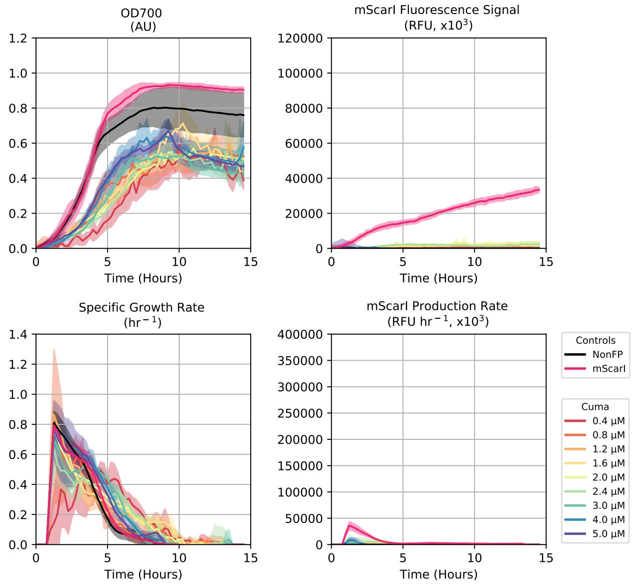

Supplementary Fig. 90. **Microplate reader data for Supplementary Fig. 14 corresponding to the experiments carried out in DH10B in the presence of 100 μM Vanillic acid.** Each experiment was conducted in biological triplicates ($n = 3$), data points and error bars represent the mean and standard deviation across replicates.

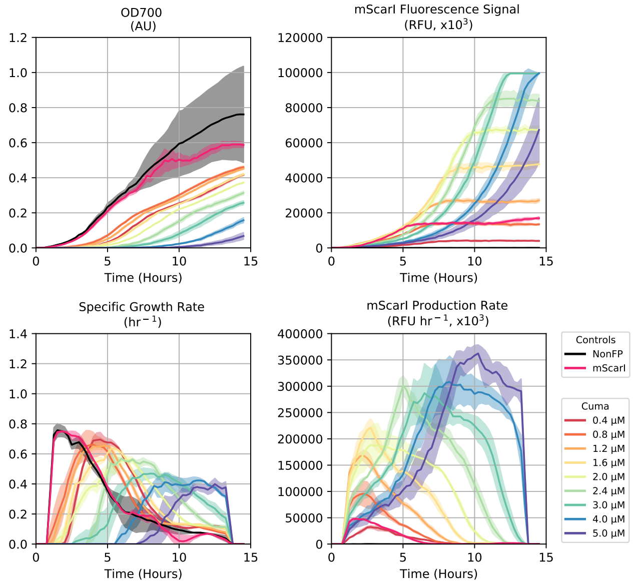

Supplementary Fig. 91. **Microplate reader data for Supplementary Fig. 14 corresponding to the experiments carried out in BW25113 in the presence of 0 μM Vanillic acid.** Each experiment was conducted in biological triplicates ($n = 3$), data points and error bars represent the mean and standard deviation across replicates.

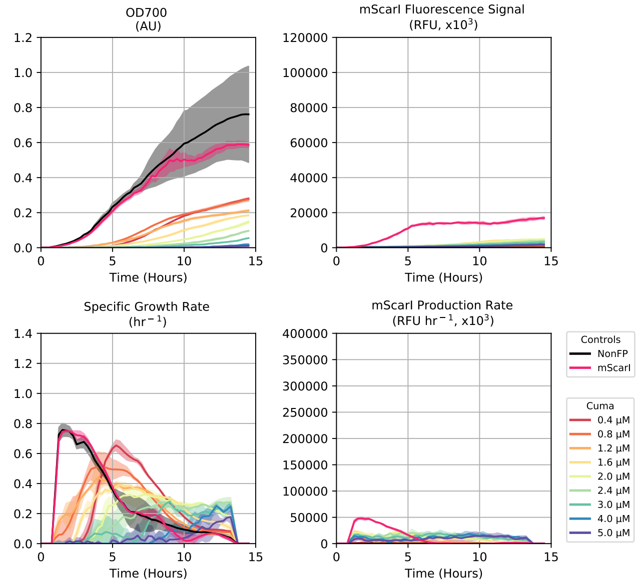

Supplementary Fig. 92. **Microplate reader data for Supplementary Fig. 14 corresponding to the experiments carried out in BW25113 in the presence of 100 μM Vanillic acid.** Each experiment was conducted in biological triplicates ($n = 3$), data points and error bars represent the mean and standard deviation across replicates.

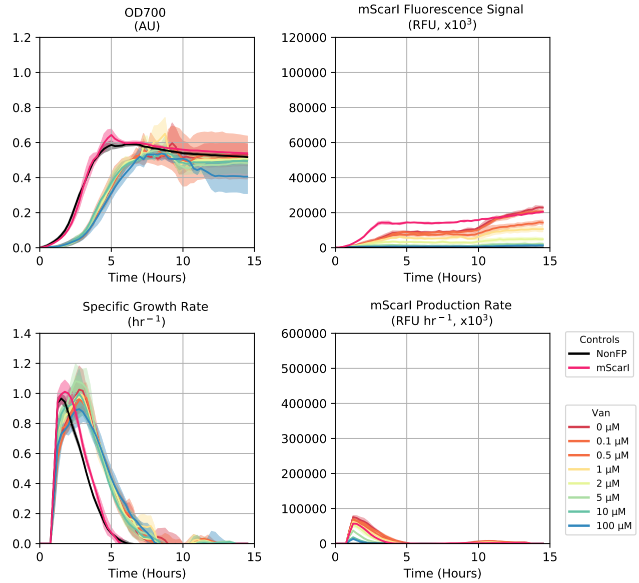

Supplementary Fig. 93. **Microplate reader data for Supplementary Fig. 14 corresponding to the experiments carried out in DH10B in the presence of 0.8 μM Cuma.** Each experiment was conducted in biological triplicates ($n = 3$), data points and error bars represent the mean and standard deviation across replicates.

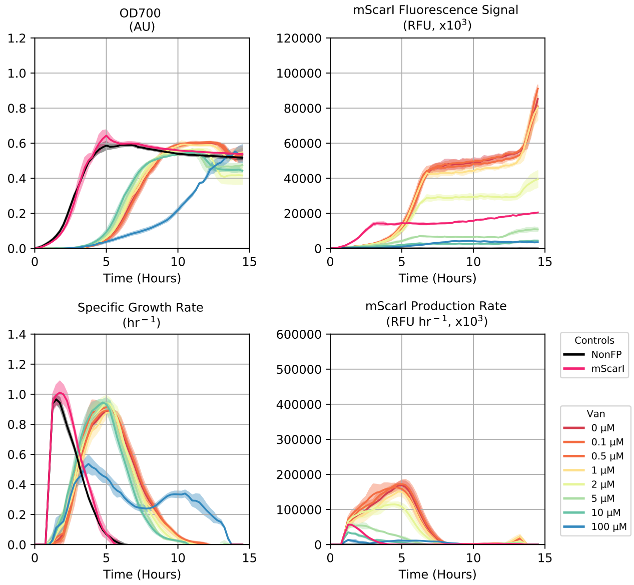

Supplementary Fig. 94. **Microplate reader data for Supplementary Fig. 14 corresponding to the experiments carried out in DH10B in the presence of 1.6 μM Cuma.** Each experiment was conducted in biological triplicates ($n = 3$), data points and error bars represent the mean and standard deviation across replicates.

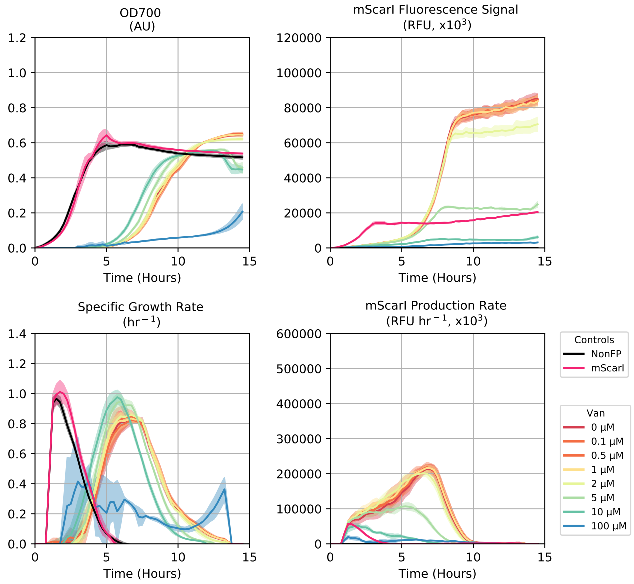

Supplementary Fig. 95. **Microplate reader data for Supplementary Fig. 14 corresponding to the experiments carried out in DH10B in the presence of 2.4 μM Cuma.** Each experiment was conducted in biological triplicates ($n = 3$), data points and error bars represent the mean and standard deviation across replicates.

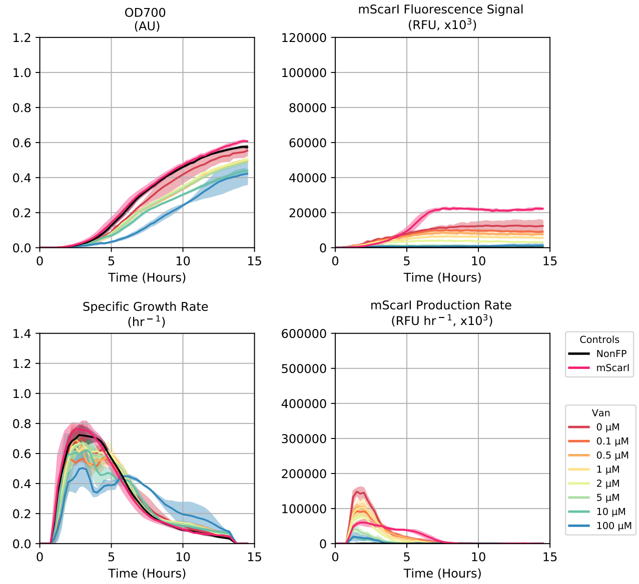

Supplementary Fig. 96. **Microplate reader data for Supplementary Fig. 14 corresponding to the experiments carried out in BW25113 in the presence of 0.8 μM Cuma.** Each experiment was conducted in biological triplicates ($n = 3$), data points and error bars represent the mean and standard deviation across replicates.

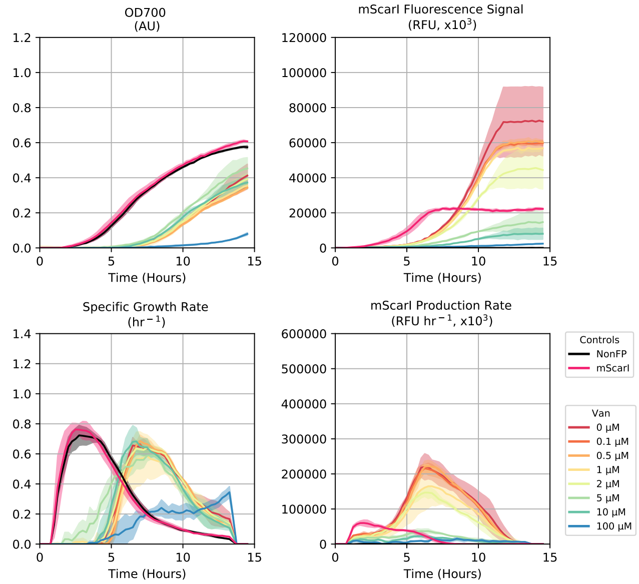

Supplementary Fig. 97. **Microplate reader data for Supplementary Fig. 14 corresponding to the experiments carried out in BW25113 in the presence of 1.6 μM Cuma.** Each experiment was conducted in biological triplicates ($n = 3$), data points and error bars represent the mean and standard deviation across replicates.

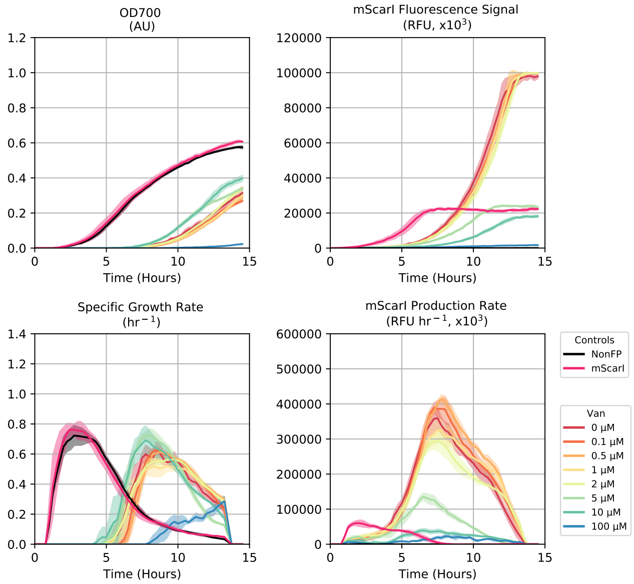

Supplementary Fig. 98. **Microplate reader data for Supplementary Fig. 14 corresponding to the experiments carried out in BW25113 in the presence of 2.4 μM Cuma.** Each experiment was conducted in biological triplicates ($n = 3$), data points and error bars represent the mean and standard deviation across replicates.

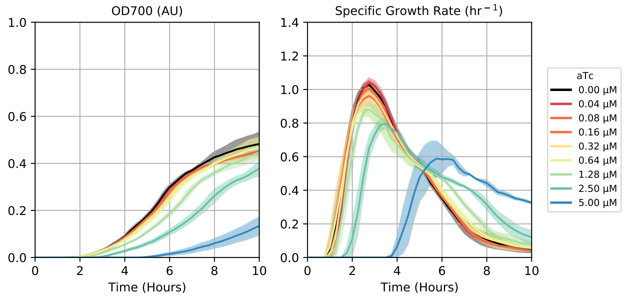

Supplementary Fig. 99. **Microplate reader data for Supplementary Fig. 26a.** Each experiment was conducted in biological triplicates ($n = 3$), data points and error bars represent the mean and standard deviation across replicates.

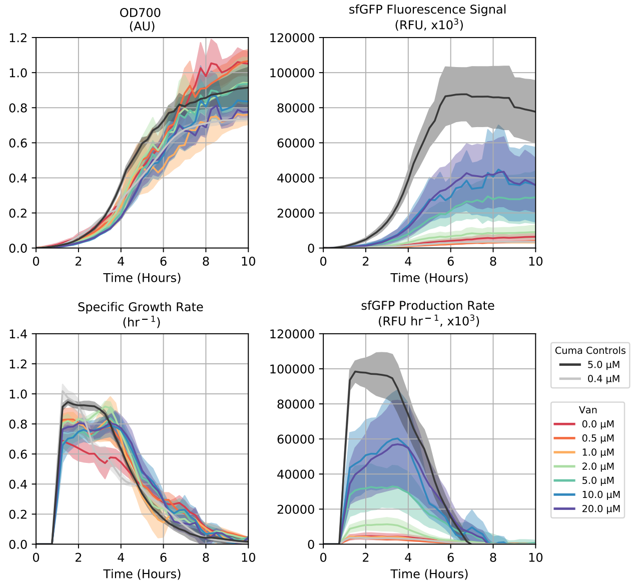

Supplementary Fig. 100. **Microplate reader data for Supplementary Fig. 26b.** Each experiment was conducted in biological triplicates ($n = 3$), data points and error bars represent the mean and standard deviation across replicates.

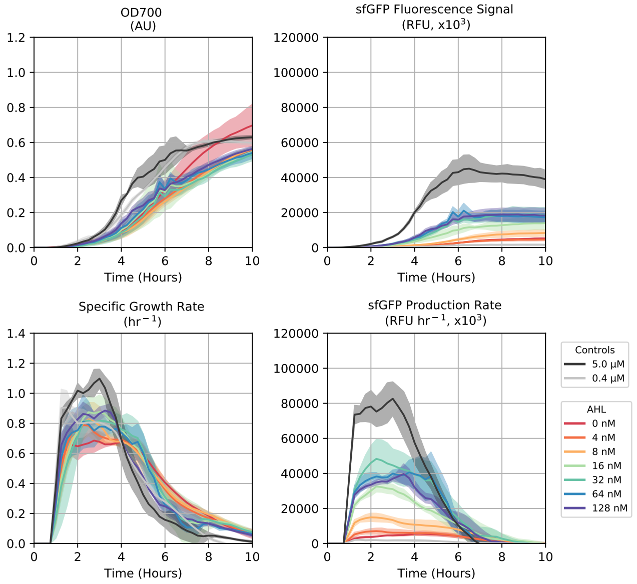

Supplementary Fig. 101. **Microplate reader data for Supplementary Fig. 26c.** Each experiment was conducted in biological triplicates ($n = 3$), data points and error bars represent the mean and standard deviation across replicates.

2 Strains, genetic parts, constructs, and primers

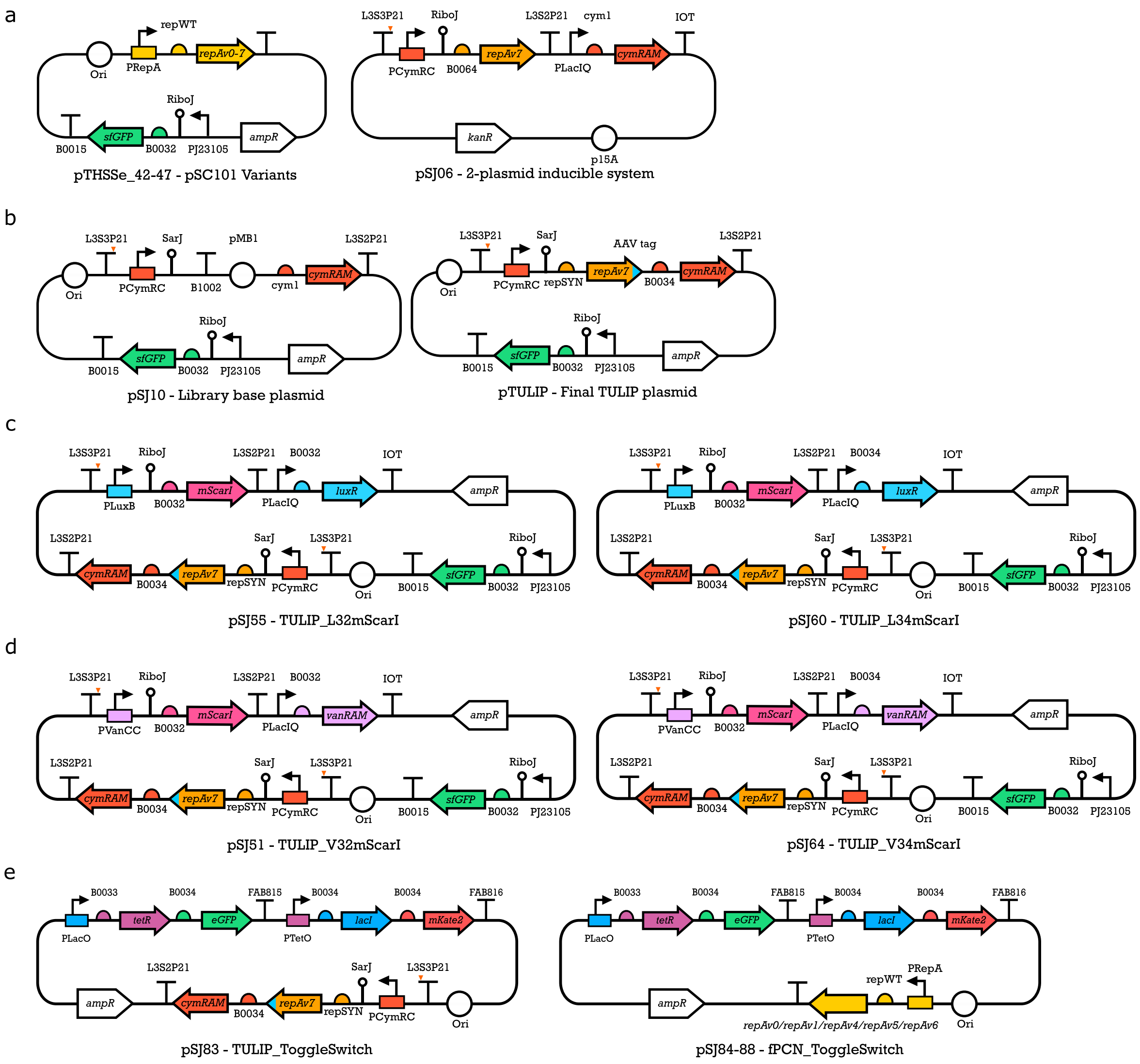

Supplementary Fig. 102. **Key plasmid maps of constructs used (part 1 of 2).** Orange triangles above terminators indicate removal of BsaI restriction site from the original part sequence, see Supplementary Table 6. **a** The fPCN collection and the 2-plasmid inducible PCN system constructs used in Figure 1 and Supplementary Fig. 1. **b** Base plasmid used to construct the RBS libraries and final TULIP design used in Figures 2–3 and Supplementary Fig. 2. **c** AHL-inducible sensor module used in Figure 5a and Figure 6a. **d** Van-inducible sensor module used in Figure 5b and Figure 6b. **e** Toggle switch harbored in TULIP and in plasmids with fixed copy number used in Figure 7.

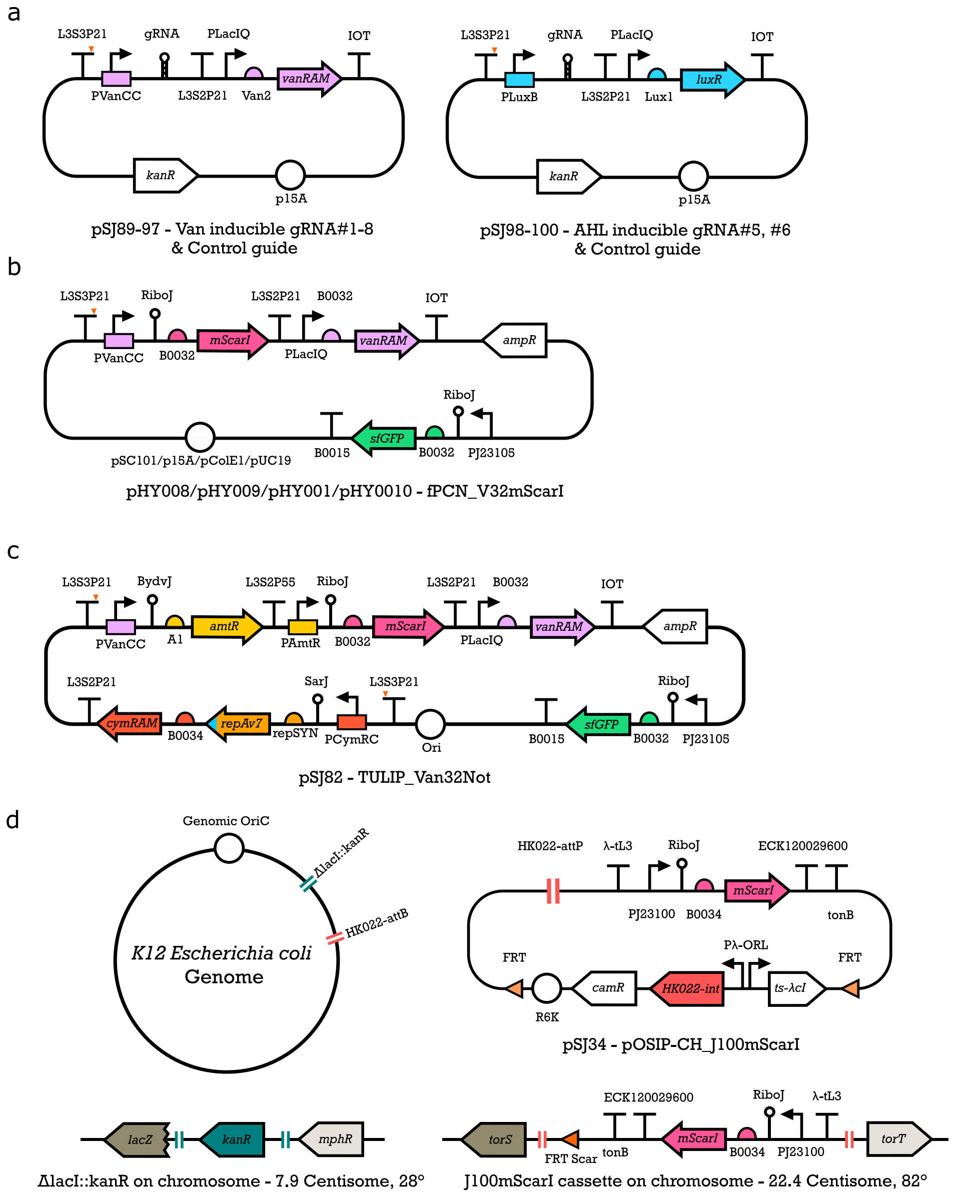

Supplementary Fig. 103. **Key plasmid maps of constructs used (part 2 of 2).** Orange triangles above terminators indicate removal of BsaI restriction site from the original part sequence, see Supplementary Table 6. **a** CRISPRi-based additional layer of control used in Figure 8. **b** Inducible mScarI constructs used in Supplementary Fig. 13. **c** Van-inducible NOT gate used in Supplementary Fig. 14. **d** *E. coli* genome map indicating approximate locations of where modifications made when appropriate. pSJ34 was used to create mScarI expressing control strains; HK022 integration site occurs between the diverging genes torS and torT. The selection marker is cured from the genome using the pE-FLP plasmid as instructed in (*11*), leaving only an FRT scar in its place and the J100mScarI cassette insertion. The ΔlacI::kanR modification was made to DH10B using lambda-RED/pREDCas9.

Supplementary Table 1: Strains used in the paper

Strains	Genotype	Strain Source
NEBStable	K-12 F' proA+B+ lacIq Δ(lacZ)M15 zzf::Tn10 (TetR)/ Δ(ara-leu) 7697 araD139 fhuA ΔlacX74 galK16 galE15 e14- Φ80dlacZΔM15 recA1 relA1 endA1 nupG rpsL (StrR) rph spoT1 Δ(mrr-hsdRMS-mcrBC)	NEB
NEB10Beta	K-12 Δ(ara-leu) 7697 araD139 fhuA ΔlacX74 galK16 galE15 e14- ϕ80dlacZΔM15 recA1 relA1 endA1 nupG rpsL (StrR) rph spoT1 Δ(mrr-hsdRMS-mcrBC)	NEB
NEBExpress	B-Strain fhuA2 [lon] ompT gal sulA11 R(mcr-73::miniTn10--TetS)2 [dcm] R(zgb-210::Tn10--TetS) endA1 Δ(mcrC-mrr)114::IS10	NEB
MG1655	K-12 F– λ– ilvG– rfb-50 rph-1 ΔlacI	Lee *et al.* 2016
BW25113	K-12 ΔfliA ΔlacY ΔacrAB lacI^{+}rrnB$_{T14}$ ΔlacZ$_{WJ16}$ hsdR514 ΔaraBAD$_{AH33}$ ΔrhaBAD$_{LD78}$ rph-1 Δ(araB–D)567 Δ(rhaD–B)568 ΔlacZ4787(::rrnB-3) hsdR514 rph-1	Lugagne *et al.* 2017
SM10(λpir)	K-12 thi thr leu tonA lacY supE recA::RP4-2-Tc::Mu Km λpir	Lee *et al.* 2016
FR-E01	K-12 MG1655 derivative F– λ– ilvG– rfb-50 rph-1 HK022 attB:pOSIP-KH-RBS2-dCas9 (aTc inducible dCas9)	Rousset *et al.* 2018
DHScarI	NEB10Beta with HK022 attB:PJ23100_RiboJ_B0034_mScarI_ECK120029600	This work
BWScarI	BW25113 with HK022 attB:PJ23100_RiboJ_B0034_mScarI_ECK120029600	This work
DH10B(ΔlacI)	NEB10Beta with ΔlacI::kanR	This work

Supplementary Table 2: Plasmids used in the paper

Plasmid ID	Description	Plasmid source
pTHSSe_42	pSC101 RepA variant 0 (wildtype) with constitutive sfGFP, pSC101_repAv0, AmpR	Segall-Shapiro *et al.* 2018
pTHSSe_43	pSC101 RepA variant 1 (E93V) with constitutive sfGFP, pSC101_repAv1, AmpR	Segall-Shapiro *et al.* 2018
pTHSSe_44	pSC101 RepA variant 2 (E93W) with constitutive sfGFP, pSC101_repAv2, AmpR	Segall-Shapiro *et al.* 2018
pTHSSe_45	pSC101 RepA variant 3 (E93K) with constitutive sfGFP, pSC101_repAv3, AmpR	Segall-Shapiro *et al.* 2018
pTHSSe_46	pSC101 RepA variant 4 (E93G) with constitutive sfGFP, pSC101_repAv4, AmpR	Segall-Shapiro *et al.* 2018
pTHSSe_47	pSC101 RepA variant 5 (R43W, T304I) with constitutive sfGFP, pSC101_repAv5, AmpR	Segall-Shapiro *et al.* 2018
pTHSSe_48	pSC101 RepA variant 6 (N99D) with constitutive sfGFP, pSC101_repAv6, AmpR	Segall-Shapiro *et al.* 2018
pTHSSe_49	pSC101 RepA variant 7 (N99D, E177G) with constitutive sfGFP, pSC101_repAv7, AmpR	Segall-Shapiro *et al.* 2018
pSJ06	Cuminic acid inducible repAv7, p15A Ori, KanR	This work
pSJ10	Library base plasmids with constitutive sfGFP expression, pMB1 Ori, AmpR	This work
pTULIP	Final Tunable Ligand Inducible Plasmid with AAV tag, TULIP Ori, AmpR	This work
pTULIP-LVA	Tunable Ligand Inducible Plasmid with LVA tag, TULIP Ori, AmpR	This work
pSJ34	pOSIP-CH modified with J100mScarI insert	This work
pSJ51	Vanillic acid inducible mScarlet-I circuit on TULIP, VanRAM has a moderate-weak B0032 RBS, TULIP Ori, AmpR	This work
pSJ55	3O6C-HSL inducible mScarlet-I circuit on TULIP, LuxR has a moderate-weak B0032 RBS, TULIP Ori, AmpR	This work
pSJ60	3O6C-HSL inducible mScarlet-I circuit on TULIP, LuxR has a strong B0034 RBS, TULIP Ori, AmpR	This work
pSJ64	Vanillic acid inducible mScarlet-I circuit on TULIP, VanRAM has a strong B0034 RBS, TULIP Ori, AmpR	This work
pSJ82	Vanillic acid inducible AmtR-mScarI Not gate circuit on TULIP, VanRAM has a moderate-weak B0032 RBS, TULIP Ori, AmpR	This work
pHY008	Vanillic acid inducible mScarlet-I circuit on TULIP, VanRAM has a moderate-weak B0032 RBS, pSC101 (wildtype) Ori, AmpR	This work
pHY009	Vanillic acid inducible mScarlet-I circuit on TULIP, VanRAM has a moderate-weak B0032 RBS, p15A Ori, AmpR	This work
pHY001	Vanillic acid inducible mScarlet-I circuit on TULIP, VanRAM has a moderate-weak B0032 RBS, ColE1 Ori, AmpR	This work
pHY010	Vanillic acid inducible mScarlet-I circuit on TULIP, VanRAM has a moderate-weak B0032 RBS, pUC19 Ori, AmpR	This work
pSJ83	IPTG and aTc inducible Lugagne Toggle switch on TULIP, TULIP Ori, AmpR	This work
pSJ84	IPTG and aTc inducible Lugagne Toggle switch on pTHSSe_42, pSC101v1, AmpR	This work
pSJ85	IPTG and aTc inducible Lugagne Toggle switch on pTHSSe_43, pSC101v1, AmpR	
pSJ86	IPTG and aTc inducible Lugagne Toggle switch on pTHSSe_46, pSC101v1, AmpR	

pSJ87	IPTG and aTc inducible Lugagne Toggle switch on pTHSSe_47, pSC101v5, AmpR	This work
pSJ88	IPTG and aTc inducible Lugagne Toggle switch on pTHSSe_48, pSC101v6, AmpR	This work
pSJ89	Vanillic acid inducible gRNA #1 targeting CymRAM CDS on TULIP, p15A Ori, KanR	This work
pSJ90	Vanillic acid inducible gRNA #2 targeting CymRAM CDS on TULIP, p15A Ori, KanR	This work
pSJ91	Vanillic acid inducible gRNA #3 targeting CymRAM CDS on TULIP, p15A Ori, KanR	This work
pSJ92	Vanillic acid inducible gRNA #4 targeting CymRAM CDS on TULIP, p15A Ori, KanR	This work
pSJ93	Vanillic acid inducible gRNA #5 targeting CymRAM CDS on TULIP, p15A Ori, KanR	This work
pSJ94	Vanillic acid inducible gRNA #6 targeting CymRAM CDS on TULIP, p15A Ori, KanR	This work
pSJ95	Vanillic acid inducible gRNA #7 targeting CymRAM CDS on TULIP, p15A Ori, KanR	This work
pSJ96	Vanillic acid inducible gRNA #8 targeting CymRAM CDS on TULIP, p15A Ori, KanR	This work
pSJ97	Vanillic acid inducible gRNA #9 Control non-targeting gRNA, p15A Ori, KanR	This work
pSJ98	3O6C-HSL inducible gRNA #5 targeting CymRAM CDS on TULIP, p15A Ori, KanR	This work
pSJ99	3O6C-HSL inducible gRNA #6 targeting CymRAM CDS on TULIP, p15A Ori, KanR	This work
pSJ100	3O6C-HSL inducible gRNA#9 Control non-targeting gRNA, p15A Ori, KanR	This work

Supplementary Table 3: Promoter parts

Part name	Part sequence	Sequence Source
PJ23105	tttacggctagctcagtcctaggtactatgctagc	iGEM
PJ23100	ttgacggctagctcagtcctaggtacagtgctagc	iGEM
PCymRC	aacaaacagacaatctggtctgtttgtattatggaaaatttttctgtataatagattcaacaaacagacaatctggtctgtttgtattat	Meyers *et al.* 2019
PVanCC	attggatccaattgacagctagctcagtcctaggtaccattggatccaat	Meyers *et al.* 2019
PLuxB	acctgtaggatcgtacaggtttacgcaagaaaatggtttgttacagtcgaataaa	Meyers *et al.* 2019
PLacIQ	gcggcgcgccatcgaatggtgcaaaacctttcgcggtatggcatgatagcgccc	Meyers *et al.* 2019
PAmtR	cttgtccaaccaaatgattcgttaccaattgacagtttctatcgatctatagataatgctagc	Nielsen *et al.* 2016
PLacO	ttgtgagcggataacaattgacattgtgagcggataacaagatactgagcacatact	Lugagne *et al.* 2017
PTetO	tccctatcagtgatagagattgacatccctatcagtgatagagatactgagcactact	Lugagne *et al.* 2017

Keys: Green indicates -10 and -35 sites; Red indicates transcription start site; Underlined indicates operator sequence

Supplementary Table 4: Ribosome binding sequence (RBS) and other RNA parts

Part name	Part sequence	Sequence Source
B0034	tactagagaaagaggagaaatactaa	iGEM
B0064	tactagagaaagaggggaaatactag	iGEM
B0032	tactagagtcacacaggaaagtactaa	iGEM
B0033	tactagagtcacacaggactactaa	iGEM
cym1	ggaagagagtcaattcagggtggtgaat	Meyers *et al.* 2019
A1	aatgttccctaataatcagcaaagaggttactag	Nielsen *et al.* 2016
repSYN	tcacactatgctttactag	This work
RiboJ	agctgtcaccggatgtgctttccggtctgatgagtccgtgaggacgaaacagcctctacaaataattttgtttaa	Lou *et al.* 2012
SarJ	agactgtcgccggatgtgtatccgacctgacgatggcccaaaagggccgaaacagtcctctacaaataattttgtttaa	Lou *et al.* 2012
BydvJ	agggtgtctcaaggtgcgtaccttgactgatgagtccgaaaggacgaaacacccctctacaaataattttgtttaa	Nielsen *et al.* 2016
gRNA scaffold	nnnnnnnnnnnnnnnnnnnngttttagagctagaaatagcaagttaaaataaggctagtccgttatcaacttgaaaaagtg gcaccgagtcggtgcttttttaggt	*CIDAR MoClo Extension, Volume I

Keys: Green indicates Shine-Dalgarno sequence; Underlined indicates core ribozyme/ core secondary structures; Blue indicates stability hairpin
** CIDAR MoClo Extension, Volume I was a gift from Richard Murray (Addgene kit #1000000161).*

Supplementary Table 5: Protein coding sequence parts

Part name	Part sequence	Sequence Source
sfGFP	atgagcaaaggagaagaacttttcactggagttgtcccaattcttgttgaattagatggtgatgttaatgggcacaaattttctgtccgtggagagggtgaaggtgatgctacaaacggaaaactcacccttaaatttatttgcactactggaaaactacctgttccgtggccaacacttgtcactactctgacctatggtgttcaatgcttttcccgttatccggatcacatgaaacggcatgactttttcaagagtgccatgcccgaaggttatgtacaggaacgcactatatctttcaaagatgacgggacctacaagacgcgtgctgaagtcaagtttgaaggtgatacccttgttaatcgtatcgagttaaagggtattgattttaaagaagatggaaacattcttggacacaaactcgagtacaactttaactcacacaatgtatacatcacggcagacaaacaaaagaatggaatcaaagctaacttcaaaattcgccacaacgttgaagatggttccgttcaactagcagaccattatcaacaaaatactccaattggcgatggccctgtccttttaccagacaaccattacctgtcgacacaatctgtcctttcgaaagatcccaacgaaaagcgtgaccacatggtccttcttgagtttgtaactgctgctgggattacacatggcatggatgagctctacaaataa	Segall-Shapiro *et al.* 2018
mScarlet-I	atggtcagtaaaggcgaagcagttatcaaagagttcatgcgcttcaaagttcatatggaagggtcgatgaacgggcacgaatttgaaattgaaggcgaaggcgaaggccgcccatatgaagggacccaaaccgcaaagcttaaggttactaaaggcggtccattaccttttcgtgggacattttaagcccacagtttatgtacgggagtcgcgctttcatcaagcaccctgcggacatcccagattactacaaacagtctttccccgaggggttcaagtgggagcgcgtgatgaacttcgaggatggcggagccgtgacggtcacccaagatacctctttggaggacggtacgttgatctacaaagtgaaattgcgtggcacgaattttccacctgatgggcctgtcatgcagaaaaagacaatgggatgggaagcttccacggagcgcctttacccagaggacggtgttcttaaaggggatatcaaaatggcgctgcgtcttaaagatggaggccgctacctggcggacttcaagactacttacaaggccaaaaaaccagtgcagatgccgggtgcgtacaatgtagatcgtaaattagatattacaagtcacaatgaagattacacggtcgtagagcagtatgagcgcagtgaggggcgtcactctacgggcggtatggacgagttatacaagtaa	*CIDAR MoClo Extension, Volume I
CymRAM	atgagcccgaaacgtcgtacccaggcagaacgtgcaatggaaacccagggtaaactgattgcagcagcactgggtgttctgcgtgaaaaaggttatgcaggttttcgtattgcagatgttccgggtgcagccggtgttagccgtggtgcacagagccatcattttccgaccaaactggaactgctgctggcaacctttgaatggctgtatgagcagattaccgaacgtagccgtgcacgtctggcaaaactgaaaccggaagatgatgttattcagcagatgctggatgatgcagcagaatttttctggatgatgattttagcatcggcctggatctgattgttgcagcagatcgtgatccggcactgcgtgaaggtattcagcgtaccgttgaacgtaatcgttttgttgttgaagatatgtggctgggtgtgctggtgagccgtggtctgagccgtgatgatgccgaagatattctgtggctgatttttaacagcgttcgtggtctggtagttcgtagcctgtggcagaaagataaagaacgttttgaacgtgtgcgtaatagcaccctggaaattgcacgtgaacgttatgcaaaattcaaacgttga	Meyers *et al.* 2019
VanRAM	atggacatgcctcgtattaaaccgggtcagcgtgttatgatggcactgcgtaaaatgattgcaagcggtgaaatcaaaagtggtgaacgtattgcagaaattccgaccgcagcagcactgggtgttagccgtatgccggttcgtatcgcactgcgttcactggaacaagaaggtctggttgttcgtctgggtgcacgtggttatgcagcccgtggtgttagcagcgatcagattcgtgatgcaattgaagttcgtggtgttctggaaggttttgcagcacgtcgtctggcagaacgtggtatgaccgcagaaacccatgcacgttttgttgtactgattgcagaaggtgaagcactgtttgcagccggtcgcctgaatggtgaagatctggatcgttatgccgcatataatcaggcatttcatgataccctggttagcgcagcaggtaatggtgcagttgaaagcgcactggcacgtaatggttttgaaccgtttgcagcagccggtgcactggccctggatctgatggacctgtctgccgaatatgaacatctgctggcagcacatcgtcagcatcaggcagttctggatgcagttagctgtggtgatgccgaaggtgcagaacgtattatgcgtgatcatgcactggcagcaattcgtaatgcaaaagtttttgaagcagcagcaagcgcaggcgcaccgctgggtgcagcatggtcaattcgtgcagattga	Meyers *et al.* 2019
LuxR	atgaaaaacataaatgccgacgacacatacagaataattaataaaattaaagcttgtagaagcaataatgatattaatcaatgcttatctgatatgactaaaatggtacattgtgaatattatttactcgcgatcatttatcctcattctatggttaaatctgatatttcaatcctagataattaccctaaaaaatggaggcaatattatgatgacgctaatttaataaaatatgatcctatagtagattattctaactccaatcattcaccaattaattggaatatatttgaaaacaatgctgtaaataaaaaatctccaaatgtaattaaagaagcgaaaacatcaggtcttatcactgggtttagtttccctattcatacggctaacaatggcttcggaatgcttagttttgcacattcagaaaaagacaactatatagatagtttatttttacatgcgtgtatgaacataccattaattgttccttctctagttgataattatcgaaaaataaatatagcaaataataaatcaaacaacgatttaaccaaaagagaaaaagaatgtttagcgtgggcatgcgaaggaaaaagctcttgggatatttcaaaaatattaggttgcagtgagcgtactgtcactttccatttaaccaatgcgcaaatgaaactcaatacaacaaaccgctgccaaagtatttctaaagcaattttaacaggagcaattgattgcccatactttaaaaattga	Meyers *et al.* 2019

RepAv7	atgtctgaattagttgttttcaaagcaaatgaactagcgattagtcgctatgacttaacggagcatgaaaccaagctaattttatgctgtgtggcactactcaaccccacgattgaaaaccctacaaggaaagaacggacggtatcgttcacttataaccaatacgctcagatgatgaacatcagtagggaaaatgcttatggtgtattagctaaagcaaccagagagctgatgacgagaactgtggaaatcaggaatcctttggttaaaggctttgagattttccagtggacagactatgccaagttctcaagcgaaaaattagaattagtttttagtgaagagatattgccttatcttttccagttaaaaaaattcataaaatataatctggaacatgttaagtcttttgaaaacaaatactctatgaggatttatgagtggtattaaaagaactaacacaaaagaaaactcacaaggcaaatatagagattagccttgatgaatttaagttcatgttaatgcttggaaataactaccatgagtttaaaaggcttaaccaatgggttttgaaaccaataagtaaagatttaaacacttacagcaatatgaaattggtggttgataagcgaggccgcccgactgatacgttgattttccaagttgaactagatagacaaatggatctcgtaaccgaacttgagaacaaccagataaaaaatgaatggtgacaaaataccaacaaccattacatcagattcctacctacgtaacggactaagaaaaacactacacgatgctttaactgcaaaaattcagctcaccagttttgaggcaaaatttttgagtgacatgcaaagtaagcatgatctcaatggttcgttctcatggctcacgcaaaaacaacgaaccacactagagaacatactggctaaatacggaaggatctga	Segall-Shapiro *et al.* 2018
AmtR	atggcaggcgcagttggtcgtccgcgtcgtagtgcaccgcgtcgtgcaggtaaaaatccgcgtgaagaaattctggatgcaagcgcagaactgtttacccgtcagggttttgcaaccaccagtacccatcagattgcagatgcagttggtattcgtcaggcaagcctgtattatcattttccgagcaaaaccgaaatctttctgaccctgctgaaaagcaccgttgaaccgagcaccgttctggcagaagatctgagcaccctggatgcaggtccggaaatgcgtctgtgggcaattgttgcaagcgaagttcgtctgctgctgagcaccaaatggaatgttggtcgtctgtatcagctgccgattgttggtagcgaagaatttgcagaatatcatagccagcgtgaagcactgaccaatgtttttcgtgatctggcaaccgaaattgttggtgatgatccgcgtgcagaactgccgtttcatattaccatgagcgttattgaaatgcgtcgcaatgatggtaaaattccgagtccgctgagcgcagatagcctgccggaaaccgcaattatgctggcagatgcaagcctggcagttctgggtgcaccgctgcctgcagatcgtgttgaaaaaaccctggaactgattaaacaggcagatgcaaaataa	Nielsen *et al.* 2016
TetR	atgtccagattagataaaagtaaagtgattaacagcgcattagagctgcttaatgaggtcggaatcgaaggtttaacaacccgtaaactcgcccagaagctaggtgtagagcagcctacattgtattggcatgtaaaaaataagcgggctttgctcgacgccttagccattgagatgttagataggcaccatactcacttttgccctttagaaggggaaagctggcaagattttttacgtaataacgctaaaagttttagatgtgctttactaagtcatcgcgatggagcaaaagtacatttaggtacacggcctacagaaaaacagtatgaaactctcgaaaatcaattagccttttatgccaacaaggtttttcactagagaatgcattatatgcactcagcgctgtggggcattttactttaggttgcgtattggaagatcaagagcatcaagtcgctaaagaagaaagggaaacacctactactgatagtatgccgccattattacgacaagctatcgaattatttgatcaccaaggtgcagagccagccttcttattcggccttgaattgatcatatgcggattagaaaaacaacttaaatgtgaaagtgggtcctaa	Lugagne *et al.* 2017
LacI	atggtgaatgtgaaaccagtaacgttatacgatgtcgcagagtatgccggtgtctcttatcagaccgtttcccgcgtggtgaaccaggccagccacgtttctgcgaaaacgcgggaaaaagtggaagcggcgatggcggagctgaattacattcccaaccgcgtggcacaacaactggcgggcaaacagtcgttgctgattggcgttgccacctccagtctggccctgcacgcgccgtcgcaaattgtcgcggcgattaaatctcgcgccgatcaactgggtgccagcgtggtggtgtcgatggtagaacgaagcggcgtcgaagcctgtaaagcggcggtgcacaatcttctcgcgcaacgcgtcagtgggctgatcattaactatccgctggatgaccaggatgccattgctgtggaagctgcctgcactaatgttccggcgttatttcttgatgtctctgaccagacacccatcaacagtattattttctcccatgaagatggtacgcgactgggcgtggagcatctggtcgcattgggtcaccagcaaatcgcgctgttagcgggcccattaagttctgtctcggcgcgtctgcgtctggctggctggcataaatatctcactcgcaatcaaattcagccgatagcggaacgggaaggcgactggagtgccatgtccggttttcaacaaaccatgcaaatgctgaatgagggcatcgttcccactgcgatgctggttgccaacgatcagatggcgctgggcgcaatgcgcgccattaccgagtccgggctgcgcgttggtgcggatatctcggtagtgggatacgacgataccgaagatagctcatgttatatcccgccgttaaccaccatcaaacaggattttcgcctgctggggcaaaccagcgtggaccgcttgctgcaactctctcagggccaggcggtgaagggcaatcagctgttgcccgtctcactggtgaaaagaaaaaccaccctggcgcccaatacgcaaaccgcctctccccgcgcgttggccgattcattaatgcagctggcacgacaggtttcccgactggaaagcgggcagtaa	Lugagne *et al.* 2017
eGFP	atgagcaagggcgaggagctgttcaccggggtggtgcccatcctggtcgagctggacggcgacgtaaacggccacaagttcagcgtgtccggcgagggcgagggcgatgccacctacggcaagctgaccctgaagttcatctgcaccaccggcaagctgcccgtgccctggcccaccctcgtgaccaccctgacctacggcgtgcagtgcttcagccgctaccccgaccacatgaagcagcacgacttcttcaagtccgccatgcccgaaggctacgtccaggagcgcaccatcttcttcaaggacgacggcaactacaagacccgcgccgaggtgaagttcgagggcgacaccctggtgaaccgcatcgagctgaagggcatcgacttcaaggaggacggcaacatcctggggcacaagctggagtacaactacaacagccacaacgtctatatcatggccgacaagcagaagaacggcatcaaggtgaacttcaagatccgccacaacatcgaggacggcagcgtgcagctcgccgaccactaccagcagaacacccccatcggcgacggccccgtgctgctgcccgacaaccactacct	Lugagne *et al.* 2017

	gagcacccagtccaagctgagcaaagaccccaacgagaagcgcgatcacatggtcctgctggagttcgtgaccgccgccgggatc<br>actctcggcatggacgagctgtacaagtaa	
mKate2	atgtctgagctgattaaggagaacatgcacatgaagctgtacatggagggcaccgtgaacaaccaccacttcaagtgcacatccgag<br>ggcgaaggcaagccctacgagggcacccagaccatgagaatcaaggccgtcgagggcggccctctcccctcgccttcgacatcct<br>ggctaccagcttcatgtacggcagcaaaaccttcatcaaccacacccagggcatccccgacttctttaagcagtccttccctgagggc<br>ttcacatgggagagagtcaccacatacgaagatgggggcgtgctgaccgctacccaggacaccagcctccaggacggctgcctcat<br>ctacaacgtcaagatcagaggggtgaacttcccatccaacggccctgtgatgcagaagaaaacactcggctgggaggcctccaccg<br>agacgctgtaccccgctgacggcggcctggaaggcagagccgacatggccctgaagctcgtgggcgggggccacctgatctgcaa<br>cttaaagaccacatacagatccaagaaacccgctaagaacctcaagatgcccggcgtctactatgtggacagacgactggaaagaat<br>caaggaggccgacaaagaaacctacgtcgagcagcacgaggtggctgtggccagatactgcgacctccctagcaaactggggcac<br>agataa	Lugagne *et al.* 2017
ASV tag	aggcctgcagcaaacgacgaaaactacgctgcatcagtttga	Anderson *et al.* 1998
AAV tag	aggcctgcagcaaacgacgaaaactacgctgcagcagtttga	Anderson *et al.* 1998
LVA tag	aggcctgcagcaaacgacgaaaactacgctttagtagcttga	Anderson *et al.* 1998

*Keys: *CIDAR MoClo Extension, Volume I was a gift from Richard Murray (Addgene kit #1000000161).*

Supplementary Table 6: Transcription terminator parts

Part name	Part sequence	Sequence Source
L3S3P21*	ccaattattgaaggcctccctaacggggggcctttttttgtttctggtctgcc	Chen *et al.* 2013
L3S2P21	ctcggtaccaaattccagaaaagaggcctcccgaaaggggggccttttttcgttttggtcc	Chen *et al.* 2013
L3S2P55	ctcggtaccaaagacgaacaataagacgctgaaaagcgtcttttttcgttttggtcc	Chen *et al.* 2013
ECK120029600	ttcagccaaaaaacttaagaccgccggtcttgtccactaccttgcagtaatgcggtggacaggatcggcggttttcttttctcttctcaa	Chen *et al.* 2013
B0015	ggtccaggcatcaaataaaacgaaaggctcagtcgaaagactgggcctttcgttttatctgttgtttgtcggtgaacgctctctactagagtcacactggctcaccttcgggtgggcctttctgcgtttatagctt	iGEM
B1002	cgcaaaaaaccccgcttcggcggggttttttcgc	iGEM
lambda-tL3	ctactggtattggcacaaacctgattccaatttgagcaaggctatgtgccatctcgatactcgttcttaactcaacagaagatgctttgtgcatacagcccctcgtttattatttatctcctcagccagccgctgtgctttcagtggatttcggataacagaaaggccgggaaatacccagcctcgctttgtaacggagtagagacgaaagtgattgcgcctacccggatattatcgtgaggatgcg	St-Pierre *et al.* 2013
tonB	aaaacctcgcgccttacctgttgagtaatagtcaaaagcctccggtcggaggcttttgactttctgcttactgaatttcggtggtgccgttaa	St-Pierre *et al.* 2013
IOT	taattggtaacgaatcagacaattgacggctcgagggagtagcatagggtttgcagaatccctgcttcgtccatttgacaggcacattatgcatcgatgataagctgtcaaacatgagcagatcctctacgccggacgcatcgtggccggcatcaccggcgccacaggtgcggttgctggcgcctatatcgccgacatcaccgatggggaagatcgggctcgccacttcgggctcatgagcaaatattttatctg	Meyers *et al.* 2019
apFAB815	tcggtcagtttcacctgatttacgtaaaaacccgcttcggcgggtttttgcttttggaggggcagaaagatgaatgactgtc	Lugagne *et al.* 2017
apFAB816	tagagatcaagccttaacgaactaagacccccgcaccgaaaggtccgggggttttttttgaccttaaaaacataaccgaggagcagaca	Lugagne *et al.* 2017

*Keys: *GGTCTC to GGTCTG alteration made to make BsaI safe.*

Supplementary Table 7: Antibiotic resistance cassettes

Part name	Part sequence	Sequence Source
AmpR	cgcggaacccctatttgtttatttttctaaatacattcaaatatgtatccgctcatgagacaataaccctgataaatgcttcaataatattgaaaaaggaagagtatgagtattcaacatttccgtgtcgcccttattcccttttttgcggcattttgccttcctgtttttgctcacccagaaacgctggtgaaagtaaaagatgctgaagatcagttgggtgcacgagtgggttatatcgaactggatctcaacagcggtaagatccttgagagttttcgccccgaagaacgttttccaatgatgagcacttttaaagttctgctatgtggcgcggtattatcccgtattgacgccgggcaagagcaactcggtcgccgcatacactattctcagaatgacttggttgagtactcaccagtcacagaaaagcatcttacggatggcatgacagtaagagaattatgcagtgctgccataaccatgagtgataacactgcggccaacttacttctgacaacgatcggaggaccgaaggagctaaccgcttttttgcacaacatgggggatcatgtaactcgccttgatcgttgggaaccggagctgaatgaagccataccaaacgacgagcgtgacaccacgatgcctgtagcaatggcaacaacgttgcgcaaactattaactggcgaactacttactctagcttcccggcaacaattaatagactggatggaggcggataaagttgcaggaccacttctgcgctcggcccttccggctggctggtttattgctgataaatctggagccggtgagcgtggttctcgcggtatcattgcagcactggggccagatggtaagccctcccgtatcgtagttatctacacgacggggagtcaggcaactatggatgaacgaaatagacagatcgctgagataggtgcctcactgattaagcattggtaactgtcagaccaagtttactcatatatactttagattgatttaaaacttcatttttaatttaaaaggatctaggtgaagatcctttttgataatcggtacg	Segall-Shapiro *et al.* 2018
KanR	ttgtgtctcaaaatctctgatgtacattgcacaagataaaaatatatcatcatgaacaataaaactgtctgcttacataaacagtaatacaaggggtgttatgagccatattcaacgggaaacgtcttgctccaggccgcgattaaattccaacatggatgctgatttatatgggtataaatgggctcgcgataatgtcgggcaatcaggtgcgacaatctatcgattgtatgggaagcccgatgcgccagagttgtttctgaaacatggcaaaggtagcgttgccaatgatgttacagatgagatggtcagactaaactggctgacggaatttatgcctcttccgaccatcaagcattttatccgtactcctgatgatgcatggttactcaccactgcgatccccgggaaaacagcattccaggtattagaagaatatcctgattcaggtgaaaatattgttgatgcgctggcagtgttcctgcgccggttgcattcgattcctgtttgtaattgtccttttaacagcgatcgcgtatttcgtctcgctcaggcgcaatcacgaatgaataacggtttggttgatgcgagtgattttgatgacgagcgtaatggctggcctgttgaacaagtctggaaagaaatgcataagcttttgccattctcaccggattcagtcgtcactcatggtgatttctcacttgataaccttatttttgacgaggggaaattaataggttgtattgatgttggacgagtcggaatcgcagaccgataccaggatcttgccatcctatggaactgcctcggtgagttttctccttcattacagaaacggctttttcaaaaatatggtattgataatcctgatatgaataaattgcagtttcatttgatgctcgatgagtttttctaaggtaactgtcagaccaagtttactcatatatactttagattgatttttcgttcc	Meyers *et al.* 2019
CamR	tgatcggcacgtaagaggttccaactttcaccataatgaaataagatcactaccgggcgtattttttgagttatcgagattttcaggagctaaggaagctaaaatggagaaaaaaatcactggatataccaccgttgatatatcccaatggcatcgtaaagaacattttgaggcatttcagtcagttgctcaatgtacctataaccagaccgttcagctggatattacggcctttttaaagaccgtaaagaaaaataagcacaagttttatccggcctttattcacattcttgcccgcctgatgaatgctcatccggaattccgtatggcaatgaaagacggtgagctggtgatatgggatagtgttcacccttgttacaccgttttccatgagcaaactgaaacgttttcatcgctctggagtgaataccacgacgatttccggcagtttctacacatatattcgcaagatgtggcgtgttacggtgaaaacctggcctatttccctaaagggtttattgagaatatgtttttcgtctcagccaatccctgggtgagtttcaccagttttgatttaaacgtggccaatatggacaacttcttcgcccccgttttcaccatgggcaaatattatacgcaaggcgacaaggtgctgatgccgctggcgattcaggttcatcatgccgtttgtgatggcttccatgtcggcagaatgcttaatgaattacaacagtactgcgatgagtggcagggcggggcgtaatttgatatcgagctcgcttggactcctgttgatagatccagtaatgacctcagaactccatctggatttgttcagaacgctcggttgccgccgggcgtttttttattggtgagaatccaa	St-Pierre *et al.* 2013

Keys: Green indicates predicted promoter-RBS; Blue indicates CDS; underlined indicates predicted terminator region.

Supplementary Table 8: Origins of replication

Part name	Part sequence	Sequence Source
pMB1/pUC19	ttgagatcctttttttctgcgcgtaatctgctgcttgcaaacaaaaaaaccaccgctaccagcggtggtttgtttgccggatcaagagctaccaactctttttccgaaggtaactggcttcagcagagcgcagataccaaatactgttcttctagtgtagccgtagttaggccaccacttcaagaactctgtagcaccgcctacatacctcgctctgctaatcctgttaccagtggctgctgccagtggcgataagtcgtgtcttaccgggttggactcaagacgatagttaccggataaggcgcagcggtcgggctgaacggggggttcgtgcacacagcccagcttggagcgaacgacctacaccgaactgagatacctacagcgtgagctatgagaaagcgccacgcttcccgaagggagaaaggcggacaggtatccggtaagcggcagggtcggaacaggagagcgcacgagggagcttccagggggaaacgcctggtatctttatagtcctgtcgggtttcgccacctctgacttgagcgtcgatttttgtgatgctcgtcaggggggcggagcctgtggaaa	Iverson *et al.* 2016
ColE1	tatcagctcactcaaaggcggtaattcatgaccaaaatcccttaacgtgagttttcgttccactgagcgtcagaccccgtagaaaagatcaaaggatcttcttgagatcctttttttctgcgcgtaatctgctgcttgcaaacaaaaaaaccaccgctaccagcggtggtttgtttgccggatcaagagctaccaactctttttccgaaggtaactggcttcagcagagcgcagataccaaatactgtccttctagtgtagccgtagttaggccaccacttcaagaactctgtagcaccgcctacatacctcgctctgctaatcctgttaccagtggctgctgccagtggcgataagtcgtgtcttaccgggttggactcaagacgatagttaccggataaggcgcagcggtcgggctgaacggggggttcgtgcacacagcccagcttggagcgaacgacctacaccgaactgagatacctacagcgtgagctatgagaaagcgccacgcttcccgaagggagaaaggcggacaggtatccggtaagcggcagggtcggaacaggagagcgcacgagggagcttccagggggaaacgcctggtatctttatagtcctgtcgggtttcgccacctctgacttgagcgtcgatttttgtgatgctcgtcaggggggcggagcctatggaaaaacgccagcaacgcggcctttttacggttcctggccttttgctggccttttgctcacatgttctttcctgcgttatcccctgattctgtggataaccgtcgattat	Segall-Shapiro *et al.* 2018
p15A	ttgagatcgttttggtctgcgcgtaatctcttgctctgaaaacgaaaaaaccgccttgcagggcggtttttcgaaggttctctgagctaccaactctttgaaccgaggtaactggcttggaggagcgcagtcaccaaaacttgtcctttcagtttagccttaaccggcgcatgacttcaagactaactcctctaaatcaattaccagtggctgctgccagtggtgcttttgcatgtctttccgggttggactcaagacgatagttaccggataaggcgcagcggtcggactgaacggggggttcgtgcatacagtccagcttggagcgaactgcctacccggaactgagtgtcaggcgtggaatgagacaaacgcggccataacagcggaatgacaccggtaaaccgaaaggcaggaacaggagagcgcacgagggagccgccagggggaaacgcctggtatctttatagtcctgtcgggtttcgccaccactgatttgagcgtcagatttcgtgatgcttgtcaggggggcggagcctatggaaa	Meyers *et al.* 2019
R6K	gatctgaagatcagcagttcaacctgttgatagtacgtactaagctctcatgtttcacgtactaagctctcatgtttaacgtactaagctctcatgtttaacgaactaaaccctcatggctaacgtactaagctctcatggctaacgtactaagctctcatgtttcacgtactaagctctcatgtttgaacaataaaattaatataaatcagcaacttaaatagcctctaaggttttaagttttataagaaaaaaaagaatatataaggcttttaaagcttttaaggtttaacggttgtggacaacaagccagggatgtaacgcactgagaagcccttagagcctctcaaagcaattttgagtgacacaggaacacttaacggctgaca	St-Pierre *et al.* 2013
pSC101-WT	ccgaagtggtcagactggaaaatcagagggcaggaactgctgaacagcaaaaagtcagatagcaccacatagcagacccgccataaaacgccctgagaagcccgtgacgggcttttcttgtattatgggtagtttccttgcatgaatccataaaaggcgcctgtagtgccatttacccccattcactgccagagccgtgagcgcagcgaactgaatgtcacgaaaaagacagcgactcaggtgcctgatggtcggagacaaaaggaatattcagcgatttgcccgagcttgcgagggtgctacttaagcctttagggttttaaggtctgttttgtagaggagcaaacagcgtttgcgacatccttttgtaatactgcggaactgactaaagtagtgagttatacacagggctgggatctattctttttatctttttttattctttctttattctataaattataaccacttgaatataaacaaaaaaaacacacaaaggtctagcggaatttacagagggtctagcagaatttacaagttttccagcaaaggtctagcagaatttacagatacccacaactcaaaggaaaaggactagtaattatcattgactagcccatctcaattggtatagtgattaaaatcacctagaccaattgagatgtatgtctgaattagttgttttcaaagcaaatgaactagcgattagtcgctatgacttaacggagcatgaaaccaagctaattttatgctgtgtggcactactcaaccccacgattgaaaaccctacaaggaaagaacggacggtatcgttcacttataaccaatacgctcagatgatgaacatcagtagggaaaatgcttatggtgtattagctaaagcaaccagagagctgatgacgagaactgtggaaatcaggaatcctttggttaaaggctttgagattttccagtggacaaactatgccaagttctcaagcgaaaaattagaattagtttttagtgaagagatattgccttatcttttccagttaaaaaaattcataaaatataatctggaacatgttaagtcttttgaaaacaaatactctatgaggatttatgagtggttattaaaagaactaacacaaaagaaaactcacaaggcaaatatagagattagccttgatgaatttaagttcatgttaatgcttgaaaataactaccatgagtttaaaaggcttaaccaatgggttttgaaaccaataagtaaagatttaaacacttacagcaatatgaaattggtggttgataagcgaggccgcccgactgatacgttgattttccaagttgaactagatagacaaatggatctcgtaaccgaacttgagaacaaccagataaaaatgaatggtgacaaaataccaacaaccattacatca	Segall-Shapiro *et al.* 2018

	gattcctacctacataacggactaagaaaaacactacacgatgctttaactgcaaaaattcagctcaccagttttgaggcaaaattttg<br>agtgacatgcaaagtaagtatgatctcaatggttcgttctcatggctcacgcaaaaacaacgaaccacactagagaacatactggcta<br>aatacggaaggatctgaggttcttatggctcttgtatctatcagtgaagcatcaagactaacaaaca	
TULIP	ccgaagtggtcagactggaaaatcagagggcaggaactgctgaacagcaaaaagtcagatagcaccacatagcagacccgccata<br>aaacgccctgagaagcccgtgacgggcttttcttgtattatgggtagtttccttgcatgaatccataaaaggcgcctgtagtgccattt<br>acccccattcactgccagagccgtgagcgcagcgaactgaatgtcacgaaaaagacagcgactcaggtgcctgatggtcggagaca<br>aaaggaatattcagcgatttgcccgagcttgcgagggtgctacttaagcctttagggttttaaggtctgttttgtagaggagcaaaca<br>gcgtttgcgacatccttttgtaatactgcggaactgactaaagtagtgagttatacacagggctgggatctattctttttatctttttttat<br>tctttctttattctataaattataaccacttgaatataaacaaaaaaaacacacaaaggtctagcggaatttacagagggtctagcagaa<br>tttacaagttttccagcaaaggtctagcagaatttacagatacccacaactcaaaggaaaaggactagtaattatcattgactagccca<br>tctcaattgggttttatcttgtgcaatgtacatcagagattttgagacacaaccaattattgaaggcctccctaacgggggcctttttt<br>gtttctggtctgccgcttaacgatcgttggctgaacaaacagacaatctggtctgtttgtattatggaaaatttttctgtataatagattc<br>aacaaacagacaatctggtctgtttgtattatagctgtcacggtagactgtcgccggatgtgtatccgacctgacgatggcccaaaag<br>ggccgaaacagtcctctacaaataattttgtttaatcacactatgctttactagatgtctgaattagttgttttcaaagcaaatgaactag<br>cgattagtcgctatgacttaacggagcatgaaaccaagctaattttatgctgtgtggcactactcaaccccacgattgaaaaccctaca<br>aggaaagaacggacggtatcgttcacttataaccaatacgctcagatgatgaacatcagtagggaaaatgcttatggtgtattagcta<br>aagcaaccagagagctgatgacgagaactgtggaaatcaggaatcctttggttaaaggctttgagattttccagtggacagactatg<br>ccaagttctcaagcgaaaaattagaattagtttttagtgaagagatattgccttatcttttccagttaaaaaaattcataaaatataatctg<br>gaacatgttaagtcttttgaaaacaaatactctatgaggatttatgagtggttattaaaagaactaacacaaaagaaaactcacaaggc<br>aaatatagagattagccttgatgaatttaagttcatgttaatgcttggaaataactaccatgagtttaaaaggcttaaccaatgggttttg<br>aaaccaataagtaaagatttaaacacttacagcaatatgaaattggtggttgataagcgaggccgcccgactgatacgttgattttcca<br>agttgaactagatagacaaatggatctcgtaaccgaacttgagaacaaccagataaaaatgaatggtgacaaaataccaacaaccatt<br>acatcagattcctacctacgtaacggactaagaaaaacactacacgatgctttaactgcaaaaattcagctcaccagttttgaggcaaa<br>atttttgagtgacatgcaaagtaagcatgatctcaatggttcgttctcatggctcacgcaaaaacaacgaaccacactagagaacatac<br>tggctaaatacggaaggatcaggcctgcagcaaacgacgaaaactacgctgcagcagtttgataacaaagaggagaaatactagat<br>gagcccgaaacgtcgtacccaggcagaacgtgcaatggaaacccagggtaaactgattgcagcagcactgggtgttctgcgtgaaa<br>aaggttatgcaggttttcgtattgcagatgttccgggtgcagccggtgttagccgtggtgcacagagccatcattttccgaccaaact<br>ggaactgctgctggcaacctttgaatggctgtatgagcagattaccgaacgtagccgtgcacgtctggcaaaactgaaaccggaag<br>atgatgttattcagcagatgctggatgatgcagcagaatttttctggatgatgattttagcatcggcctggatctgattgttgcagcag<br>atcgtgatccggcactgcgtgaaggtattcagcgtaccgttgaacgtaatcgttttgttgttgaagatatgtggctgggtgtgctggtg<br>agccgtggtctgagccgtgatgatgccgaagatattctgtggctgatttttaacagcgttcgtggtctggtagttcgtagcctgtggca<br>gaaagataaagaacgttttgaacgtgtgcgtaatagcaccctggaaattgcacgtgaacgttatgcaaaattcaaacgttgataactc<br>ggtaccaaattccagaaaagaggcctcccgaaagggggcctttttcgttttggtccggttcttatggctcttgtatctatcagtgaa<br>gcatcaagactaacaaaca	This work

Keys: Green indicates Ori site; Blue indicates RepA CDS; Purple indicates wildtype par (partition) domain; Red underlined indicates PCymRC promoter and Red indicates CymRAM CDS.

Supplementary Table 9: Key primers used in the paper

Primer #	Alias	Sequence	Purpose
SJ061Fv1	RepAv7_N7_F	ccattgggtctcgtcacacnnnnnnntactagatgtctgaattagttgttttcaaagc	Library Prep
SJ070F	RepAv7_N3_F	ccattgggtctcgtcacacngnagnttactagatgtctgaattagttgttttcaaagc	Library Prep
SJ010R	RepAv7_R	ccattgggtctcggttatcagatccttccgtatttagcc	Library Prep
SJ008F	LibBase_C1_F	ccattgggtctcctaacggaagagagtcaattcaggg	Library Prep
SJ071Fv1	LibBase_N3_F	ccattgggtctcctaactcacacngnagnttactagatgagcccgaaacgtcgtac	Library Prep
SJ071Fv2	LibBase_B0034_F	ccattgggtctcctaacaaagaggagaaatactagatgagcccgaaacgtcgtac	Library Prep
SJ071Fv3	LibBase_B0032_F	ccattgggtctcctaactcacacaggaaagtactagatgagcccgaaacgtcgtac	Library Prep
SJ009R	LibBase_R	ccattgggtctcggtgattaaacaaaattatttgtagaggactg	Library Prep
SJ011F	DXS2_F	aaacgaatgtcgccagatgc	qPCR
SJ011R	DXS2_R	acgcttcacaatgcctttgc	qPCR
SJ013F	Amp1_F	tcgtcgtttggtatggcttc	qPCR
SJ013R	Amp1_R	taacactgcggccaacttac	qPCR
SJ172F	KanR_38nt	ggcttaccatccagcgccaccatccagtgcaggagctcggaacgaaaaatcaatctaaag	Lambda-RED
SJ172R	KanR_38nt	gatagcgcccggaagagagtcaattcagggtggtgaatttgtgtctcaaaatctctgatg	Lambda-RED
SJ173F	KanR_80nt	ccttgtggagcgacatccagaggcacttcaccgcttgccagcggcttaccatccagcgc	Lambda-RED
SJ173R	KanR_80nt	ttgacaccatcgaatggcgcaaaacctttcgcggtatggcatgatagcgcccggaagag	Lambda-RED

Keys: ggtctcg BsaI recognition site and nnnn 4 bp sticky end; Orange indicates RBS insert and Orange underlined indicates Shine-Dalgarno sequence; Lavender indicates homology arm sequences that would flank the KanR cassette to be integrated into the E. coli genome, simultaneously knocking-out the lacI gene; Underlined indicates primer binding sequence.

Supplementary Table 10: Guide RNA designs for dCas9 used in the paper

Guide	Target on CymRAM	Sequence	PAM	Source
gRNA#1	+ Strand at 28-47 nt	gaacgtgcaatggaaaccca	ggg	*Benchling
gRNA#2	+ Strand at 18-37 nt	tacccaggcagaacgtgcaa	tgg	*Benchling
gRNA#3	+ Strand at 27-46 nt	agaacgtgcaatggaaaccc	agg	*Benchling
gRNA#4	+ Strand at 52-71 nt	aaactgattgcagcagcact	ggg	*Benchling
gRNA#5	- Strand at 48-67 nt	ctgctgcaatcagtttaccc	tgg	*Benchling
gRNA#6	- Strand at 47-66 nt	tgctgcaatcagtttaccct	ggg	*Benchling
gRNA#7	- Strand at 23-42 nt	ttccattgcacgttctgcct	ggg	*Benchling
gRNA#8	- Strand at 24-43 nt	tttccattgcacgttctgcc	tgg	*Benchling
gRNA#9	Control Non-target	acctgagccttcagcaacgt	n/a	Random

*Keys:*All target guides were designed using the Benchling CRISPR guide design tool (Benchling 2022, retrieved from https://benchling.com), the top 8 designs were selected for characterization. The Control Non-targeting guide RNA was designed by a random nucleotide generator set to return a 20 nt sequence with 50% GC content, this was then BLASTed against TULIP and a K-12 E. coli MG1655 reference genome (GenBank CP032667.1) to confirm there are no targets.*

3 Mathematical model and analysis

Here, we present the mathematical modeling and analysis complementing the experimental data for the single-layer sensor modules in Figures 5–6, the NOT gate in Supplementary Fig. 14, and the toggle switch in Figure 7 and Supplementary Fig. 20, together with the typical range of model parameters.

3.1 Single layer sensor modules and NOT gate

Consider first the sensor module in Figure 5a relying on the activator TF. Let D denote the PCN, so that the total concentration x_T of the activator TF (LuxR) is given by $x_T = \alpha_x D/\lambda_x$, where the production rate constant α_x is proportional to the RBS (B0032 or B0034) strength of the activator, and λ_x denotes the degradation rate constant. Define $\bar{\alpha}_x = \alpha_x/\lambda_x$. Let u, x, and x_u denote the concentration of the inducer (AHL), the TF without the inducer, and activated TF, respectively. The total concentration of the TF is partitioned as $x_T = x + x_u$ with $x = x_T K_u/(K_u + u)$ and $x_u = x_T u/(K_u + u)$ at the steady state, where K_u is the dissociation constant of the TF-inducer binding. The output y of the sensor (mScarI) is given by

$$y = \frac{1}{\lambda_y}\left(\alpha_y D \frac{x_u^n}{K_x^n + x_u^n} + \alpha_{y,0} D\right),$$

where α_y and λ_y are production and degradation rate constants, respectively, $0 < \alpha_{y,0} \ll \alpha_y$ is introduced to capture promoter leakiness, and K_x is the dissociation constant of x_u binding to the promoter (PLuxB) of the reporter protein with Hill coefficient n. With LuxR acting as a dimer (*19*), we obtain that

$$\begin{aligned}\frac{\partial y}{\partial u} &= \frac{1}{\lambda_y}\frac{2\alpha_y \bar{\alpha}_x^2 D^3 K_u K_x^2}{\left(K_u^2 K_x^2 + K_x^2 u^2 + \bar{\alpha}_x^2 D^2 u^2 + 2K_u K_x^2 u\right)^2},\\ \frac{\partial y}{\partial D} &= \frac{1}{\lambda_y}\frac{\bar{\alpha}_x^2 \alpha_y D^2 u^2\left(\bar{\alpha}_x^2 D^2 u^2 + 3K_x^2 K_u^2 + 6K_x^2 K_u u + 3K_x^2 u^2\right)}{\left(\bar{\alpha}_x^2 D^2 u^2 + K_x^2 K_u^2 + K_x^2 u^2 + 2K_x^2 K_u u^2\right)^2} + \alpha_{y,0}.\end{aligned} \tag{1}$$

In case of the repressor-based sensor module from Figure 5b with the inducer u (Van) and using the same notation as above, the output y of the module (mScarI) is given by

$$y = \frac{1}{\lambda_y}\left(\alpha_y D \frac{K_x^n}{K_x^n + x^n} + \alpha_{y,0} D\right), \tag{2}$$

where K_x now denotes the dissociation constant of the repressor (VanRAM) binding to the promoter (PVanCC) of the reporter. With VanR acting as a dimer (*20*), we obtain that

$$\begin{aligned}\frac{\partial y}{\partial u} =& \frac{1}{\lambda_y} \frac{2\alpha_y \bar{\alpha}_x^2 D^3 K_u^2 K_x^2 (K_u + u)}{\left[K_x^2 (K_u + u)^2 + \bar{\alpha}_x^2 D^2 K_u^2\right]^2}, \\ \frac{\partial y}{\partial D} =& \frac{1}{\lambda_y} \frac{\alpha_y K_x^2 (K_u + u)^2 (-\bar{\alpha}_x^2 D^2 K_u^2 + K_x^2 K_u^2 + 2K_x^2 K_u u + K_x^2 u^2)}{(\bar{\alpha}_x^2 D^2 K_u^2 + K_x^2 K_u^2 + K_x^2 u^2 + 2K_x^2 K_u u^2)^2} + \alpha_{y,0}.\end{aligned} \tag{3}$$

Finally, consider the NOT gate in Supplementary Fig. 14. Since its first layer (the Van cassette) is identical to the repressor-based sensor module, the output z (mScarI) is given by

$$z = \frac{1}{\lambda_z}\left(\alpha_z D \frac{K_y^m}{K_y^m + y^m} + \alpha_{z,0} D\right), \tag{4}$$

with y from Supplementary Equation (2), where α_z and λ_z denote the production and degradation rate constant of the output z, respectively, $0 < \alpha_{z,0} \ll \alpha_z$ is introduced to capture promoter leakiness, and K_y is the dissociation rate constant of y binding to the promoter of z (PAmtR).

In case of the activator-based sensor module, from (1) it follows that $\partial y/\partial u > 0$ and $\partial y/\partial D > 0$, hence the output y is expected to monotonically increase with both the inducer u and the PCN D (Supplementary Fig. 104), confirmed experimentally in Figure 5a and Figure 6a. Similarly, for the repressor-based sensor module from (3) we obtain that $\partial y/\partial u > 0$, thus its output is expected to monotonically increase with the inducer u (Supplementary Fig. 104), confirmed experimentally in Figure 5b. As the sensitivity $\partial y/\partial D$ could become negative as $\bar{\alpha}_x D$ increases, we expect that the output of the repressor-based sensor module could display non-monotonic dependence on the PCN D (Supplementary Fig. 104), verified experimentally in Figure 6b. Finally, in case of the NOT gate with AmtR acting as a dimer (*21*) we have $m = 2$,

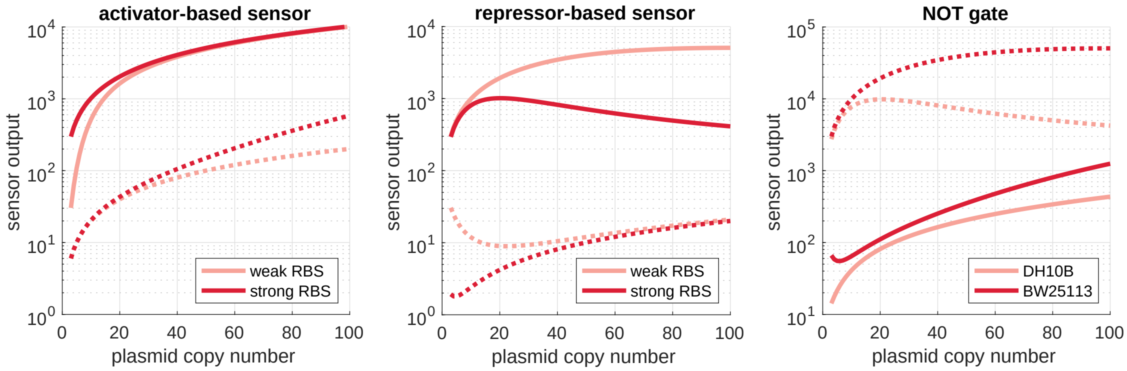

Supplementary Fig. 104. **Simulation results illustrating the dependence of the single-layer sensor modules in Figures 5–6 and the NOT gate in Supplementary Fig. 14 on PCN.** In all plots $K_u = 10$ nM; $u = 0$ μM and $u = 1$ μM in case of dashed and solid lines, respectively, with $D = dD_0$ where $D_0 = 2$ nM corresponds to the concentration of a single molecule in *E. coli* and d denotes the PCN indicated on the x-axis. Simulation parameters for the activator-based sensor module: $\alpha_y = 100$ h^{-1}, $\alpha_{y,0}/\alpha_y = 0.02$, $\lambda_x = \lambda_y = 1$h^{-1}, $K_x = 100$ nM, $\alpha_x = 10$ h^{-1} and $\alpha_x = 200$ h^{-1} for light and dark red, respectively. Simulation parameters for the repressor-based sensor module: $\alpha_y = 100$ h^{-1}, $\alpha_{y,0}/\alpha_y = 0.002$, $\lambda_x = \lambda_y = 1$ h^{-1}, $K_x = 100$ nM, $\alpha_x = 100$ h^{-1} and $\alpha_x = 500$ h^{-1} for light and dark red, respectively. Simulation parameters for the NOT gate module: $\alpha_z = 100$ h^{-1}, $K_x = 100$ nM, $\alpha_{z,0}/\alpha_z = \alpha_{y,0}/\alpha_y = 0.004$, $\lambda_x = \lambda_y = \lambda_z = 1$ h^{-1}, $K_y = 40$ nM, $\alpha_x = 500$ nM, and $\alpha_y = 500$ nM and $\alpha_y = 100$ nM for light and dark red, respectively.

yielding $\partial z/\partial u < 0$ from (4), whereas the sign of $\partial z/\partial D$ can be either positive or negative. Therefore, the output of the NOT gate is expected to monotonically decrease with the inducer u, while its dependence on the PCN may be non-monotonic (Supplementary Fig. 104), both confirmed experimentally in Supplementary Fig. 14.

3.2 Toggle switch

Let y and z denote the concentration of TetR and LacI co-expressed with the reporters eGFP and mKate2, respectively, so that in the absence of the inducers aTc and IPTG the dynamics of the toggle switch are given by

$$\dot{y} = \alpha_{y,0}D_0 + \alpha_y D\frac{K_z^m + \epsilon_y z^m}{K_z^m + z^m} - \lambda_y y, \qquad \dot{z} = \alpha_{z,0}D_0 + \alpha_z D\frac{K_y^n + \epsilon_z y^n}{K_y^n + y^n} - \lambda_z z, \quad (5)$$

where n and m are the Hill coefficients of the repressors y and z, alongside with the production rate constants α_y and α_z, the basal production rate constants $\alpha_{y,0}$ and $\alpha_{z,0}$ from the host genome together with D_0 denoting the concentration of the chromosome, the degradation rate constants λ_y and λ_z, the dissociation rate constants K_y and K_z, and $0 < \epsilon_y, \epsilon_z \ll 1$ are introduced to capture promoter leakiness. Induction via IPTG inhibits repression of the PLacO promoter by LacI (i.e., $K_z \to \infty$), yielding the dynamics

$$\dot{y} = \alpha_{y,0} D_0 + \alpha_y D - \lambda_y y, \qquad \dot{z} = \alpha_{z,0} D_0 + \alpha_z D \frac{K_y^n + \epsilon_z y^n}{K_y^n + y^n} - \lambda_z z \tag{6}$$

with a unique stable equilibrium. Similarly, aTC induction can be taken into account by considering $K_y \to \infty$, resulting in

$$\dot{y} = \alpha_{y,0} D_0 + \alpha_y D \frac{K_z^m + \epsilon_y z^m}{K_z^m + z^m} - \lambda_y y, \qquad \dot{z} = \alpha_{z,0} D_0 + \alpha_z D - \lambda_z z, \tag{7}$$

with a unique stable equilibrium.

In Supplementary Fig. 105 we present simulation data corresponding to the data in Figure 7 and Supplementary Fig. 20 to confirm the experimental observations. For instance, the considerable decrease in post-wash eGFP expression subsequent to IPTG induction in DH10B with chromosomal LacI expression (Supplementary Fig. 105a) is mitigated at low PCN values once this gene is knocked out (Supplementary Fig. 105b).

The simulation results in Supplementary Fig. 105 also highlight that PCN control can be successfully deployed to render monostable toggle switches bistable without further cloning and transformation, for instance, by overcoming basal expression of the repressors from the chromosome. However, this approach does not always ensure a functional toggle switch (*22*): for instance, if the repressors act as monomers (Supplementary Fig. 106a) or in case of unbalanced production rate constants (Supplementary Fig. 106b).

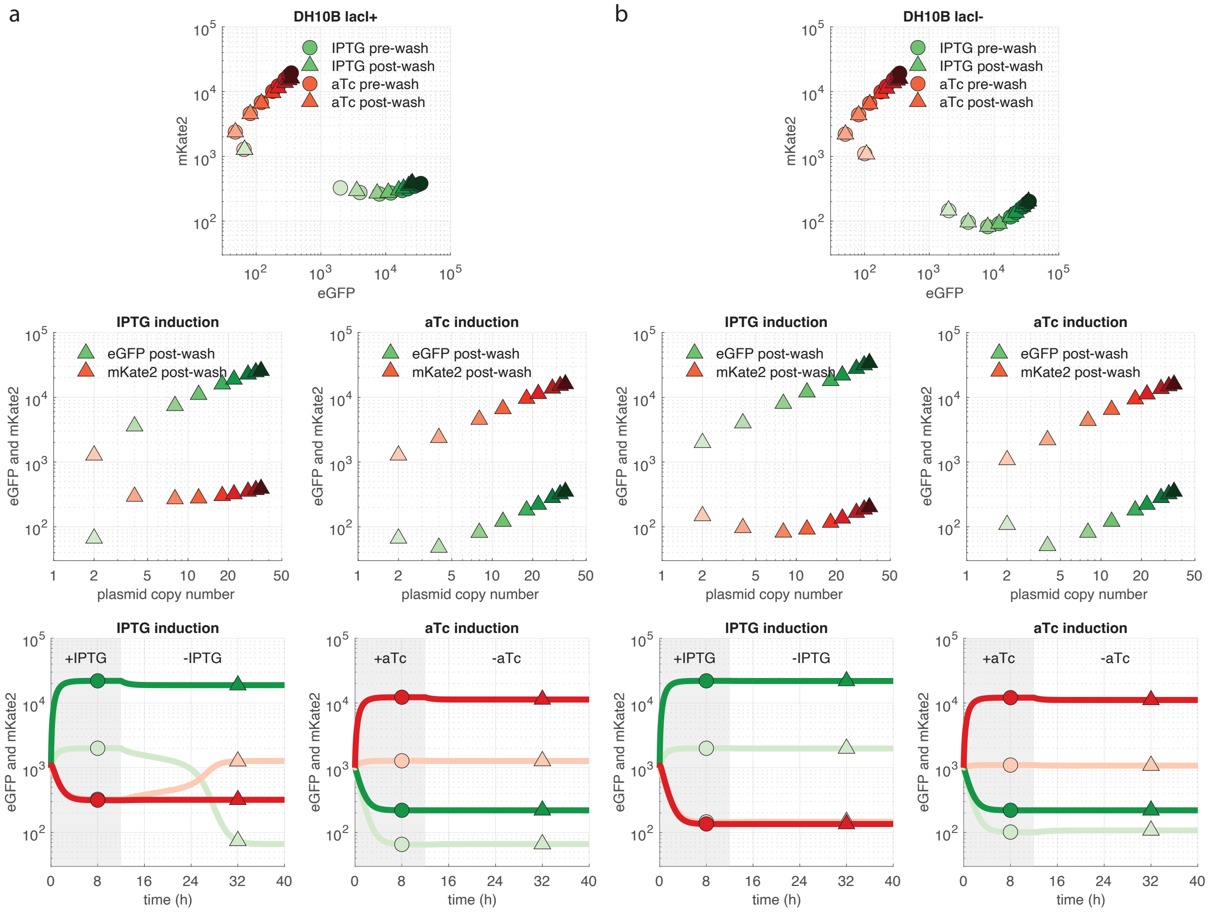

Supplementary Fig. 105. **Behavior of the toggle switch in DH10B cells with and without chromosomal LacI expression.** Darker shades correspond to greater values of PCN (changed via D). Circles denote pre-wash equilibrium values when using Supplementary Equation (6) or Supplementary Equation (7) corresponding to IPTG/aTc induction, respectively. Triangles denote post-wash equilibrium values obtained from Supplementary Equation (5) using the corresponding pre-wash equilibrium as initial point. In all plots $n = 2$, $m = 4$, $\alpha_y = 500\ \mathrm{h}^{-1}$, $\alpha_z = 275\ \mathrm{h}^{-1}$, $\alpha_{z,0} = 0\ \mathrm{h}^{-1}$, $K_y = 500$ nM, $K_z = 750$ nM, $\epsilon_y = \epsilon_z = 0.01$, together with $D = dD_0$ where $D_0 = 2$ nM corresponds to the concentration of a single molecule in *E. coli* and d denotes the PCN with $d = 2, 4, 8, 12, 18, 22, 28, 32, 35$. Samples trajectories for eGFP (green) and mKate2 (red) are shown for $d = 2$ (light) and $d = 22$ (dark). **a** The host genome contains the lacI gene ($\alpha_{y,0} = 90\ \mathrm{h}^{-1}$). **b** The host genome lacks the lacI gene ($\alpha_{y,0} = 0\ \mathrm{h}^{-1}$).

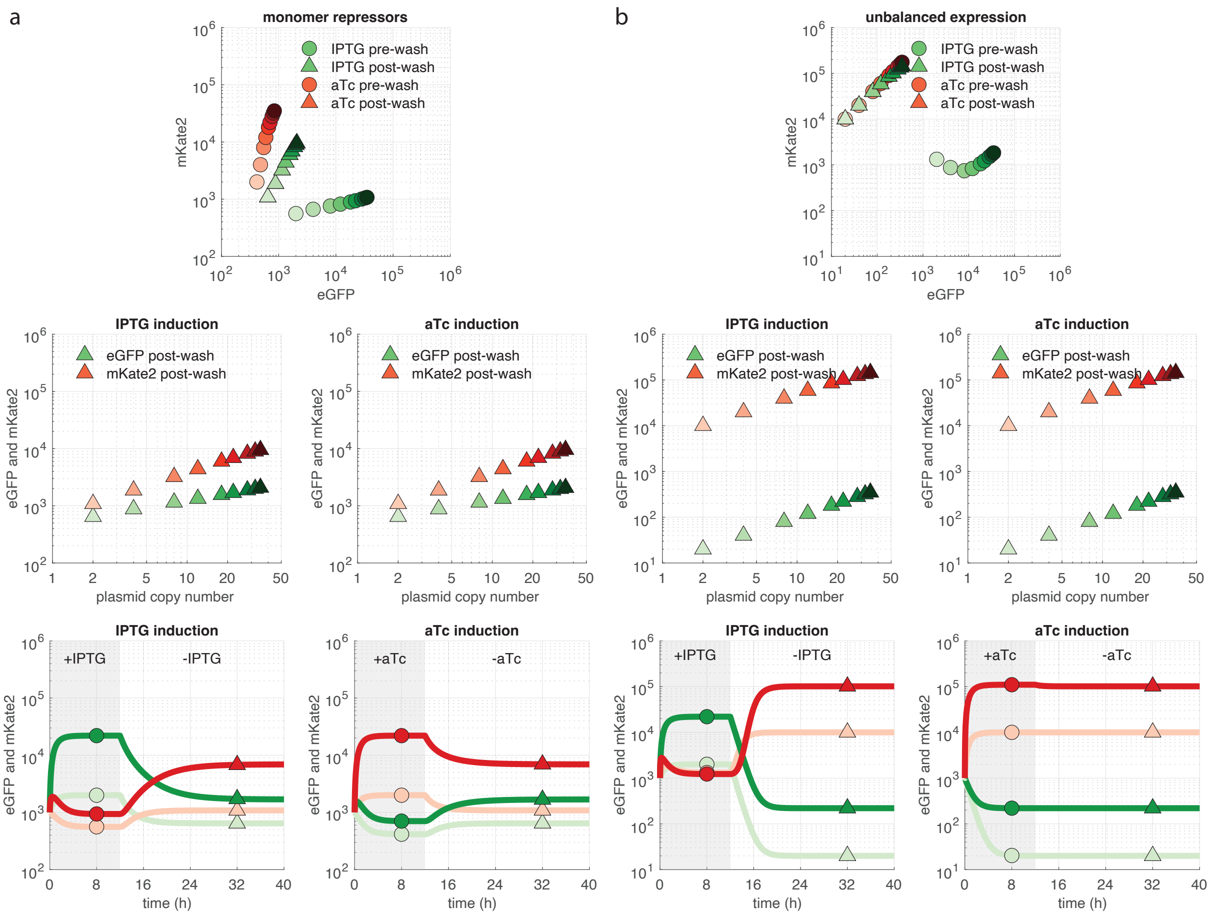

Supplementary Fig. 106. **PCN control is not always sufficient to render monostable toggle switches bistable.** Darker shades correspond to greater values of PCN (changed via D). Circles denote pre-wash equilibrium values when using Supplementary Equation (6) or Supplementary Equation (7) corresponding to IPTG/aTc induction, respectively. Triangles denote post-wash equilibrium values obtained from Supplementary Equation (5) using the corresponding pre-wash equilibrium as initial point. In all plots $\alpha_{y,0} = \alpha_{z,0} = 0\,\mathrm{h}^{-1}$, $K_y = 500\,\mathrm{nM}$, $K_z = 750\,\mathrm{nM}$, $\epsilon_y = \epsilon_z = 0.01$, together with $D = dD_0$ where $D_0 = 2$ nM corresponds to the concentration of a single molecule in *E. coli* and d denotes the PCN with $d = 2, 4, 8, 12, 18, 22, 28, 32, 35$. Samples trajectories for eGFP (green) and mKate2 (red) are shown for $d = 2$ (light) and $d = 22$ (dark). **a** The repressors act as monomers ($n = 1$, $m = 1$) together with $\alpha_y = \alpha_z = 500\,\mathrm{h}^{-1}$. **b** The repressors act as dimers ($n = 2$, $m = 2$), but production rate constants are not balanced ($\alpha_y = 500\,\mathrm{h}^{-1}$, $\alpha_z = 2500\,\mathrm{h}^{-1}$).

3.3 Typical range of model parameters

The typical range for the dissociation constant K for some of the most common transcription factors is approximately 10 nM $\leq K \leq$ 100 μM (*14*). As the typical volume of *E. coli* cells is $V \approx 0.86 \times 10^{-18}$ m^3 (*23*), the concentration of a single molecule is approximately

$$\frac{\frac{1}{6\times10^{23}\ \mathrm{mol}^{-1}}}{0.86 \times 10^{-18}\ \mathrm{m}^3} \approx 2 \times 10^{-6}\ \mathrm{mol/m}^3 = 2\ \mathrm{nM},$$

thus $D = 2$ nM is the concentration of single-copy plasmids. Considering (*23–25*), the total number of proteins in *E. coli* is reported to be approximately 2–4 million per cell volume, and given the proteome size of *E. coli*, this represents approximately 1000 copies per cell for the average protein (*25–27*), corresponding to the concentration $x \approx 2000$ nM. With production and degradation rate constants α and γ, respectively, this yields the estimate $\alpha/\gamma = x/D \approx 1000$ at the steady state of $\dot{x} = \alpha D - \gamma x$. Considering the growth rate $\gamma \approx 1$ h^{-1} typically observed in our experiments (Figure 4), since the dynamic range of protein expression can span up to six orders of magnitude (*27*), the values featured in Supplementary Fig. 104–Supplementary Fig. 106 are expected to fall well within the typical range in *E. coli*.

References

1. Segall-Shapiro, T. H., Sontag, E. D. & Voigt, C. A. Engineered promoters enable constant gene expression at any copy number in bacteria. *Nature biotechnology* **36**, 352–358 (2018).

2. Andersen, J. B. *et al.* New unstable variants of green fluorescent protein for studies of transient gene expression in bacteria. *Applied and environmental microbiology* **64**, 2240–2246 (1998).

3. Cardinale, S., Joachimiak, M. P. & Arkin, A. P. Effects of genetic variation on the e. coli host-circuit interface. *Cell reports* **4**, 231–237 (2013).

4. Chattoraj, D. K. Control of plasmid dna replication by iterons: no longer paradoxical. *Molecular microbiology* **37**, 467–476 (2000).

5. Ratelade, J. *et al.* Production of recombinant proteins in the lon-deficient bl21 (de3) strain of escherichia coli in the absence of the dnak chaperone. *Applied and environmental microbiology* **75**, 3803–3807 (2009).

6. Steel, H., Habgood, R., Kelly, C. L. & Papachristodoulou, A. In situ characterisation and manipulation of biological systems with chi.bio. *PLOS Biology* **18**, 1–12 (2020).

7. Cui, S. *et al.* Multilayer genetic circuits for dynamic regulation of metabolic pathways. *ACS Synthetic Biology* **10**, 1587–1597 (2021). PMID: 34213900.

8. Tas, H., Grozinger, L., Stoof, R., de Lorenzo, V. & Goñi-Moreno, Á. Contextual dependencies expand the re-usability of genetic inverters. *Nature communications* **12**, 1–9 (2021).

9. Rousset, F. *et al.* Genome-wide crispr-dcas9 screens in e. coli identify essential genes and phage host factors. *PLoS genetics* **14**, e1007749 (2018).

10. Nielsen, A. A. *et al.* Genetic circuit design automation. *Science* **352** (2016).

11. St-Pierre, F. *et al.* One-step cloning and chromosomal integration of dna. *ACS synthetic biology* **2**, 537–541 (2013).

12. Lee, J. W. *et al.* Creating single-copy genetic circuits. *Molecular cell* **63**, 329–336 (2016).

13. Lugagne, J.-B. *et al.* Balancing a genetic toggle switch by real-time feedback control and periodic forcing. *Nature communications* **8**, 1–8 (2017).

14. Meyer, A. J., Segall-Shapiro, T. H., Glassey, E., Zhang, J. & Voigt, C. A. Escherichia coli "marionette" strains with 12 highly optimized small-molecule sensors. *Nature chemical biology* **15**, 196–204 (2019).

15. Lou, C., Stanton, B., Chen, Y.-J., Munsky, B. & Voigt, C. A. Ribozyme-based insulator parts buffer synthetic circuits from genetic context. *Nature biotechnology* **30**, 1137–1142 (2012).

16. Chen, Y.-J. *et al.* Characterization of 582 natural and synthetic terminators and quantification of their design constraints. *Nature methods* **10**, 659–664 (2013).

17. Iverson, S. V., Haddock, T. L., Beal, J. & Densmore, D. M. Cidar moclo: improved moclo assembly standard and new e. coli part library enable rapid combinatorial design for synthetic and traditional biology. *ACS synthetic biology* **5**, 99–103 (2016).

18. Chen, Y., Kim, J. K., Hirning, A. J., Josić, K. & Bennett, M. R. Emergent genetic oscillations in a synthetic microbial consortium. *Science* **349**, 986–989 (2015).

19. Zhang, J. *et al.* Binding site profiles and n-terminal minor groove interactions of the master quorum-sensing regulator luxr enable flexible control of gene activation and repression. *Nucleic acids research* **49**, 3274–3293 (2021).

20. Kwak, Y. M. *et al.* Crystal structure of the vanr transcription factor and the role of its unique α-helix in effector recognition. *The FEBS journal* **285**, 3786–3800 (2018).

21. Nielsen, A. A. K. *et al.* Genetic circuit design automation. *Science* **352**, aac7341–aac7341 (2016).

22. Gardner, T. S., Cantor, C. R. & Collins, J. J. Construction of a genetic toggle switch in E. coli. *Nature* **403**, 339–42 (2000).

23. Milo, R. What is the total number of protein molecules per cell volume? A call to rethink some published values. *BioEssays* **35**, 1050–1055 (2013).

24. Neidhardt, F. C. & Curtiss, R. (eds.) *Escherichia coli and Salmonella: cellular and molecular biology* (ASM Press, Washington, D.C, 1996), 2nd ed edn.

25. Arike, L. *et al.* Comparison and applications of label-free absolute proteome quantification methods on Escherichia coli. *Journal of Proteomics* **75**, 5437–5448 (2012).

26. Lu, P., Vogel, C., Wang, R., Yao, X. & Marcotte, E. M. Absolute protein expression profiling estimates the relative contributions of transcriptional and translational regulation. *Nature Biotechnology* **25**, 117–124 (2007).

27. Soufi, B., Krug, K., Harst, A. & Macek, B. Characterization of the E. coli proteome and its modifications during growth and ethanol stress. *Frontiers in Microbiology* **6** (2015).
