# Supplementary material for: Inducible plasmid copy number control for synthetic biology in commonly used E. coli strains
Source: Nat Commun. 2022 Nov 5;13:6691. doi: 10.1038/s41467-022-34390-7 (PMC9637173; doi:10.1038/s41467-022-34390-7)
Supplement: Supplementary file 2 — Reporting Summary [file 41467_2022_34390_MOESM2_ESM.pdf]

nature portfolio

Corresponding author(s): Andras Gyorgy

Last updated by author(s): Oct 18, 2022

Reporting Summary

Nature Portfolio wishes to improve the reproducibility of the work that we publish. This form provides structure for consistency and transparency in reporting. For further information on Nature Portfolio policies, see our Editorial Policies and the Editorial Policy Checklist.

Statistics

For all statistical analyses, confirm that the following items are present in the figure legend, table legend, main text, or Methods section.

n/a	Confirmed	
☐	☒	The exact sample size (n) for each experimental group/condition, given as a discrete number and unit of measurement
☐	☒	A statement on whether measurements were taken from distinct samples or whether the same sample was measured repeatedly
☒	☐	The statistical test(s) used AND whether they are one- or two-sided<br>*Only common tests should be described solely by name; describe more complex techniques in the Methods section.*
☒	☐	A description of all covariates tested
☒	☐	A description of any assumptions or corrections, such as tests of normality and adjustment for multiple comparisons
☒	☐	A full description of the statistical parameters including central tendency (e.g. means) or other basic estimates (e.g. regression coefficient) AND variation (e.g. standard deviation) or associated estimates of uncertainty (e.g. confidence intervals)
☒	☐	For null hypothesis testing, the test statistic (e.g. F, t, r) with confidence intervals, effect sizes, degrees of freedom and P value noted<br>*Give P values as exact values whenever suitable.*
☒	☐	For Bayesian analysis, information on the choice of priors and Markov chain Monte Carlo settings
☒	☐	For hierarchical and complex designs, identification of the appropriate level for tests and full reporting of outcomes
☒	☐	Estimates of effect sizes (e.g. Cohen's d, Pearson's r), indicating how they were calculated

Our web collection on statistics for biologists contains articles on many of the points above.

Software and code

Policy information about availability of computer code

Data collection	The AttuneTM NxT Software (version 3.1.1243.0) was used for acquisition of flow cytometry data from the Biosystems Attune NxT Flow Cytometer. BD FACSDiva Software (version 7.0) was used for the acquisition of flow cytometry data from the BD FACSAria III Sorter. Gen5 Software (version 3.10.06) was used to for aquistion of microplate reader experiment data from the BioTek Cytation 5. The Design & Analysis Software (version 2.5.0) was used for acquisition of quantitative PCR (qPCR) experiment data from the Biosystems Quant Studio 7 Pro. Chi.Bio Operating Software (version 2.3) was used to control and acquire data from the Chi.Bio Turbidostats (Manufactured by LabMaker).
Data analysis	For data analysis and plotting the results, Python (version 3.8.5) was used in the JupyterLab interface (version 2.2.6), provided in the Anaconda package (version 1.7.2). Inkscape (version 1.0.2) and Adobe Illustrator (version 26.5) were used for creating figures. Microsoft Word for Mac (version 16.62) was used to create the tables presented in the Supplementary Information. Microsoft Excel for Mac (version 16.62) was used to analyze the qPCR data, the results were then plotted using Python (version 3.8.5). Mathematical simulations in the Supplementary Information were created and plotted using MATLAB (version R2022a). Overleaf (version v2) online LaTeX editor was used to prepare and compile the manuscript. Benchling (2020-2022, retrieved from https://benchling.com) was used for all visualization of genetic sequences, i.e., plasmid maps and genomes maps, and the built-in Benchling CRISPR guide design tool was used to design optimum gRNAs for dCas9. Primer3Plus (version 2.6.1) was used to design primers for qPCR experiments.

For manuscripts utilizing custom algorithms or software that are central to the research but not yet described in published literature, software must be made available to editors and reviewers. We strongly encourage code deposition in a community repository (e.g. GitHub). See the Nature Portfolio guidelines for submitting code & software for further information.

Data

Policy information about availability of data

All manuscripts must include a data availability statement. This statement should provide the following information, where applicable:

- Accession codes, unique identifiers, or web links for publicly available datasets
- A description of any restrictions on data availability
- For clinical datasets or third party data, please ensure that the statement adheres to our policy

Source data are provided with this paper.

Field-specific reporting

Please select the one below that is the best fit for your research. If you are not sure, read the appropriate sections before making your selection.

☒ Life sciences ☐ Behavioural & social sciences ☐ Ecological, evolutionary & environmental sciences

For a reference copy of the document with all sections, see nature.com/documents/nr-reporting-summary-flat.pdf

Life sciences study design

All studies must disclose on these points even when the disclosure is negative.

Sample size	No calculations were performed to determine the sample size. It is generally accepted that three biological replicates are sufficient for inferential analysis.
Data exclusions	No data were excluded from the analysis.
Replication	Each experimental condition was replicated in biological triplicates in every given experiment, and sufficient agreement was found across all data points in each experimental condition, these are reported as the average (mean) and standard deviation across all biological replicates from their respective experimental measurements.
Randomization	Colonies were selected randomly from agar plates when being prepared for experimental pre-cultures, a single colony represents one biological replicate.
Blinding	Our experiments do not include humans or animals as subjects, where their perception of the experiment could bias the outcome of the study, nor could the experimentalist have influenced the outcome of the experiment through influential bias. Therefore, blinding was not relevant for our study focusing on bacterial gene expression and growth dynamics.

Reporting for specific materials, systems and methods

We require information from authors about some types of materials, experimental systems and methods used in many studies. Here, indicate whether each material, system or method listed is relevant to your study. If you are not sure if a list item applies to your research, read the appropriate section before selecting a response.

Materials & experimental systems

n/a	Involved in the study	
☒	☐	Antibodies
☒	☐	Eukaryotic cell lines
☒	☐	Palaeontology and archaeology
☒	☐	Animals and other organisms
☒	☐	Human research participants
☒	☐	Clinical data
☒	☐	Dual use research of concern

Methods

n/a	Involved in the study	
☒	☐	ChIP-seq
☐	☒	Flow cytometry
☒	☐	MRI-based neuroimaging

Flow Cytometry

Plots

Confirm that:

- [x] The axis labels state the marker and fluorochrome used (e.g. CD4-FITC).
- [x] The axis scales are clearly visible. Include numbers along axes only for bottom left plot of group (a 'group' is an analysis of identical markers).
- [x] All plots are contour plots with outliers or pseudocolor plots.
- [x] A numerical value for number of cells or percentage (with statistics) is provided.

Methodology

Sample preparation	Samples were prepared and processed in PBS.
Instrument	Biosystems Attune NxT Flow Cytometer and BD FACSAria III Sorter
Software	Attune™ NxT Software (version 3.1.1243.0) and BD FACSDiva Software (version 7.0)
Cell population abundance	10,000 gated events per sample.
Gating strategy	Cells were gated according to forward scatter height (FSC-H) and side scatter height (SSC-H).

- [x] Tick this box to confirm that a figure exemplifying the gating strategy is provided in the Supplementary Information.
